# Eight high-quality genomes reveal pan-genome architecture and ecotype differentiation of *Brassica napus*

**DOI:** 10.1038/s41477-019-0577-7

**Published:** 2020-01-13

**Authors:** Jia-Ming Song, Zhilin Guan, Jianlin Hu, Chaocheng Guo, Zhiquan Yang, Shuo Wang, Dongxu Liu, Bo Wang, Shaoping Lu, Run Zhou, Wen-Zhao Xie, Yuanfang Cheng, Yuting Zhang, Kede Liu, Qing-Yong Yang, Ling-Ling Chen, Liang Guo

**Affiliations:** 1 National Key Laboratory of Crop Genetic Improvement, Huazhong Agricultural University, Wuhan, People’s Republic of China; 2 Hubei Key Laboratory of Agricultural Bioinformatics, College of Informatics, Huazhong Agricultural University, Wuhan, People’s Republic of China

**Keywords:** Genetics, Plant sciences, Agriculture

## Abstract

Rapeseed (*Brassica napus*) is the second most important oilseed crop in the world but the genetic diversity underlying its massive phenotypic variations remains largely unexplored. Here, we report the sequencing, de novo assembly and annotation of eight *B. napus* accessions. Using pan-genome comparative analysis, millions of small variations and 77.2–149.6 megabase presence and absence variations (PAVs) were identified. More than 9.4% of the genes contained large-effect mutations or structural variations. PAV-based genome-wide association study (PAV-GWAS) directly identified causal structural variations for silique length, seed weight and flowering time in a nested association mapping population with ZS11 (reference line) as the donor, which were not detected by single-nucleotide polymorphisms-based GWAS (SNP-GWAS), demonstrating that PAV-GWAS was complementary to SNP-GWAS in identifying associations to traits. Further analysis showed that PAVs in three *FLOWERING LOCUS C* genes were closely related to flowering time and ecotype differentiation. This study provides resources to support a better understanding of the genome architecture and acceleration of the genetic improvement of *B. napus*.

## Abstract (web summary)

The assembly of eight high-quality rapeseed genomes allows identification of presence and absence variations (PAVs) and small variations. PAV-based genome-wide association analysis uncovered causal variations for agronomic traits and ecotype differentiation.

## Main

The species *Brassica napus* (AACC, 2*n* = 38) is an economically important oilseed crop that provides approximately 13–16% of vegetable oil globally^1^. *B. napus* originated in the Mediterranean region about 7,500 years ago by natural hybridization between two diploid progenitors, *B. rapa* (AA, 2*n* = 20) and *B. oleracea* (CC, 2*n* = 18)^2,3^. The genetic pool of *B. napus* has been broadened by the introgression of genes from *B. rapa* and synthetic materials produced by artificial crossing between the two diploid progenitors^4,5^. Driven by responses to seasonal changes, such as vernalization requirements, winter hardiness and photoperiod-responsive flowering, *B. napus* has been domesticated with various characteristics^2^. Currently, three ecotypes of *B. napus* are widely distributed in Europe, Asia, Australia and North America^5^. Winter-type oilseed rapes (WORs) were first cultivated in Europe and have been suggested to be the original form of *B. napus*^3^. After being introduced into China, Australia and North America in the twentieth century, cultivated *B. napus* has undergone adaptive changes under a combination of natural and artificial selection to suit different geographical environments and climates. Two additional ecotypes, namely, semi-winter oilseed rapes (SWORs) and spring-type oilseed rapes (SORs)^6,7^, adapted to different vernalization times and temperatures, were gradually formed.

*B. napus* is an allotetraploid crop with a complex genome; there are extensive genomic and phenotypic variations in different accessions and ecotypes^2,5^. The genomes of several rapeseed accessions, including two WORs (Darmor-*bzh*^2^ and Tapidor^8^) and two SWORs (ZS11, ref. ^4^ and NY7, ref. ^5^), have been decoded, providing useful resources for genetic studies such as gene mapping and cloning. However, the current *B. napus* genomes were primarily assembled on the basis of 454 GS-FLX + Titanium and Sanger sequence, next-generation sequencing (NGS) data or medium-coverage PacBio single-molecule real-time (SMRT) sequencing data. Their accuracy and completeness are unsatisfactory for identifying structural variations (SVs), which are major contributors to genetic diversity and play key roles in the determination of agronomic traits in many crop species^9,10^. The concept of the pan-genome was proposed to represent a repertoire of genes including the core genes and dispensable genes of a species^11^. Pan-genomes have been constructed on the basis of NGS technologies for major crops, including soybean, maize, rapeseed and rice, using different numbers of individuals^9,12–14^. These pan-genomes play important roles in the identification of SVs, including copy number variants (CNVs) and presence and absence variations (PAVs) that are associated with crop agronomic traits^10^.

Although several genomes are available for *B. napus*, they cannot represent these genetic variations or satisfy the needs of subsequent functional genomics research and molecular breeding of *B. napus*. Multiple high-quality reference genomes representing different ecotypes are necessary for a better understanding of the genome structure and genetic basis of morphotype differentiation in *B. napus*. In this study, ZS11 de novo assemblies were created by integrating PacBio, Hi-C and BioNano data; the other seven accessions were obtained by integrating high-coverage PacBio and Illumina data; two of them were verified by Hi-C or BioNano data. We performed a genome-wide comparative analysis of these eight well-assembled genomes and the Darmor-*bzh* genome and identified the core-gene clusters, dispensable gene clusters and specific gene clusters. As a proof of concept for the importance of the pan-genome, we identified the causal PAVs that control silique length, seed weight and flowering time of oilseed rape.

## Results

### De novo assembly and annotation of eight *B. napus* genomes

**Fig. 1:**
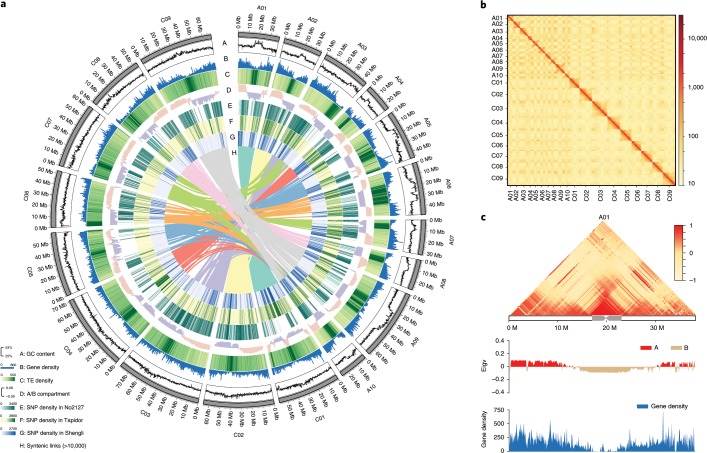
Features of the *B. napus* genome. **a**, Circos plot of the multidimensional topography for *B. napus* ZS11 genome. A–H, Concentric circles from outermost to innermost, show GC content (A), gene density (B), TE density (C), A/B compartment (D), SNP density in No2127 (E), SNP density in Tapidor (F), SNP density in Shengli (G) and syntenic regions between the A and C subgenomes (H). **b**, Genome-wide contact matrix of ZS11 genome. The colour intensity represents the frequency of contact between two 500 kb loci. **c**, Interaction frequency, A/B compartment and gene density in ZS11 chromosome A01. The colour scale represents the Pearson’s correlation coefficient of normalized interaction matrix. Eigv, eigenvector value of correlation matrix.

Eight oilseed rape lines, including four SWORs (ZS11, Gangan, Zheyou7 and Shengli), two WORs (Tapidor and Quinta) and two SORs (Westar and No2127, ref. ^15^, a synthetic line), were sequenced with the PacBio SMRT platform (Supplementary Fig. 1 and Supplementary Table 1). We generated 64.5–97.1 gigabase (Gb) subreads for the eight lines, with an estimated coverage depth of 64−96X for the different genomes (Supplementary Table 2) and the assembled contig N50 was in the range of 2.1−3.1 megabases (Mb) (Supplementary Table 3). The contigs of ZS11 were corrected, ordered and oriented using Hi-C data^16^ and clustered into 19 chromosomes (Fig. 1a,b, Supplementary Fig. 2 and Supplementary Table 4). The other accessions were oriented on the basis of the ZS11 reference genome (see Methods). The different chromosome size of eight *B. napus* genomes might be caused by homologous exchange (HE) events (Supplementary Fig. 3). Our *k*-mer analysis suggested a genome size of 1,200–1,280 Mb for each genome (Supplementary Table 5), which is close to the estimated genome size of *B. napus* (~1,132 Mb) according to flow cytometry analysis^17^. Finally, we obtained eight *B. napus* reference genomes with scaffolds N50 of 50.90−57.88 Mb and genome lengths of 1,001−1,033 Mb. For the ZS11 reference genome, 960.8 Mb (95.3%) of sequence was anchored to 19 chromosomes (Table 1 and Supplementary Table 6). These indicator values are greatly improved compared with those of previously *B. napus* genomes^2,4,5,8^.

**Table 1:** Statistics of genomic assembly and annotation for eight *B. napus* genomes

Accession	Assembly size (Mb)	Anchored chromosome (Mb)	Scaffold N50 (Mb)	TEs (%)	Number of annotated genes	Completeness (%, CEGMA)
Westar	1,007	942	55.24	55.78	97,514	98.79
No2127	1,011	932	53.90	56.86	95,385	99.19
Zheyou7	1,015	924	50.90	56.39	96,209	99.19
Gangan	1,033	934	46.68	56.58	96,843	99.19
Shengli	1,001	930	52.53	56.29	94,586	99.19
Tapidor	1,014	932	52.41	56.59	96,117	99.19
Quinta	1,003	933	55.90	56.29	95,492	98.79
ZS11	1,008	961	57.88	55.62	100,919	99.19

The completeness of all eight assembled genomes exceeded 98% (Supplementary Table 7) when evaluated using BUSCO^18^ and CEGMA^19^. The centromere sequences were successfully identified for all chromosomes of ZS11 (Supplementary Table 8). The accuracy and completeness of the assembly were further supported by high matches with 11 BAC sequences, paired-end short reads of 13 RNA-seq libraries and Illumina genomic paired-end short reads of each accession (Supplementary Tables 9–11). We aligned 8,858 and 5,722 paired BAC-end sequences (BESs) from the ZS11 and Tapidor BAC libraries, respectively, to the assembled ZS11 genome. The average distances between paired BESs were within the range of the estimated insertion sizes, suggesting the high-quality of the genome assembly (Supplementary Table 12 and Supplementary Fig. 4). The genetic map with 7,158 single-nucleotide polymorphism (SNP) markers derived from the cross of *B. napus* ZS11 and Quantum showed a good collinearity with the physical map of ZS11 (Supplementary Fig. 5). In addition, the pseudo-chromosomes of No2127 generated by Hi-C data showed good synteny with those based on the ZS11 genome (Supplementary Fig. 6). The scaffolds assembled by BioNano optical maps for No2127 and Westar covered ~66% of the reference genomes (Supplementary Table 13) and were highly consistent with the 19 chromosomes, indicating the accuracy of contig orientation (Supplementary Figs. 7 and 8). Only 86 and 99 conflicts were identified between the assembled genomes and BioNano optical maps for No2127 and Westar, respectively (Supplementary Tables 14 and 15).

The genome assemblies are highly improved in regions containing highly repetitive sequences. In total, 56.8−58.2% transposable elements (TEs) were identified in the eight assembled genomes (Fig. 1a and Supplementary Table 16), which is much higher than the proportion in the published Darmor-*bzh* genome^2^ (34.8%), further supporting the completeness of the genome assembly. Long terminal repeat (LTR)/Gypsy repeat elements were the most abundant, accounting for ~16% of the genome, followed by LTR/Copia. Overall, the proportion of retrotransposons (RTs, ~36%) was much higher than that of DNA transposons (~10%). We identified 24,704 intact LTR-RTs from the ZS11 genome and estimated their insertion times (substitution rate 1.5 × 10^−8^)^20^. The insertions of 93% of the intact LTR-RTs occurred after the differentiation of *B. rapa* and *B. oleracea*. The intact LTR-RTs in the C subgenome continuously amplified over 3 Myr since the differentiation of *B. oleracea* and *B. rapa*, resulting in a wide distribution of LTR-RTs in euchromatic regions and a larger genome size than the A subgenome. However, the A subgenome had expanded within the last 1 Myr (Supplementary Fig. 9), similar to the expansion observed in the genomes of its diploid progenitors^21,22^, indicating that there was a higher proportion of active LTR-RTs in the A subgenome.

The remaining unmasked *B. napus* genome was annotated using a comprehensive strategy combining evidence-based and ab initio gene prediction. Using PASA and the EVM pipeline^23^, we incorporated protein sequences from six related species and the transcripts assembled from the RNA-seq data of each accession. We identified 94,586–100,919 gene models in the eight genomes, with an average coding sequence length of ~1 kilobase (kb) and an average of five exons per gene (Supplementary Table 17). More than 97% of the annotated genes are supported by homology to known proteins or functional domains in other species (Supplementary Table 18). On a genome-wide scale, protein-coding genes tend to be distributed in chromosome arms with lower GC content and fewer repeat elements (Fig. 1a). In addition, 21,430−27,390 non-coding RNAs, including ribosomal RNAs (rRNAs), transfer RNAs (tRNAs), microRNAs (miRNAs) and small nuclear RNAs (snRNAs), were annotated in each of the eight *B. napus* genomes (Supplementary Table 19).

As shown in Fig. 1b, the Hi-C data of all the ZS11 chromosomes showed a strong signal on the main diagonal, indicating frequent interactions between adjacent loci. Strong intrachromosomal interactions were also observed between chromosome arms, which were consistent with the chromosome concept, in which each chromosome occupies a limited, exclusive nuclear space^24^. The Hi-C map of *B. napus* showed similar A/B compartment characteristics to mammal genomes^16^, where the B compartment is concentrated in the centromere region, with higher transposon density and the A compartment is mainly distributed on the chromosome arm with higher gene density (Fig. 1c).

### Phylogenetic analysis of *B. napus* and other *Brassica* species

**Fig. 2:**
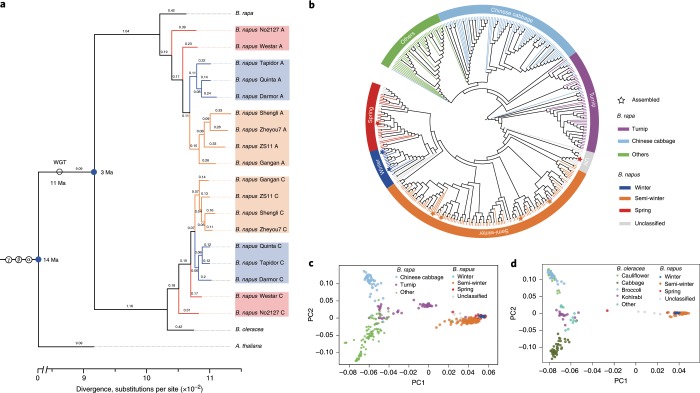
Phylogenetic analysis of *Brassicaceae*. **a**, Phylogenetic relationship of nine *B. napus* genomes and their diploid progenitors, *B. rapa* and *B. oleracea*. The phylogenetic tree is constructed on the basis of 1,235 conserved genes. The values on the branch are the substitutions between species and the nearest ancestor. WGT, whole genome triplication. **b**, A neighbour-joining tree of 210 *B. napus* accessions, eight assembled accessions and 199 *B. rapa* accessions. Each assembled accession was represented by a pentagram (left to right: Westar, Quinta, Tapidor, Shengli, Zheyou, Gangan, ZS11 and No2127). The layer rings indicate the group name of each clade. **c**, PCA plot of *B. napus* (*n* = 210) and *B. rapa* (*n* = 199) accessions. **d**, PCA plot of *B. napus* (*n* = 210) and *B. oleracea* (*n* = 119) accessions.

*Brassica* has undergone lineage-specific whole-genome triplication and the *B. napus* genome was formed via allotetraploidization of its diploid progenitors *B. rapa* and *B. oleracea* (Fig. 2a and Supplementary Fig. 10a). In the ZS11 genome, up to six homologous copies can be found in a single synteny block, which indicated traces of paralogue retention following triploidization and allotetraploidization (Supplementary Fig. 10b). On the basis of the coding sequences of 1,235 single-copy orthologous genes, we constructed a *Brassica* phylogeny for the 20 subgenomes/genomes with *A. thaliana* as the outgroup. The structure of the phylogenetic tree clearly indicates that two subgenomes of the resynthesized allotetraploid No2127 are the closest to the two ancestral genomes of *B. rapa* and *B. oleracea* and rapeseed lines of the same *B. napus* ecotypes clustered together (Fig. 2a). Synonymous substitution rate (*K*_s_) analysis indicated that *B. napus* differentiated from its diploid progenitors, *B. rapa* and *B. oleracea*, ~10,000 years ago (Supplementary Fig. 11). *B. rapa* and *B. oleracea* differentiated ~3 million years ago (Ma), which is close to a previous estimation based on SLR1 genes^25^. A whole-genome triplication of *Brassica* species occurred ~11 Ma and *A. thaliana* and *B. napus* differentiated ~14 Ma. The triplicated regions of the 19 chromosomes of ZS11 were reconstructed according to the relative positions of the 22 ancestral crucifer karyotype blocks (A–X) in *A. thaliana*^26^ (Supplementary Fig. 12). These syntenic blocks occupy almost the whole-genome assemblies of the A (43,966 of 44,359 genes) and C subgenomes (52,067 of 52,562 genes) in ZS11 (Supplementary Table 20). On the basis of the high-quality ZS11 reference genome, we identified 297 MADS-box genes, which is more than previous studies^27^. Some MADS-box genes have been shown to be targets of domestication and variety improvement^28^. We identified 28 MADS-box genes that had undergone positive selection in the ZS11 genome, including *BnaA10.FLC*, which is closely related to flowering time and reproductive development (Supplementary Fig. 13 and Supplementary Table 21).

To study the origins of the *B. napus* subgenomes, we analysed SNPs in the A subgenomes of 210 *B. napus* accessions^1^, the eight accessions assembled in this study and 199 *B. rapa* accessions covering most subspecies of *B. rapa*^29^. We constructed a neighbour-joining tree for the A subgenomes of *B. napus* and found that all the A subgenomes were rooted in the common ancestor turnip (Fig. 2b), indicating that the A subgenomes may derive from turnip, in agreement with a previous study^30^. Principal component analysis (PCA) also located *B. napus* close to turnip accessions (Fig. 2c), supporting the results of the phylogenetic tree. The 210 *B. napus* accessions included SWORs from China and WORs and SORs from Europe, Canada, Australia and other countries. SWORs were closer to turnip than to SORs and WORs in the phylogenetic tree. PCA also showed that SWORs were close to turnip (Fig. 2c).

Similarly, a neighbour-joining tree of the 210 *B. napus* and 119 *B. oleracea* accessions^29^ was constructed using tag SNPs in the C subgenome. However, the origin of the C subgenome in *B. napus* was not robustly inferred (Fig. 2d and Supplementary Fig. 14), which is consistent with previous results that the C subgenome in *B. napus* originated during the divergence of the ancestors of four *B. oleracea* subspecies^3^. The 210 *B. napus* lines were classified into different subclades that roughly corresponded to three ecotypes. Of the eight sequenced rapeseed lines, ZS11, Gangan, Shengli and Zheyou7 were clustered with the SWORs, Quinta and Tapidor with the WORs, Westar with the SORs and the artificial synthesized line No2127 with the unknown type. The node positions of the eight reference accessions in the phylogenetic tree of the *B. napus* A subgenome were similar to those in the C subgenome tree. This result illustrated that the genetic diversity of these eight accessions had good typicality or representativeness of natural rapeseed populations.

### Large genomic variations in eight genomes of *B. napus*

To compare the genomic differences among the eight assembled *B. napus* genomes, we aligned the other seven genomes to ZS11. In general, 76−84% of the ZS11 genome sequences were in one-to-one syntenic blocks to the other seven genomes, with the lowest ratio found in the resynthesized line No2127 (Supplementary Figs. 15 and 16). In the aligned syntenic regions, 1.87−3.93 × 10^6^ high-quality SNPs and 0.98−1.48 × 10^6^ small insertions/deletions (InDels) were identified (Supplementary Tables 22 and 23), with an average of 2.24−5.18 SNPs and 1.17−1.95 InDels per kb in different *B. napus* genomes. The distributions of SNPs and InDels were positively correlated (Supplementary Fig. 17) and both were more abundant in intergenic regions. The number of synonymous SNPs was much larger than that of non-synonymous SNPs and InDels were more abundant with multiples of 3 bp in coding regions, as expected, because such InDels do not cause frameshifts (Supplementary Fig. 18).

The high-quality reference genomes allowed us to identify large SVs by comparative genomic analysis between different accessions. Compared with the ZS11 genome, 7.5−15.6 Mb were identified as inversions in each of the other seven accessions, with 40 large inversions of >50 kb (Supplementary Fig. 19 and Supplementary Tables 24 and 25). We also detected 39.7−49.1 Mb translocations, with more interchromosomal translocations (2,343−5,957) than intrachromosomal translocations (1,157−2,149). Most of the translocations were caused by HE events (Supplementary Tables 26 and 27). We further characterized PAVs by comparing the ZS11 reference genome with the other seven genomes. In total, 16,720−34,158 regions with cumulative lengths of 77.2−149.6 Mb were identified as absent in the ZS11 genome and these regions were associated with 2,619−4,810 genes in the other seven genomes (Supplementary Tables 28–35). Gene ontology enrichment analysis showed that the genes in PAV regions were enriched in defence response, signal transduction, response to stress and so on (Supplementary Table 36). In addition to the large SVs, 9.4−14.9% of the genes contained large-effect mutations, including frameshifts, gain or loss of stop codons, or other variations causing major protein differences (Supplementary Table 37). Consequently, extensive intraspecific genomic variations exist and may cause massive phenotypic variations in *B. napus*^31^. The eight high-quality reference genomes provide important resources for capturing the full landscape of genetic diversity in different *B. napus* genomes.

### Pan-genome and gene index of *B. napus*

**Fig. 3:**
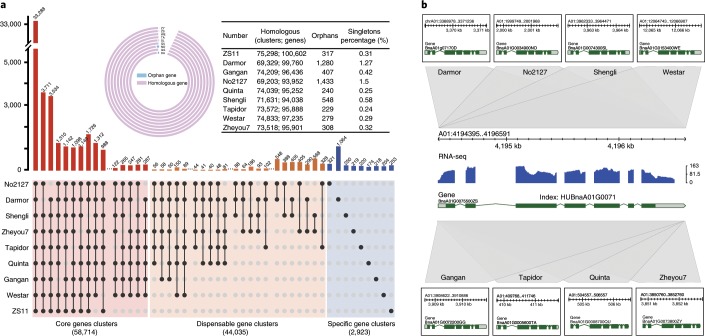
The pan-genome and gene index of nine *B. napus* accessions. **a**, Core- and pan-genome of *B. napus*. The upper circle diagram shows the ratio of homologous genes to orphan genes and the table lists the detailed number. The histograms below show the core-gene clusters (present in seven or more genomes), dispensable gene clusters (present in two to six genomes) and specific gene clusters (present in one genome). **b**, An example of *B. napus* Gene Index. HUBnaA01G007100 is the unique gene ID of A01 *MAPKK2* gene across nine *B. napus* genomes. The axis is the physical location of the gene in the ZS11 genome. The blue column is an accumulation of multi-tissue RNA-seq reads map. Grey blocks are collinearly aligned regions. Annotated gene structure in each genome is in the black box.

Referring to the pan-genome definition in corn^32^ and *B. oleracea*^33^, we constructed the pan-genome of *B. napus* by adding the PAV sequences from the individual genomes to the ZS11 genome and obtained a pan-reference genome with a genome size of ~1.8 Gb and 152,185 genes. The genome size and gene number increased as the number of genomes increased but the number of orthologous gene clusters did not increase after combining six genomes (Supplementary Fig. 20), indicating that the gene families in the *B. napus* pan-reference genome tend to be saturated when integrating six genomes representing the three ecotypes. We further analysed the following four conceptual groups of genes^34^ in nine genomes, including our eight genomes and the Darmor-*bzh* genome: the pan-gene clusters (the cumulative set of all genes), the core-gene clusters (genes present in *n* ≥ 7 genomes containing paralogues), the dispensable gene clusters (genes present in 2−6 genomes containing paralogues) and the specific gene clusters (present in only one genome, including specific gene clusters and singletons). Orthologous and paralogous genes were identified on the basis of OrthoMCL^35^. All 874,105 annotated genes in the nine *B. napus* genomes were assigned to 105,672 gene clusters (group of homologous genes) (Supplementary Table 38). Of these gene clusters, 58,714 (~56%) were core-gene clusters that existed in at least seven genomes and 44,035 (~42%) were dispensable gene clusters. The remaining ~2% (2,923 gene clusters and 5,041 singletons) were specific genes with no orthologues in other genomes. We then performed gene ontogeny enrichment analysis to associate biological functions with the pan-, core- and specific-genes. Generally, basic functions, such as ‘regulation of biosynthetic process’, ‘transport and primary metabolic process’, were enriched in the core-gene clusters of *B. napus* and 86% of the genes in the core genome contained known functional domains. On the other hand, ‘response to stimulation’ or ‘stress and protein phosphorylation’ was enriched in the specific gene clusters of almost all accessions (Supplementary Figs. 21–27). Secondary metabolism and biological process regulatory proteins were often identified as specific genes and thus represent critical genetic differences in *B. napus*. For convenience of gene comparison among different rapeseed lines and retrieval of genes of interest, we constructed a unique gene index in the above nine *B. napus* genomes (Supplementary Table 39). Figure 3b shows an example of the gene index HUBnaA01G0071 in ZS11 and the other eight genomes. The exon–intron structure of HUBnaA01G0071 is conserved in all nine genomes. In the gene indices, 35.5−55.9% of ZS11 genes are identical to genes in the other eight genomes, while 36.7−58.3%, 1.2−2.1% and <0.06% have high identity (>80%), medium identity (50−80%) and low identity (<50%) to genes in the other eight genomes, respectively (Supplementary Table 40). A pan-reference genome coordinate system was constructed (http://cbi.hzau.edu.cn/bnapus) for quick querying, viewing and downloading gene indexes, gene annotations and SVs across different *B. napus* genomes.

### SNP-based GWAS versus PAV-based GWAS: case studies for silique length, seed weight and flowering time

**Fig. 4:**
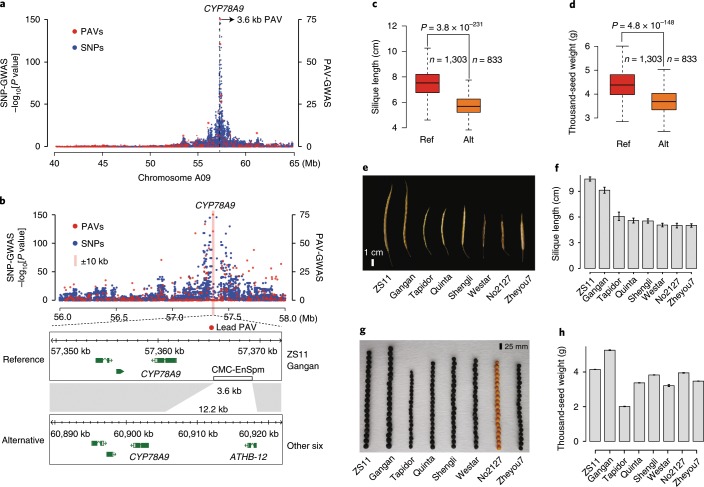
GWAS of silique length and seed weight in the NAM population. **a**, Manhattan plots of SNP-GWAS and PAV-GWAS for silique length. **b**, A 3.6-kb CACTA-like insertion as lead PAV of *BnaA09.CYP78A9* promoter region. **c**, The silique length in lines with different *CYP78A9* alleles. For **a** and **b**, the GWAS (-lmmm 1: Wald test) was performed with 3,971,412 SNPs or 27,216 PAVs in the BN-NAM population containing 2,141 RILs. **d**, Thousand-seed weight in lines with different *CYP78A9* alleles. For **c** and **d**, *P* values were determined using two-tailed Student’s *t*-tests. The middle bars represent the median while the bottom and top of each box represent the 25th and 75th percentiles, respectively. The whiskers extend to 1.5 times the interquartile range. Alt, alternative; Ref, reference. **e**,**f**, Phenotype data of silique length in eight *B. napus* accessions. **g**,**h**, Phenotype data of seed weight in eight assembled *B. napus* accessions. For **e** and **g**, experiments were repeated five times with similar results. For **f** and **h**, data are mean ± s.d. of eight and five biological replicates, respectively.

Many studies show that large SVs have a more significant influence on traits than SNPs^36–38^, while traditional SNP-based genome-wide association studies (SNP-GWASs) have difficulty in detecting missing content in the reference genome. PAVs have been reported as the causal variations of several important traits in *B. napus*^13,39,40^. To explore the contribution of SVs to trait variation, we conducted GWAS on three important yield-related traits, including silique length (SL), seed weight (SW) and flowering time. This was done on the basis of 3,971,412 SNPs and 27,216 PAVs (PAV-GWAS) with minor allele frequencies (MAFs) higher than 0.05 in a *B. napus* nested association mapping (NAM) population. The NAM population was developed by crossing ZS11 with 15 diverse founder inbred lines, including two reference inbred lines, Zheyou7 and Gangan^41^ (Supplementary Tables 41–43). The PAV genotypes of the 16 NAM parental lines were identified by BayesTyper^42^ and projected onto the 2,141 recombinant inbred lines (RILs) on the basis of the high-density genetic linkage map^41^. SNP-GWAS identified 75 and 38 SNPs associated with SL and SW, respectively (Supplementary Table 44). Although the peak SNP on chromosome A09 fell within the previously reported region identified by traditional quantitative trait locus mapping and positional cloning^43,44^, none of the associated SNPs was located in the regulatory region or coding sequence of the target gene *BnaA9.CYP78A9* (Fig. 4a,b). Encouragingly, PAV-GWAS directly detected the 3.9-kb CACTA-like TE inserted upstream of the *BnaA9.CYP78A9* promoter region, which was identified as the causal variation for SL and SW^44^ (Supplementary Figs. 28–29). The NAM individuals containing the CACTA-like TE insertion had longer siliques and larger seeds than those not containing the TE insertion (Fig. 4c–h). Previous studies have demonstrated that the inserted TE acts as an enhancer, promoting the high expression of *BnaA9.CYP78A9* preferentially in the silique valves of varieties with long siliques and large seeds^44^. Among the eight genomes, Gangan and ZS11 had TE insertions upstream of the *BnaA9.CYP78A9* promoter. Consequently, these two lines showed significantly greater SL and higher SW than the others (Fig. 4b,e–h). This result indicates that PAV-GWAS is complementary to SNP-GWAS in identifying associations with phenotypes caused by SVs.

**Fig. 5:**
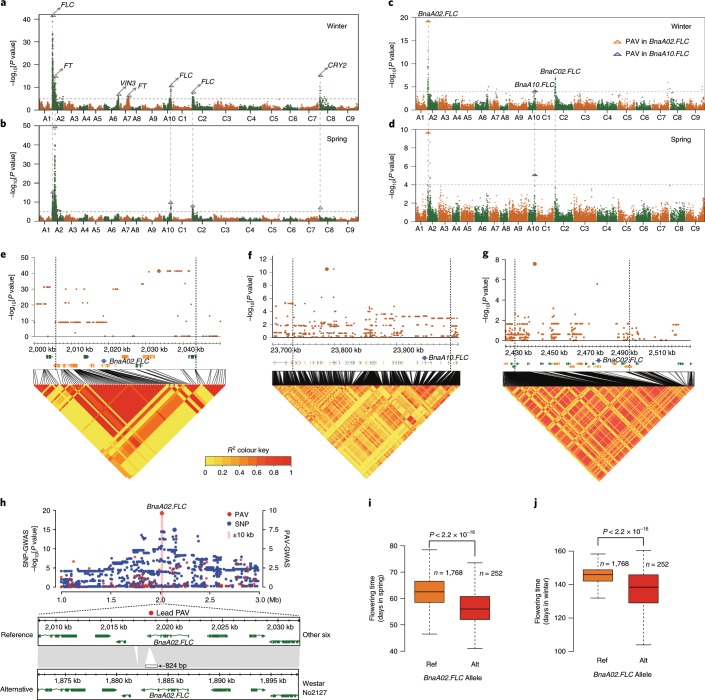
GWAS of flowering time in the NAM population. **a**,**b**, Manhattan plots for flowering time analysed by SNP-GWAS in winter and spring environments, respectively. The gray dashed lines indicate the significance threshold. The BLUP values of the days from sowing to flowering (DTF) in the winter and spring environments were used to represent the flowering time for SNP-GWAS. The triangles and arrows denote the main candidate genes surrounding the strong peaks. **c**,**d**, Manhattan plots for flowering time analysed by PAV-GWAS in winter and spring environments, respectively. The BLUP values of DTFs in winter and spring environments were used to represent the flowering time for PAV-GWAS. The gray dashed lines indicate the significance threshold. **e**–**g**, Local Manhattan plots, gene positions and LD heatmaps show the regions surrounding the strong peaks of the candidate genes (*BnaA02.FLC*, *BnaA10.FLC* and *BnaC02.FLC*) identified by SNP-GWAS. **h**, An 824-bp hAT insertion in the last exon of *BnaA02.FLC* was identified as the lead PAV by PAV-GWAS. For **a**–**h**, the GWAS (-lmmm 1: Wald test) was performed with 3,971,412 SNPs or 27,216 PAVs in the BN-NAM population containing 2,141 RILs. **i**,**j**, Flowering time of lines with different *BnaA02.FLC* alleles in spring (**i**) and winter (**j**), respectively. *P* values were determined using two-tailed Student’s *t*-tests. The middle bars represent the median, while the bottom and top of each box represent the 25th and 75th percentiles, respectively. The whiskers extend to 1.5 times the interquartile range.

Flowering time is a complex agronomic and quantitative trait reflecting the adaptation of *B. napus* to its environment by tailoring vegetative and reproductive growth phases to climatic effects. To dissect the genetic architecture of flowering time, we grew the NAM population in multiple environments, including two spring environments and six winter environments. The flowering time (days from sowing to flowering) showed extensive variation among different RILs (Supplementary Table 45). SNP-GWAS identified 63 and 79 loci associated with flowering time in the winter and spring environments, respectively (Fig. 5a,b and Supplementary Table 46). Most of the loci identified in the spring environment overlapped with those identified in the winter environment. Underlying these loci, 67 genes orthologous to *Arabidopsis* genes controlling flowering time were identified, including three *FLOWERING LOCUS C* (*FLC*), two *FLOWERING LOCUS T* (*FT*), two *VERNALIZATION INSENSITIVE 3* (*VIN3*) and two *CRYPTOCHROME 2* (*CRY2*) genes (Fig. 5a,b and Supplementary Tables 46–47). PAV-GWAS detected only three consistent peaks in both the spring and winter environments. The peak PAVs were directly located within two orthologues of *Arabidopsis FLC*, *BnaA02.FLC* and *BnaA10.FLC* (Fig. 5c,d). The peak PAV (824 bp) on A02 is a partial sequence of hAT retrotransposon located in the sixth exon of *BnaA02.FLC* (Fig. 5h). NAM RILs with this 824-bp PAV flowered much earlier than those without it in both the winter and spring environments (Fig. 5i,j). The peak PAV on A10 was a 4,421-bp hAT inserted in the promoter region of *BnaA10.FLC*, which was not reported in a previous GWAS studies with nearly 1,000 re-sequenced rapeseed accessions^45^. NAM RILs with this hAT insertion flowered later than those without it in spring environments (Supplementary Fig. 30).

### The role of *FLC* genes in the divergence of the three rapeseed ecotypes

**Fig. 6:**
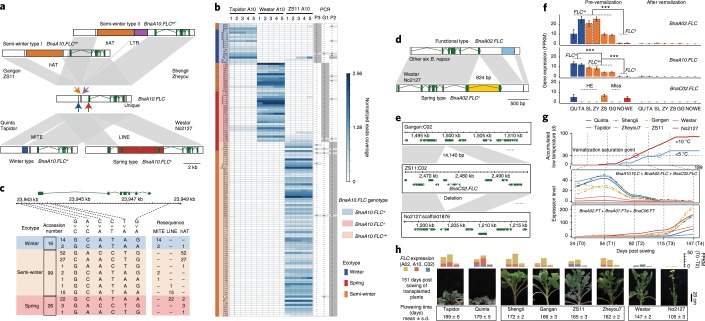
Structural variations detected in *BnaA10.FLC*, *BnaA02.FLC* and *BnaC02.FLC*. **a**, Insertions of four transposable elements around *BnaA10.FLC* in different ecotypes. **b**, Genotyping *BnaA10.FLC* in 141 *B. napus* accessions. The left were ecotypes of *B. napus* accessions. The middle is the read coverage of resequencing data in 15 representative sites, with Tapidor A10: 22,661,433–22,661,437; Westar A10: 23,731,730–23,731,734 and ZS11 A10: 23,942,298–23,942,302. The right is the PCR results statistics of three insertions. **c**, The haplotypes of six SNPs and three TEs around *BnaA10.FLC* in 141 *B. napus* accessions. **d**,**e**, SVs in *BnaA02.FLC* (**d**) and *BnaC02.FLC* (**e**) in eight accessions. **f**, The expression levels of *BnaA02.FLC*, *BnaA10.FLC* and *BnaC02.FLC* in plants before and after vernalization (T0 and T4) based on the number of fragments per kilobase of the exon model per million mapped reads (FPKM). ***P < 0.001 (two-tailed Student’s *t*-test). Error bars indicate the mean ± s.d. (*n* = 2). ns, not spring; w, winter; s, spring; sw, semi-winter. **g**, The relationship between the accumulated days with low temperature, the cumulative expression levels of *FLCs*, *FTs* and flowering time in the 2018–2019 growing season in Wuhan. Three expressed *FT* genes were considered (average FPKM ≥ 1). Accumulated low-temperature curves indicated that the end of vernalization was in T2–T3 for SORs and T3–T4 for WORs. **h**, The cumulative expression levels of three *FLC* genes and the flowering time characterization of eight assembled *B. napus* accessions. Stacked histogram showed *FLCs* expression in T0–T3. These plants were transplanted from the field to the pot at 106 d after sowing. The standard deviation and average of flowering time were counted from 14–21 lines.

*FLC* has been reported as a key transcriptional regulator that delays flowering by repressing the expression of floral integrators such as *FT*, *SUPPRESSOR OF OVEREXPRESSION OF CO1* (*SOC1*) and *FD* in *A. thaliana*^46^. *B. napus* contains multiple copies of *FLC* and several homologues are associated with flowering time variation^47^ (Fig. 5e–g and Supplementary Fig. 31). We compared the gene structures of *BnaA02.FLC*, *BnaA10.FLC* and *BnaC02.FLC* among our eight assembled *B. napus* genomes and their diploid progenitors *B. rapa* and *B. oleracea*. Four TEs were identified in the promoter and coding region of *BnaA10.FLC* (Fig. 6a and Supplementary Figs. 32 and 33). A 5,565-bp LINE transposon was identified only in the first exons of the two SORs (No2127 and Westar), while a 621-bp MITE transposon was identified only in the promoter regions of two WORs (Quinta and Tapidor), although it was also present in the genome of Darmor-*bzh*. This MITE was previously identified as specific to WORs^48^. The 4,421-bp hAT retrotransposon was identified only in the promoter regions of the four SWORs (ZS11, Zheyou7, Gangan and Shengli). In addition, the 1,656-bp LTR was found in the promoter regions of two (Shengli and Zheyou7) out of the four SWORs. We further validated these TEs in 210 *B. napus* accessions^1^ sequenced with an average depth of 7× (Supplementary Table 48), 141 of which had ecotype information^49^. Five characteristic sequence sites were selected from each of the LINE, MITE and hAT insertions to represent the corresponding TEs (Fig. 6b, Supplementary Fig. 34 and Supplementary Notes). Of the 210 *B. napus* accessions, 34 contained the MITE insertion, 60 contained the hAT insertion and 129 contained the LINE insertion (Supplementary Table 49). Notably, all the WORs contained the MITE insertion, 85% (22/26) of the SORs contained the LINE insertion and 81% (80/99) of the SWORs contained the hAT insertion, indicating a strong correlation between specific TE insertions in *BnaA10.FLC* and ecotype classification (Fig. 6b). The above TE insertions were further confirmed by PCR amplification and sequencing using transposon-specific primers (Supplementary Table 50 and Supplementary Fig. 35). We then analysed the haplotypes of six SNPs and the three TEs located within the 5.0-kb upstream and downstream regions and the coding sequence of *BnaA10.FLC* (Fig. 6c). The results showed that the haplotypes of the three TEs were more consistent with ecotype information and flowering time than the haplotypes of the SNPs (Supplementary Fig. 36) and this result was also supported by PCA (Supplementary Fig. 34), suggesting that these TE insertions in *BnaA10.FLC* could be used to roughly classify *B. napus* lines with unknown ecotype information into specific ecotypes, which would be very useful for rapeseed breeding.

We further detected insertions in *BnaA10.FLC* on the basis of the resequencing data of 199 *B. rapa* accessions, including Chinese cabbage and paikoi^29^ and PCR amplification in another 192 oil-use and Chinese cabbage accessions. Of these *B. rapa* lines, 295 (75.4%) contained the 4,421-bp hAT insertion and 25 (6.4%) contained the MITE insertion, while 18.2% of the cabbage lines did not have either of these two insertions, suggesting that the insertion of these two TEs in *BnaA10.FLC* predated the formation of the allotetraploid *B. napus* (Supplementary Fig. 37 and Supplementary Table 51). The SOR-specific LINE transposon was not found in the *B. rapa* accessions analysed in this study, which may be because the insertion of the LINE transposon occurred during the process of domestication and selection of *B. napus* or due to the limited number of *B. rapa* accessions analysed.

The 824-bp hAT inserted in the sixth exon of *BnaA02.FLC* was found in only two SORs and not in the other ecotypes (Fig. 6d and Supplementary Fig. 38). Functional *BnaC02.FLC* genes were found in the genomes of ZS11, Quinta and Westar, while this gene was replaced by *BnaA02.FLC* in the genomes of Shengli, Zheyou7 and Tapidor due to HE events (Supplementary Fig. 39) or was completely deleted in the genomes of Gangan and No2127 (Fig. 6e). Therefore, there are two copies of the *BnaA02.FLC* gene in Tapidor, Zheyou7 and Shengli, one copy of *BnaA02.FLC* and one copy of *BnaC02.FLC* in ZS11, Westar and Quinta, only one copy of *BnaA02.FLC* in Gangan and no *BnaC02.FLC* in No2127 (Supplementary Table 52).

In *B. napus*, late flowering and responsiveness to vernalization correlate with the level of *BnaFLC* messenger RNA (mRNA) transcripts. To further investigate the expression of the *BnaA10.FLC*, *BnaA02.FLC* and *BnaC02.FLC* genes in different ecotypes, we performed transcriptome sequencing using leaves at five stages (T0–T4) started from four-leaf seedlings at one-month intervals and analysed the expression levels of the *FLC* and *FT* genes before and after vernalization. The expression levels of *BnaA02.FLC* and *BnaA10.FLC* before vernalization (T0) displayed significant differences, which were associated with PAVs and copy number, among the eight accessions (Fig. 6f). *BnaA02.FLC* was not expressed in two SORs (Westar and No2127) in any stage, and *BnaC02.FLC* was expressed in only Quinta, ZS11 and Westar. Unlike No2127, Westar has one functional *BnaC02.FLC*, which may explain the later flowering of Westar than of No2127. It has been reported that *BnaA02.FLC* has a stronger flowering repression effect than *BnaC02.FLC*^47^. Although Tapidor and Quinta both possess *BnaA10.FLC*, Tapidor has two copies of *BnaA02.FLC*, while Quinta has one copy of *BnaA02.FLC* and one copy of *BnaC02.FLC*, which may cause the difference in flowering time between them (Fig. 6h). The expression levels of all three *FLCs* decreased after vernalization (T4). Simultaneously, the expression levels of the *FT* genes (*BnaA02.FT, BnaA07.FT* and *BnaC06.FT*) were negatively correlated with those of the *FLCs* (Fig. 6g and Supplementary Figs. 40–41). The decrease in *FLC* transcripts perfectly coincided with the increase in *FT* transcripts (Fig. 6g and Supplementary Fig. 41), which also coincided with the flowering times of the eight accessions (Fig. 6h).

## Discussion

In this study, we performed de novo assembly and annotation of eight representative *B. napus* genomes by integrating data from PacBio sequencing, Illumina paired-end short read sequencing and Hi-C technologies. Our assemblies are highly accurate and complete (Table 1) owing to the long-read sequencing technology and a scaffolding strategy based on chromatin interaction^2,4,5,8^, especially in regions enriched in TEs, such as centromere regions. In addition, this comprehensive comparative genomic analysis of eight *B. napus* genomes illustrates the power of multiple de novo assemblies in detecting genomic variations that have not been found by resequencing alone. Many intraspecific variations, including more than 16,720 PAVs, 1,360 inversions and 3,716 translocations and a series of SNPs and InDels, were revealed among the eight assemblies. We demonstrated that >76% of the genes in the different genomes were located in syntenic regions and >9.4% of genes were associated with large-effect mutations. Thus, these eight reference genomes represented the genetic information of three rapeseed ecotypes and provided useful resources for the identification of genomic variations in different rapeseed lines and for the understanding of genetic diversity and phenotypic heterogeneity.

Previous studies of crop pan-genomes confirmed that a single reference genome does not adequately represent the genetic diversity within a species^9,14,50–52^. Thus, we constructed a high-quality pan-genome of *B. napus* on the basis of the eight de novo assembled genomes and Darmor-*bzh*. The pan-genome reached ~1.8 Gb with 105,672 gene clusters that consisted of ~56% core-gene clusters, ~42% dispensable gene clusters and ~2% specific gene clusters. These results explain why several candidate genes associated with important agronomic traits in *B. napus* cannot be obtained directly by the homologous gene cloning approach from the Darmor-*bzh* reference genome (the fragment with the candidate genes was not present in the genome) but required the strategy of traditional BAC library construction and NGS sequencing^53,54^. The above results also indicate that the construction of the pan-genome is valuable for the study of important functional genes and genetic breeding of *B. napus*. To allow researchers to better use the pan-genome, we constructed a bioinformatics platform to reveal these data, which should greatly accelerate genetic studies and molecular breeding efforts, such as gene mapping, gene cloning, GWAS and marker-assisted selection, in oilseed rape.

Abundant evidence from genetics and molecular biology has clearly demonstrated that SVs can cause major phenotypic variance affecting a series of important agronomic and quality traits in crops^9,10,14,55^ and the characterization of SVs in populations is becoming the frontier of plant genomics. We used PAV-GWAS as a new strategy for screening candidate genes for traits. This technique yielded fewer significant associations than traditional SNP-GWAS but could focus on absent loci in the reference genome and obtain accurate positions. Subsequent analysis also showed that the PAVs among different ecotypes of *B. napus* directly affected the expression of the *FLC* genes and were involved in flowering regulation. Although many SNPs significantly associated with flowering time in *B. napus* were identified on both sides of the *FLC* genes of A02, A10 and C02 in previous studies^5,14,55,56^, such SNPs are often proxies and not the functional variants driving the allelic difference in the *FLC* genes. These SNPs, rather than SVs, in these three *FLC* genes were also identified by sequence capture in four rapeseed accessions with different winter hardiness and vernalization requirements^57^. These results further validate that, compared to SNPs, SVs contribute more strongly to phenotype and the resulting phenotypic consequences.

We identified different types of SVs that altered the expression levels of three *FLC* genes. We can infer that *BnaA10.FLC*, *BnaA02.FLC* and *BnaC02.FLC* all participated in the vernalization response but *BnaA10.FLC* was the master regulator causing the differentiation of the three ecotypes of *B. napus*. Oilseed rape starts to flower when the low-temperature days exceed a critical number and the expression of three *BnaFLCs* and four *BnaFTs* achieve a threshold value (Fig. 6g). Due to the LINE insertion in the first exon of *BnaA10.FLC*, the loss-of-function mutation makes SORs require weak or no vernalization. The MITE insertion in the promoter region of *BnaA10.FLC* enhances the expression of *BnaA10.FLC* which leads to a requirement of strong vernalization for WORs. A demand for vernalization of SWOR is somewhere between the other two ecotypes due to the hAT insertion in the promoter region of *BnaA10.FLC*.

## Methods

### Plant materials

Eight rapeseed accessions of three ecotypes named ZS11, Gangan, Zheyou7, Shengli, Tapidor, Quinta, Westar and No2127 were used in this study. ZS11 and Tapidor are the same as the two cultivars previous sequenced^4,8^. Tapidor and Quinta are two typical WORs from Europe. Westar is an SOR from Canada that is widely used as transgenic receptor. No2127 is an artificially synthesized yellow-seeded SOR derived from hybridization between *B. rapa* and *B. oleracea* in Europe in the 1980s^15^. Zheyou7 and ZS11 are two elite open-pollinated cultivars released in the 1970s and 2000s in China once or being widely cultivated in the provinces along the Yangtze River. Shengli is the first widely cultivated oilseed rape cultivar in China introduced from Japan in the 1950s. ZS11, Zheyou7, Gangan and Shengli are SWORs. All eight accessions were planted in the experimental field at Huazhong Agricultural University in Wuhan or in a greenhouse under a photoperiod of 16 h of light and 8 h of dark at 22 °C and 70% relative humidity. A natural population including 210 rapeseed accessions was collected from the world major rapeseed-growing countries to represent the genetic diversity of rapeseed^1^. This natural population was planted in the experimental field in two SOR cultivation areas (Xining, Qinghai, 36° 35′ N, 101° 47′ E and Lanzhou, Gansu, 36° 02′ N, 103° 50′ E) in Northwest China in the 2013 and 2014 growing seasons and one SWOR cultivation area (Wuhan, 30° 36′ N, 104° 18′ E, China) in the 2014–2015 growing season. Flowering time was investigated and recorded as previously described^48,58^.

### Illumina sequencing, PacBio library construction, sequencing and optical genome maps construction

Illumina sequencing, PacBio sequencing and optical genome maps construction were performed at Novogene, China. High molecular weight DNA was extracted from 3-week-old seedlings. DNA fragments larger than 20 kb were selected by BluePippin electrophoresis (Sage Sciences). SMRTbell libraries were constructed as previously described^59^ and sequenced on the PacBio Sequel platform (Pacific Biosciences). Illumina paired-end sequencing libraries were generated following manufacturer’s standard protocol (Illumina) and sequenced on the Illumina HiSeq platform. Paired-end 150-bp reads were generated from libraries with an insert size of 350 bp. Illumina genomic reads of each accession were used as the input of the Jellyfish tool^60^ to obtain the *k*-mer frequency for estimating genome size. BioNano optical mapping was performed by digesting and labelling medium molecular weight DNA with the single-stranded nicking endonuclease Nt.BspQI according to BioNano’s standard protocol. The labelled DNA molecules were stretched and imaged with the BioNano Irys system (https://bionanogenomics.com/support-page/bionano-solve/).

### Hi-C library construction and sequencing

About 1.5 g of 3-week-old seedlings were used for Hi-C experiment. The experiment procedures were similar to a previous study^61^ but some steps were improved for efficiency. To digest extra protein and make the nuclei more permeable, the nuclei were resuspended in 150 μl of 0.5% SDS buffer and incubated at 62 °C for 5 min. Chromatin was digested for 12 h with 20 units of DpnII restriction enzyme (NEB) at 37 °C and the resuspended mixture was incubated at 62 °C for 20 min to inactivate the restriction digestion. The DNA pieces between 300 and 500 bp were excised and purified using Ampure XP beads (Beckman Coulter). The library was constructed by an Illumina TruSeq DNA Sample Prep Kit and sequenced by Illumina Hiseq Xten with 2 × 150-bp reads.

### Contig assembly and polishing

De novo genome assembly was performed mainly using the PacBio SMRT long reads. Subreads polishing and contigs assembly was primarily carried out using Falcon^62^ (falcon-2017.11.02–16.04-py2.7) with length_cutoff_pr = 6,000. We additionally configured pa_HPCdaligner_option = -v -B128 -t32 -e.75 -h480 -l3200 -w8 -T8, ovlp_HPCdaligner_option = -v -B128 -t32 -e.96 -l2500 -T8, falcon_sense_option = –output_multi–min_idt 0.70–min_cov 3–max_n_read 300, overlap_filtering_setting = –max_diff 110–max_cov 165–min_cov 3–bestn 10 with parameters optimized for eight *B. napus* genomes assembly. The subreads were assembled using Canu^63^ v.1.6 after Falcon polishing with correctedErrorRate = 0.05. We mapped PacBio sequencing reads to the draft contigs acquired by Canu and Falcon using pbalign and polished the resulting contigs using Quiver^64^ with arrow as algorithm. On this basis, contigs were polished using Illumina PE reads (insertion size = 350 bp) and pilon 1.18 (ref. ^65^). For the polished contigs, the unique sequences in Canu assembly while not being contained in Falcon assembly were merged to obtain final contigs.

### Pseudo-chromosome construction

Pseudo-chromosome was constructed with Hi-C data using the 3D-DNA pipeline^66^. The Hi-C reads were aligned to the polished contigs using the Juicer pipeline^67^. The 3D-DNA pipeline was run with the following parameters: -i 1 -r 5. The results were polished using the Juicebox Assembly Tools^68^. The Hi-C scaffolding resulted in 19 chromosome-length scaffolds. The scaffolds nomenclature was adopted for the chromosome numbering on the basis of their collinearity with 19 chromosomes of Darmor-*bzh* genome.

### Phylogenetic analysis

Protein sequences of *A. thaliana*, *B. oleracea*, *B. rapa* and eight assembled *B. napus* accessions were compared using BLASTP (e-value cut-off 1 × 10^−5^). Orthologous groups of sequences were constructed on the basis of the best bidirectional hits. A total of 1,235 groups with single member from each species were selected and the sequences of each organism were concatenated into one long protein sequence. Concatenated sequences were aligned using MAFFT^69^ and well-aligned regions were extracted using Gblocks^70^ with −t = p, −b4 = 5, −b5 = h. Trees were then constructed using multithreaded RAxML^71^, the PROTGAMMAWAG model and 100 bootstrap replicates. Tag SNPs^72^ were selected using PLINK^73^ (v.1.90) with parameter ‘–blocks’ to construct neighbour-joining tree. The neighbour-joining tree was constructed using TreeBeST (v.1.9.2, https://github.com/lh3/treebest) software with 1,000 replicates of bootstrap. An online tool Interactive tree of life (iTOL)^74^ v.3 was used to display the neighbour-joining tree. PCA of all SNPs were performed using genome-wide complex trait analysis (GCTA)^75^ v.1.91.7 software with default parameters.

### SNPs and InDels analysis of different accessions

The remaining seven genomes were aligned to reference genome ZS11 using Mummer^76^ (v.3.23) with parameters settings ‘-g 1000 -c 90 -l 40’. The alignment block was then filtered out of the mapping noise and the one-to-one alignment was identified by delta-filter with parameters settings ‘-r -q’. Show-snps was used to identify SNPs and InDels (<100 bp) with parameter setting ‘-ClrTH’. All clean reads were mapped to the ZS11 genome using BWA-MEM^77^ with the default parameters. Picard program was used to filter the PCR duplicates of reads and reads around InDels were realigned with the IndelRealigner option in the genome analysis toolkit (GATK)^78^. The unique mapping data were used for identifying SNPs using the GATK. Only variations detected with both tools were identified as high-quality SNPs. The distribution of SNPs in the genome was demonstrated by Circos^79^.

### Structural variation analysis of different accessions

To identify translocations and inversions, we aligned the other seven genomes to the ZS11 reference genome assembled in this study using Mummer. For the original alignment block to be filtered, we picked a unique alignment block that is longer than 1,000 bp. Aligned blocks that appeared as reverse matches as potential inversion regions were further manually checked and merged. At the same time, the alignment block between different chromosomes was considered to be an interchromosomal translocation. SyRI (https://github.com/schneebergerlab/syri) was used to identify translocation regions on the basis of the presence of non-collinear alignment blocks on both sides. We used the same method as Sun et al.^80^ to identify genes with large structure variations, which mapped gene sequence (extending the longest transcript of each gene 2 kb upstream and downstream) to query genomes using BWA-MEM.

### Identification of PAVs and pan-reference genome construction

The potential PAV sequences of seven genomes relative to reference genome ZS11 were identified using show-diff in Mummer (v.3.23). First, sequences that intersected with the gap region in the respective genome were excluded. On the other hand, sequence with feature type ‘BRK’ was filtered out, which was considered to be non-reference sequence which aligned to the gap-start or gap-end bounder. To identify the true respective unique sequences, the candidate PAV sequence was mapped to the ZS11 genome with parameter setting ‘-x asm10’ using minimap2 (ref. ^81^) and the sequence covering >80% was filtered out to obtain the final PAV region. The gene having >80% overlap with PAV region was considered to be a PAV-related gene. Further, we used BWA-MEM to align Illumina reads of ZS11 to seven genomes to rule out the effects of false positives and filtered out genes covering >50% of the genes to obtain the final PAV genes. We stepwise added the PAV sequence and PAV genes with the order ZS11, Gangan, Zheyou7, Shengli, Tapidor, Westar, No2127 and Darmor to the current genome to construct a pan-reference genome.

### Gene index in nine *B. napus*

MCscanx^82^ was used to identify collinear orthologues between query genomes and ZS11 genome, with at least five homologous genes and fewer than ten gaps required to call a collinear block. On the other hand, some translocations would also not need to meet the threshold for syntonic search. Therefore, for the genes that have established gene index pairs with ZS11 or with at least one species, we also added reciprocal best-hits as evidence to construct gene index pairs. We combined all collinear orthologues to construct the *B. napus* gene index with a set of unique ID (HUBna).

### SNP genotyping and SNP-GWAS of BN-NAM population

The 15 RIL families of BN-NAM population were genotyped by sequencing previously^41^. The genotypes of all 15 RIL families in the BN-NAM population were reanalysed according to the original method as previously described^41^ but using the newly assembled ZS11 genome sequence as the reference. A joint linkage map containing 122,899 SNPs was generated by integrating SNPs in the 15 RIL families. A set of 5,419,567 SNPs were identified for the whole-genome variant map according to the original method, as previously described^41^. GWAS was performed using a mixed linear model (MLM) in genome-wide efficient mixed model association (GEMMA) software^83^. MLM was coupled with estimated relatedness matrix as a random effect, which was estimated by GEMMA^83^. The effective number of independent markers (*N*) was calculated using the GEC tool^84^ and the suggestive *P* value (1/*N*) was set as the threshold^85^. To identify independent peak SNPs of association signals, SNPs passing the *P* value of GWAS thresholds were further clumped to remove the dependent makers caused by linkage disequilibrium (LD; *r*^2^ > 0.30) and interval (±500 kb) using the clumping function in Plink. *r*^2^ represents the degree of linkage disequilibrium.

### PAV genotyping and PAV-GWAS

To construct PAV haplotype maps of the 16 founder accessions of the NAM population, we combined PAVs across eight *B. napus* accessions identified by whole-genome comparative analysis using the ‘bayesTyperTools combine’ module in BayesTyper v.1.3.1 and the vcf file contained 88,781 AVs and 95,772 PVs as variant candidates in 19 pseudo-chromosomes. The WGS reads *k*-mers of 16 founder accessions of the NAM population were counted using KMC^86^ v.3.1.0 with parameters –k55 and –ci1. A read *k*-mer bloom filter was created from the KMC output using the ‘bayesTyperTools makeBloom’ module with default parameters. The ‘bayesTyper genotype’ module was used to estimate genotypes of each line based on *k*-mer counts from sequencing reads. To accurately estimate the noise parameters, the genotyping procedure included all the PAVs and 1 × 10^6^ random SNVs; all the unplaced contigs in the reference genome were treated as decoy. Then, the PAV haplotype maps of parent lines were mapped to 2,141 RILs based on high-density genetic linkage map as previously described in SNPs genotyping^41^. Finally, 27,216 unique polymorphic PAVs were obtained with MAF large than 0.05. PAV-GWAS was performed for silique length, seed weight and flowering time using MLM in GEMMA v.0.98 software.

### Population structure and genotype analysis of *FLC*

To obtain the genotypes of *Bna.A02FLC* and *Bna.A10FLC* in the natural population consisting of 210 rapeseed accessions^1^, we mapped the resequencing data to the genomes of ZS11, Westar and Tapidor, representing the SWOR, SOR and WOR ecotypes, respectively, using BWA-MEM. SAMtools^87^ was used to filter uniquely mapped reads with parameter setting ‘-q 30’. Normalized coverage was defined as the ratio of the insertion site coverage and the average coverage of 100 kb of surrounding genes. Searching for unique fragments on both sides of insert fragment, 30 candidate loci were selected among three genomes. The R PCA function was used to perform PCA to screen out the representative points that contributed to the interpretation of the data variation. Each PAV in *BnaA10.FLC* and *BnaA02.FLC* was genotyped on the basis of the coverage of 15 and five representative sites, respectively. Normalized coverage higher than 0.25 was considered to cover the insertion fragment.

### Reporting Summary

Further information on research design is available in the Nature Research Reporting Summary linked to this article.

## Supplementary information

Supplementary Information Supplementary Figs. 1–41 and Supplementary Notes.

Reporting Summary

Supplementary Tables Supplementary Tables 1–53.

## Data Availability

All the raw sequencing data generated during the current study are available in the NCBI BioProject under accession number PRJNA546246. The genome assemblies and annotation files are available at the website http://cbi.hzau.edu.cn/bnapus. All the materials in this study, including introgression lines, are available upon request.
