# Supplementary material for: Eight high-quality genomes reveal pan-genome architecture and ecotype differentiation of Brassica napus
Source: Nat Plants. 2020 Jan 13;6(1):34–45. doi: 10.1038/s41477-019-0577-7 (PMC6965005; doi:10.1038/s41477-019-0577-7)
Supplement: Supplementary file 1 — Supplementary Figs. 1–41 and Supplementary Notes. [file 41477_2019_577_MOESM1_ESM.pdf]

nature plants

SUPPLEMENTARY INFORMATION

https://doi.org/10.1038/s41477-019-0577-7

In the format provided by the authors and unedited.

Eight high-quality genomes reveal pan-genome architecture and ecotype differentiation of *Brassica napus*

Jia-Ming Song[1,2,3], Zhilin Guan[1,3], Jianlin Hu[1,3], Chaocheng Guo[1], Zhiquan Yang[1,2], Shuo Wang[1,2], Dongxu Liu[1,2], Bo Wang[1], Shaoping Lu[1], Run Zhou[1,2], Wen-Zhao Xie[1,2], Yuanfang Cheng[1], Yuting Zhang[1], Kede Liu[1*], Qing-Yong Yang[1,2*], Ling-Ling Chen[1,2*] and Liang Guo[1*]

[1]National Key Laboratory of Crop Genetic Improvement, Huazhong Agricultural University, Wuhan, People's Republic of China. [2]Hubei Key Laboratory of Agricultural Bioinformatics, College of Informatics, Huazhong Agricultural University, Wuhan, People's Republic of China. [3]These authors contributed equally: Jia-Ming Song, Zhilin Guan, Jianlin Hu.
*e-mail: kdliu@mail.hzau.edu.cn; yqy@mail.hzau.edu.cn; llchen@mail.hzau.edu.cn; guoliang@mail.hzau.edu.cn

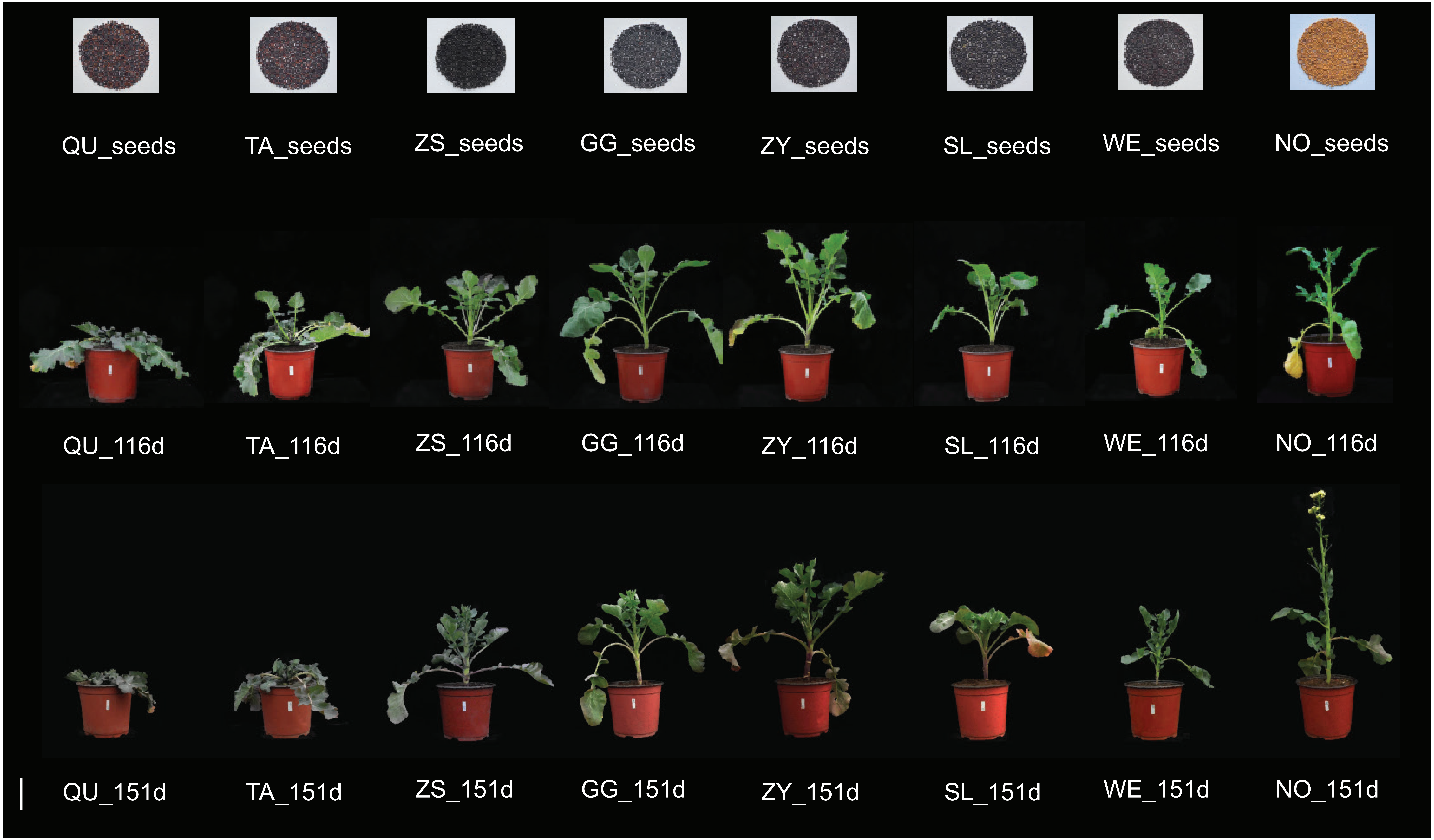

Supplementary Figure 1. The seeds and plants of eight *B. napus* accessions in 2018-2019 growing season in Wuhan. Plant pictures were taken at 116 days and 151 days after sowing. These plants were transplanted from the field to the pot at 106 days after sowing. Due to transplant, the growth and flowering were delayed compared to those grown in the field. Bar: 10 cm.

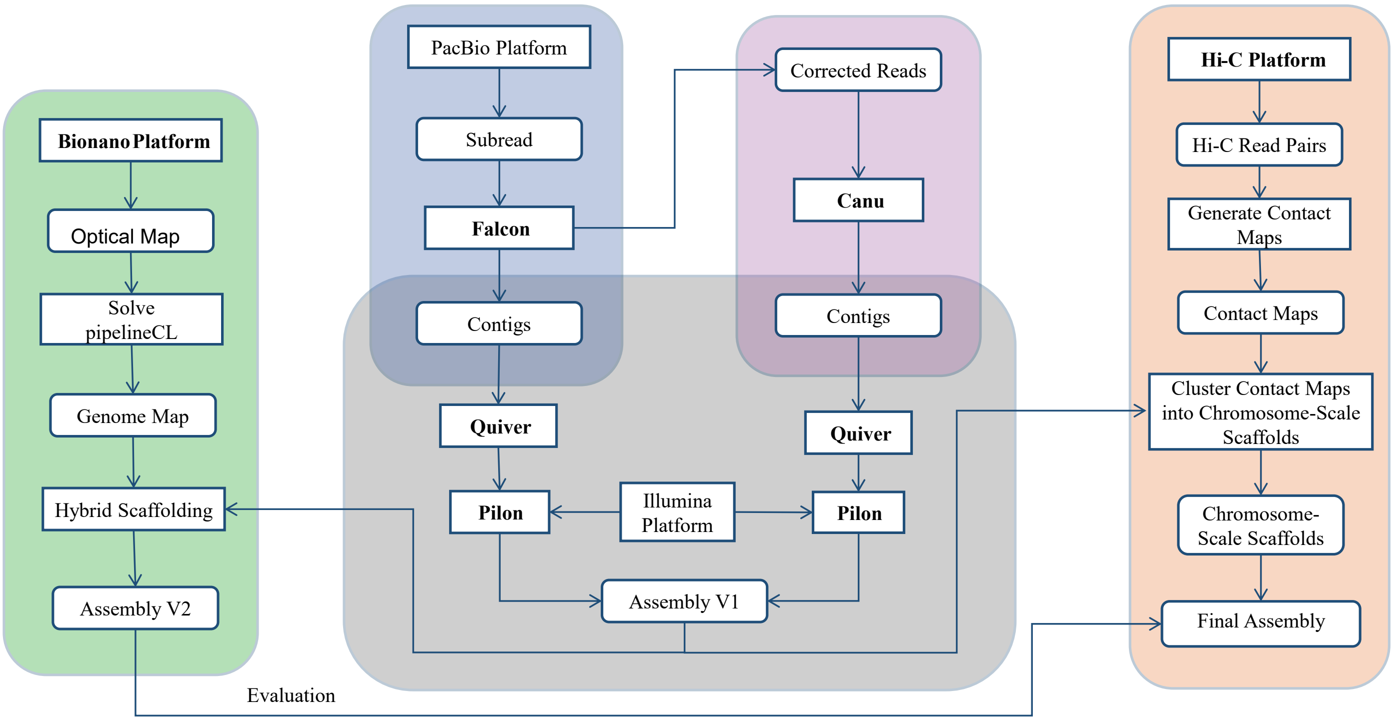

Supplementary Figure 2. The pipeline for genome assembly in this study. 3D-DNA was used to correct, order, orientate and cluster the contigs. Bionano hybrid scaffolds were used to evaluate the final assembly (see Method).

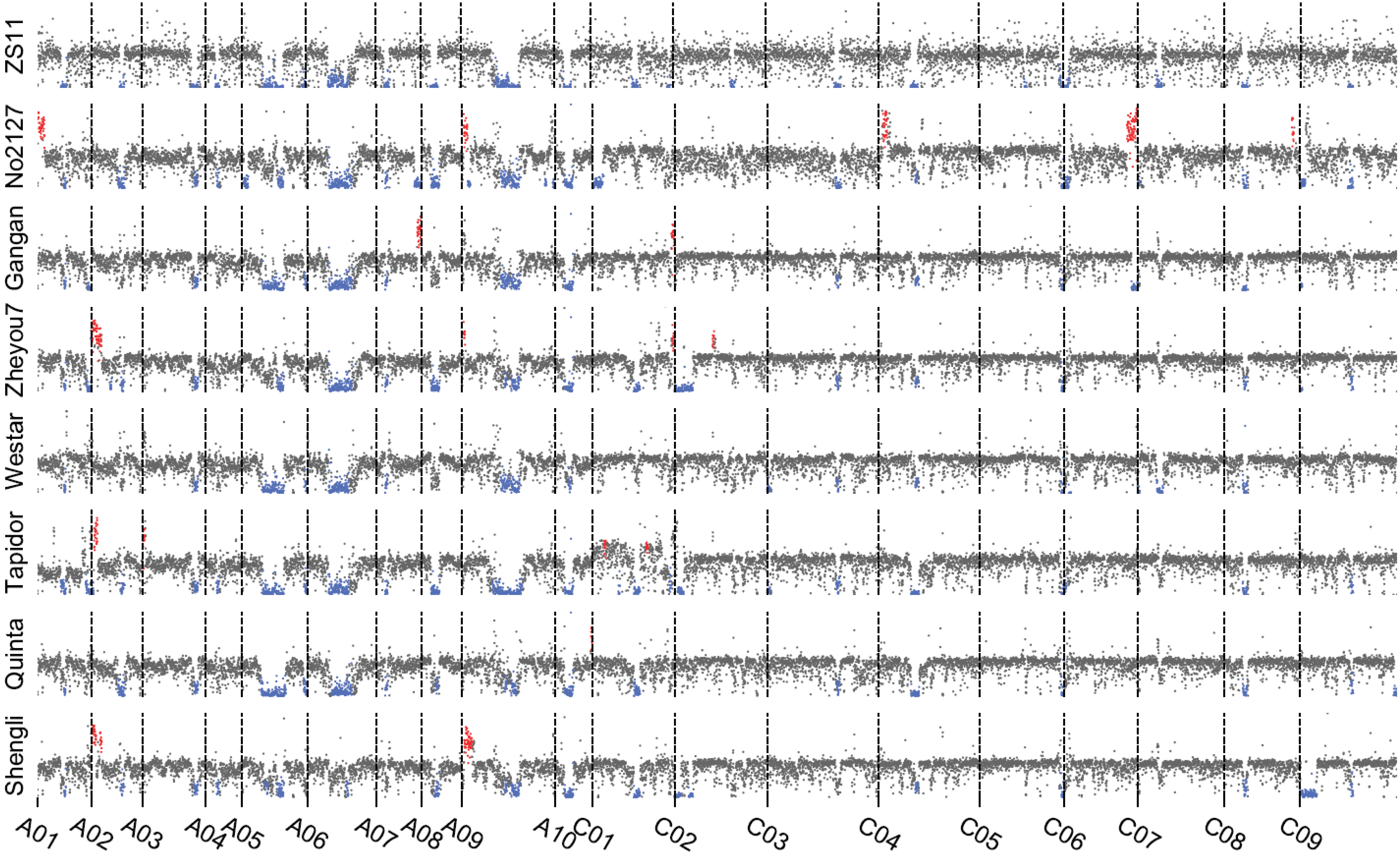

Supplementary Figure 3. Large segmental HEs of eight assembled accessions. Coverage depth obtained along genome after mapping Illumina sequence reads from eight assembled accessions to the 'ZS11' genome. The x-axis represents the 100kb regions of chromosomes A01-C09 in genome and the y-axis represents the coverage of these regions. Segmental HEs are revealed based on sequence read coverage analysis, where a duplication (red) is revealed by significantly greater coverage for a given segment than the rest of the genome (grey) and a deletion (blue) by little or no coverage for the corresponding homeologous segment.

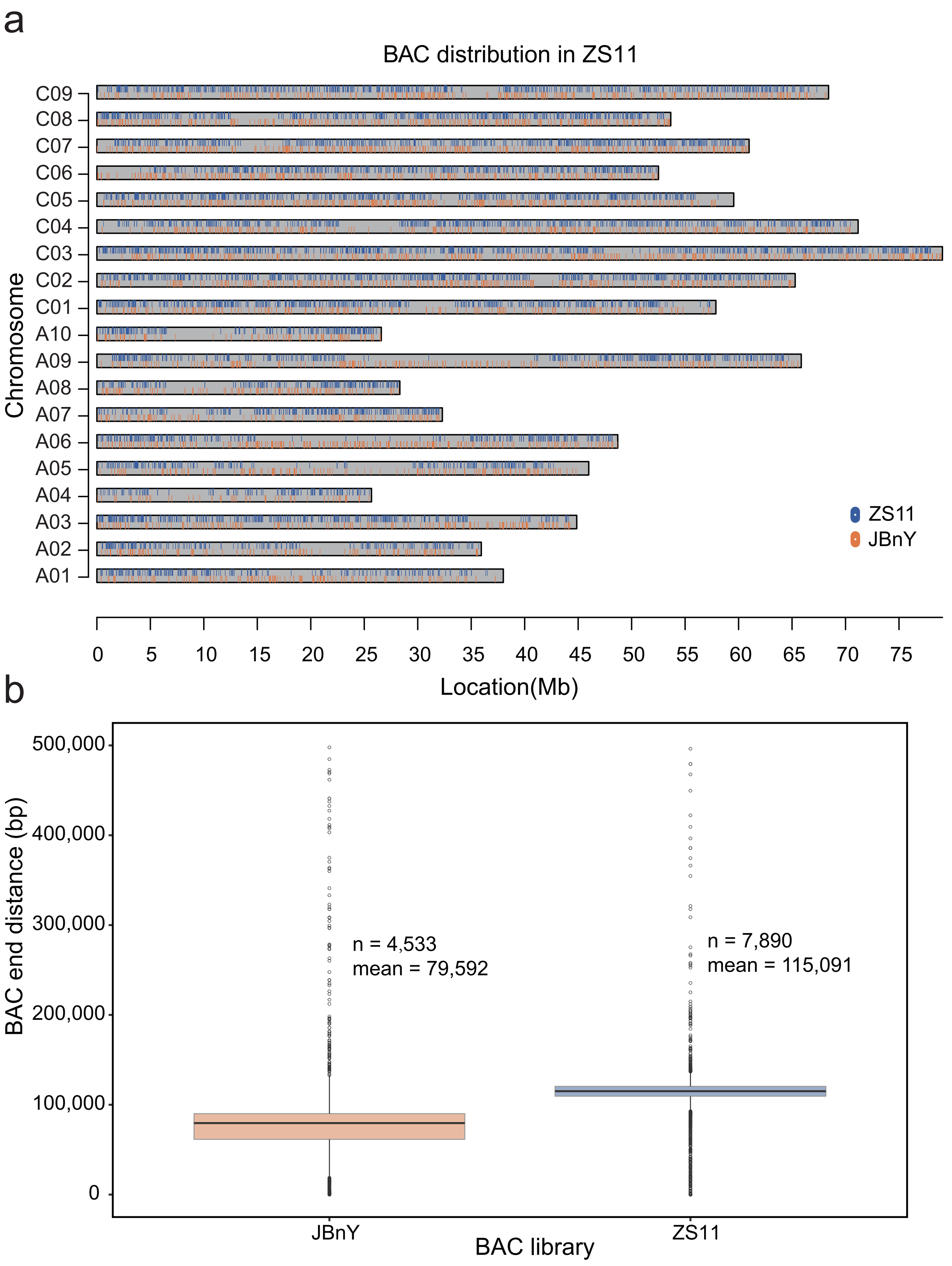

Supplementary Figure 4. Mapping results of paired BAC-end data on ZS11 assembly genome. **a,** The distribution of ZS11 and JBnY BAC mapped to genome. **b,** The distance of BAC pairs end. Additional details about the statistics can be found in Supplementary Notes (for all Supplementary Figures).

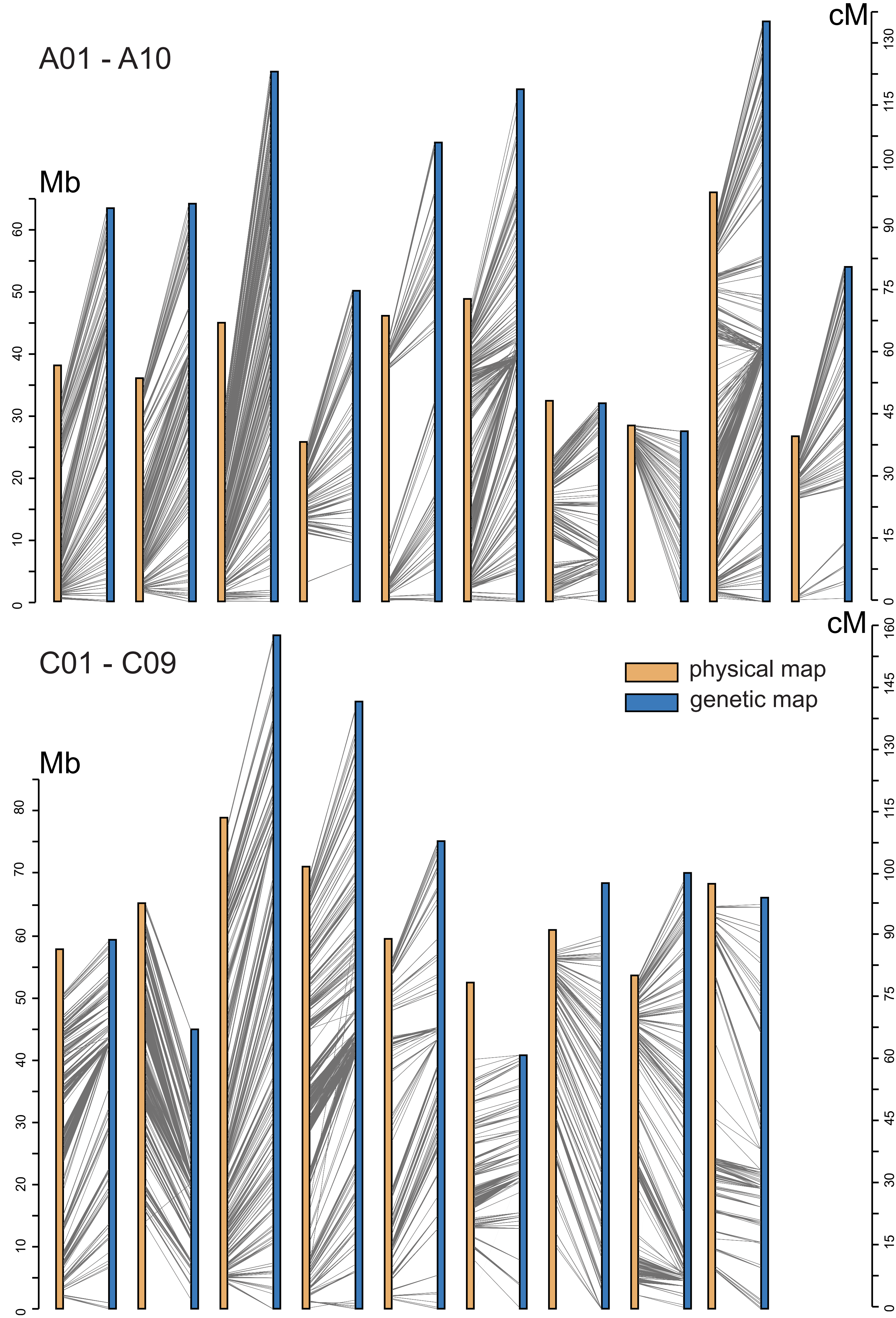

Supplementary Figure 5. Colinearity between genetic map and physical map. The genetic map was generated with a RIL population (ZS11 x Quantum), which including 7,158 SNP makers. A subset of 7,103 markers were uniquely mapped to the ZS11 physical map and constructed physical map.

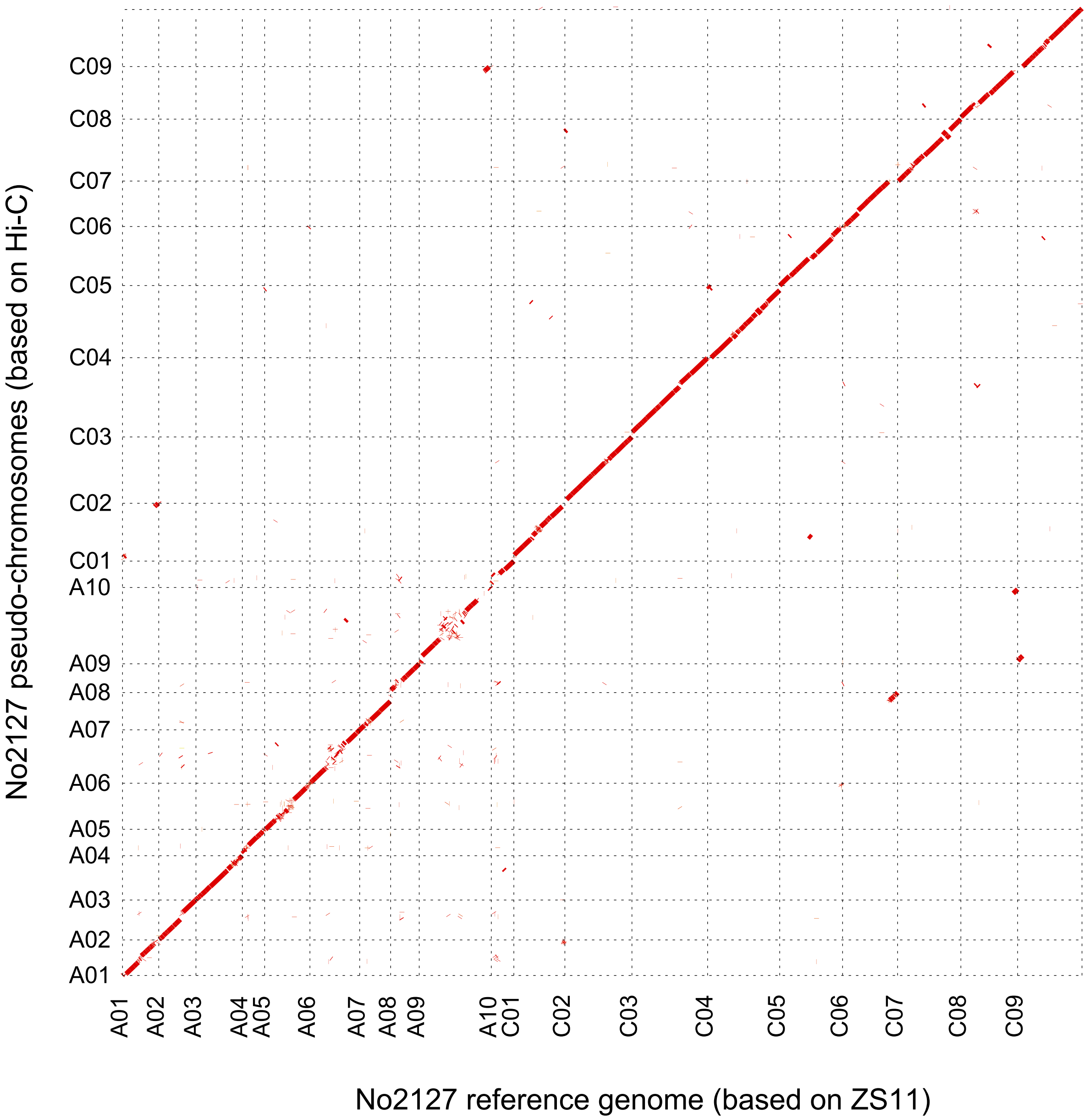

Supplementary Figure 6. The illustration of dot plots showing synteny regions between No2127 pseudo-chromosomes generated by Hi-C data and the pseudo-chromosomes of No2127 based on ZS11 genome. Horizontal axis shows the pseudo-chromosomes of No2127 based on ZS11 genome and the vertical axis shows No2127 pseudo-chromosomes (based on Hi-C) coordinate. Synteny regions were identified by Mummer (-c 90 -l 40).

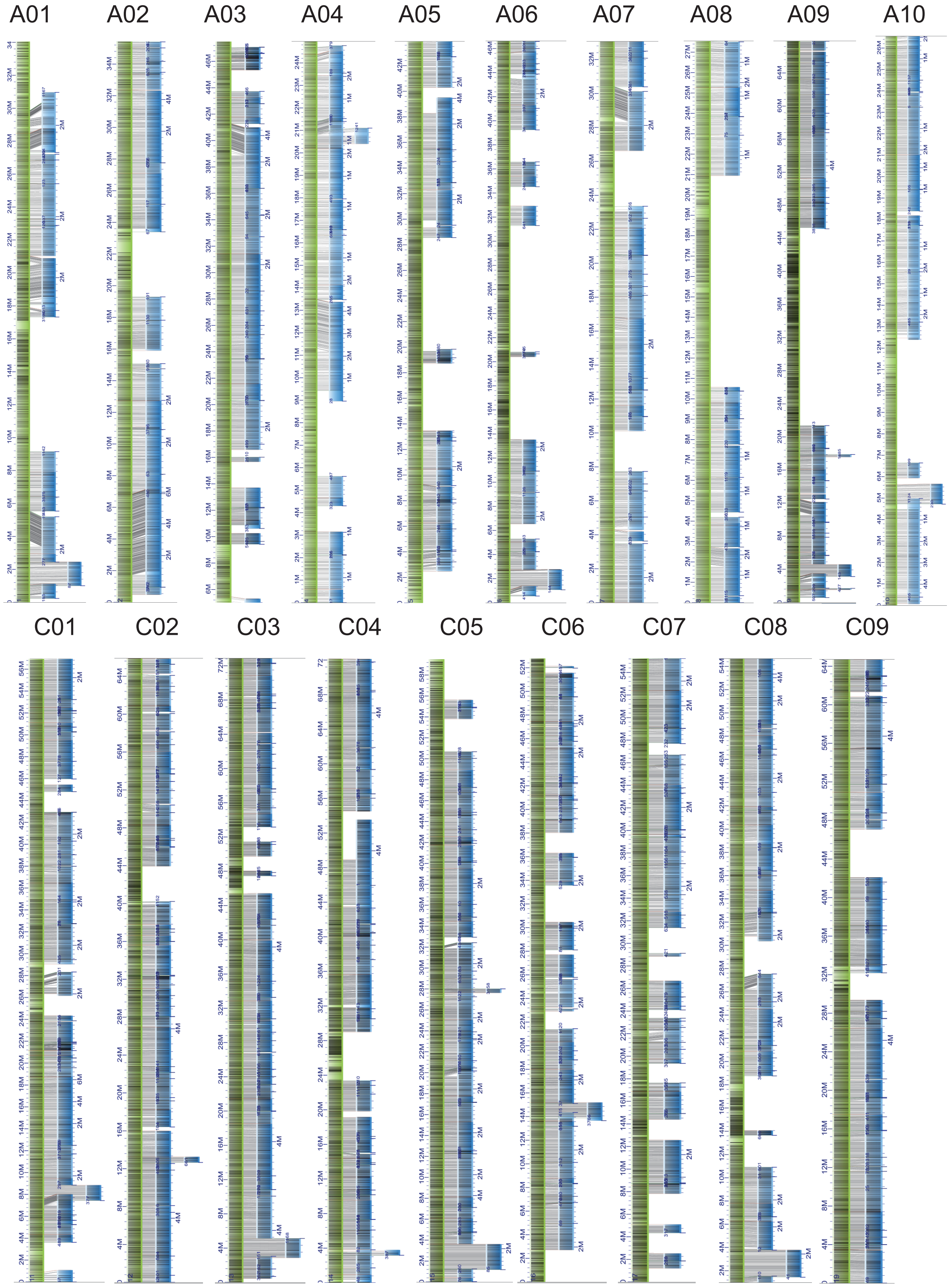

Supplementary Figure 7. Bionano alignment images of Westar assembly. Green represents the Westar assembly results, blue represents the assembly scaffolds by optical mapping, and gray lines represent the collinear regions. The alignment was performed with the hybridscaffold module in Solve (version 3.1).

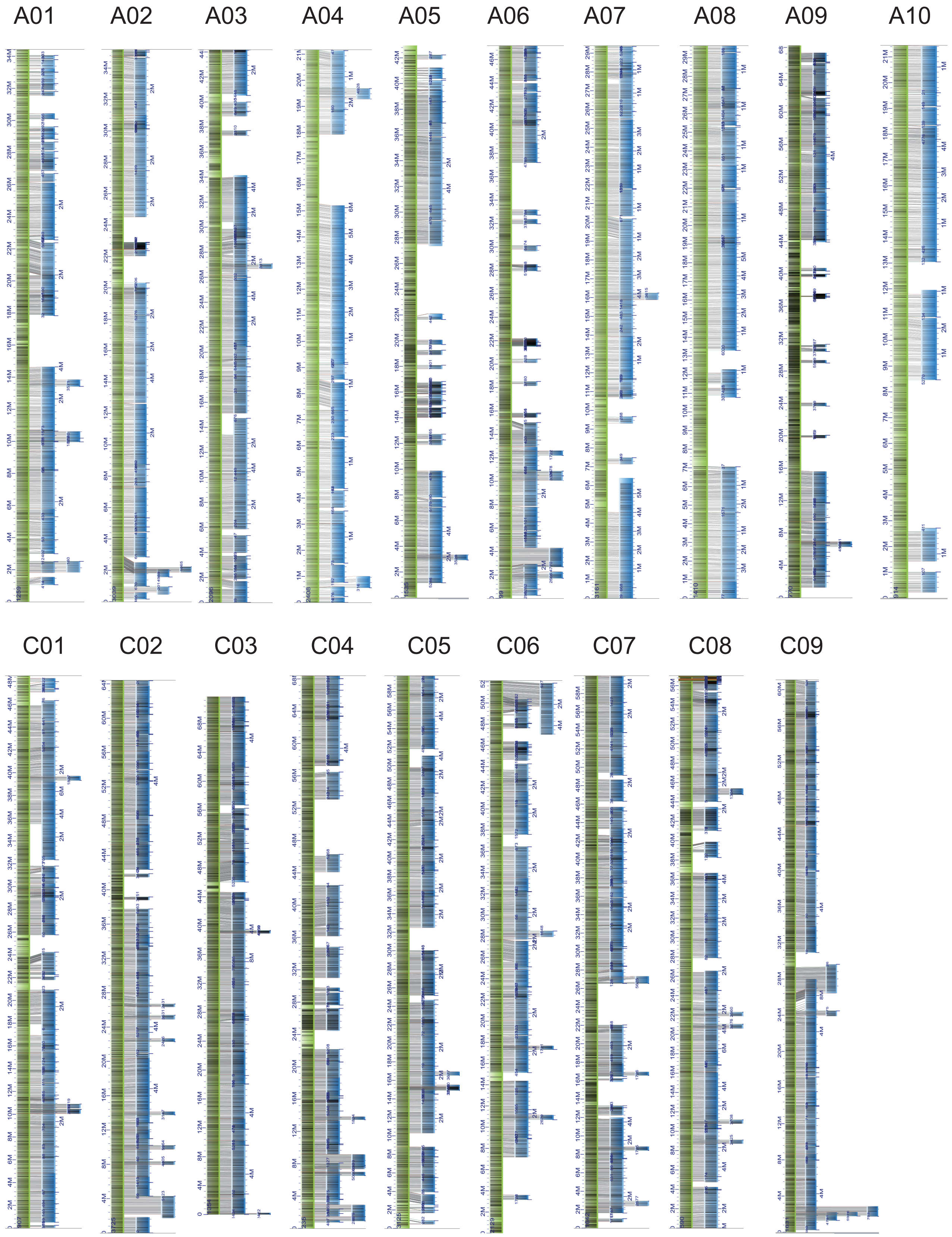

Supplementary Figure 8. Bionano alignment images of No2127 assembly. Green represents the No2127 assembly results, blue represents the assembly scaffolds by optical mapping, and gray lines represent the collinear regions. The alignment was performed with the hybridscaffold module in Solve (version 3.1).

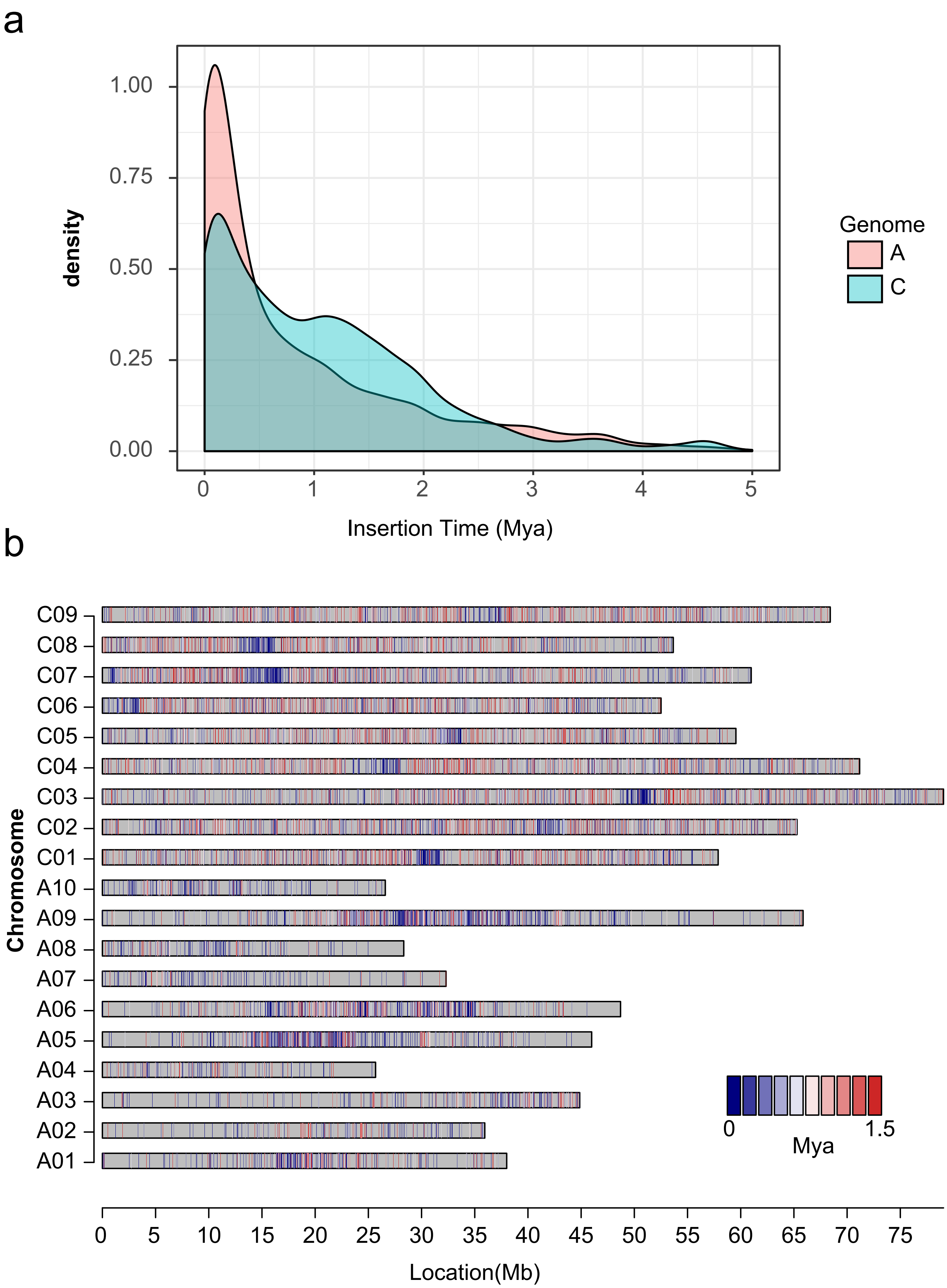

Supplementary Figure 9. The insertion time of high-confidence intact LTR retrotransposons in ZS11 genome. a, The frequency distribution of all intact LTRs insertion time. The C genome has an LTR insertion outbreak in 1-2 million years. **b,** The distribution of identified intact LTR on the genome. Blue to red represents insertion time from near to far. There are more distantly inserted LTRs on the C genome and the distribution is more uniform. Most of the LTRs in the A subgenome are recently inserted and tend to be clustered.

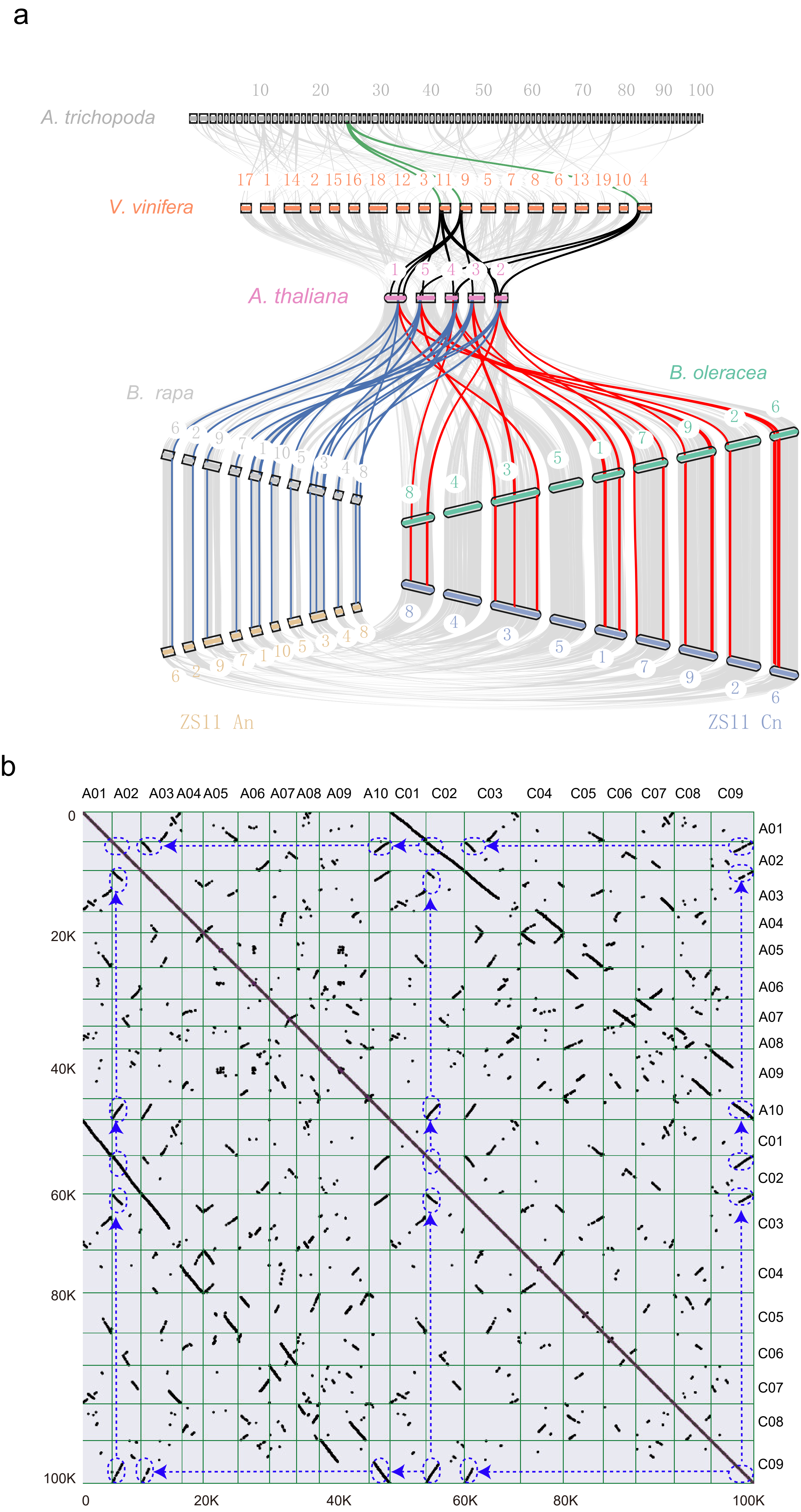

Supplementary Figure 10. The evolutionary history of the *B. napus* genome. a, Evolutionary scenario and genome duplication of *B.napus*. a. Grape has experienced WGT relative to the ancient genome (*A. trichopoda*). Subsequently, *A. thaliana* experienced two WGDs. *Brassica* (*B. napus*) experienced WGT again. Finally, the rapeseed genome was formed by allopolyploidy. **b,** Paralog retention (1-6 chromosomal relationships in blue circles) illustrates *Brassica* WGT and allopolyploidy events.

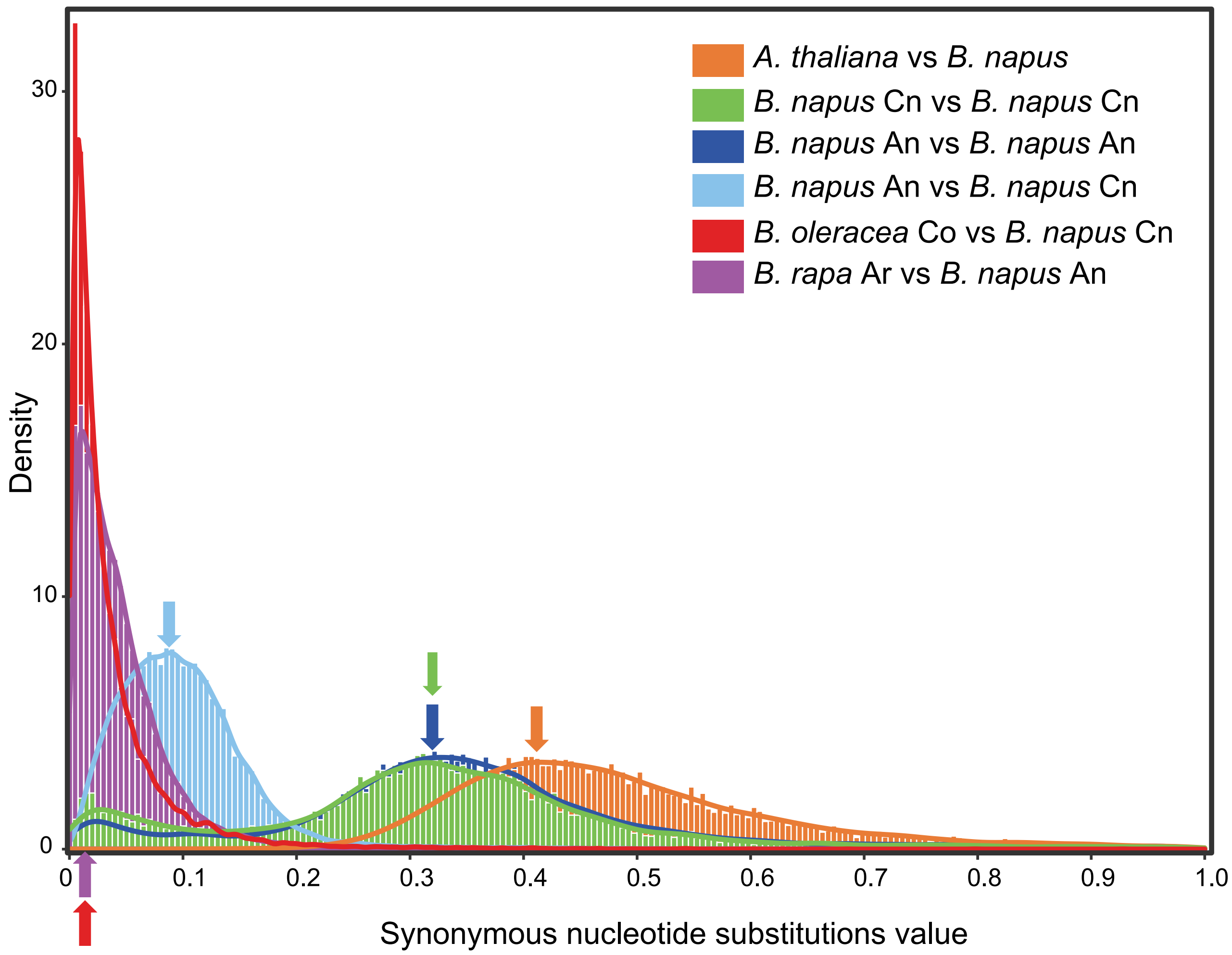

Supplementary Figure 11. Frequency distributions of synonymous substitution rates (Ks) between collinear genes in syntenic blocks. The K_S values between different genomes or sub-genomes are represented by columns of different colors. Arrows are marked at the peak of K_S, and arrows from left to right represent the differentiation time of *B. napus* and *B. rapa*/*B. oleracea* (allotetraploidy) ($K_S = 2.8*10^{-4}$ - $4.2*10^{-4}$, about 10,000 years ago); the differentiation time of *B. rapa* and *B. oleracea* ($K_S = 0.089$, about 3 million years ago); *Brassica* tripled time ($K_S = 0.328$, about 11 million years ago); the differentiation time of *A. thaliana* vs *B. napus* ($K_S = 0.417$, about 14 million years ago). The synonymous replacement rate r is chosen to be $1.5*10^{-8}$ mutations site/year.

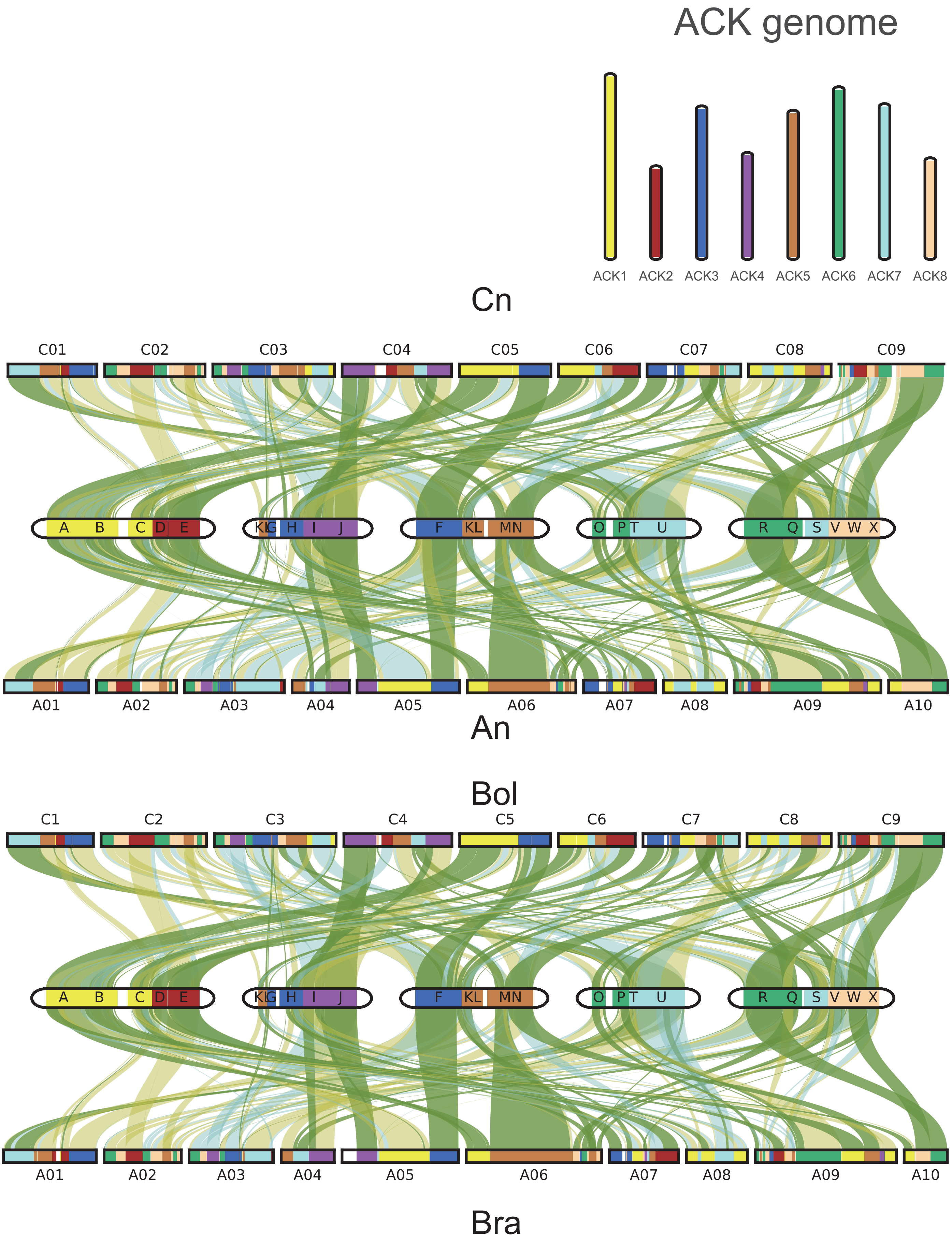

Supplementary Figure 12. Segmental colinearity among the genomes of *B. oleracea*, *B. rapa* , *B. napus* and *A. thaliana*. Syntenic blocks are defined and labelled from A to X (coloured) previously reported in *A. thaliana*. Subgenomic (LF, MF1 and MF2) and genes in each of blocks (see Supplementary Table 20).

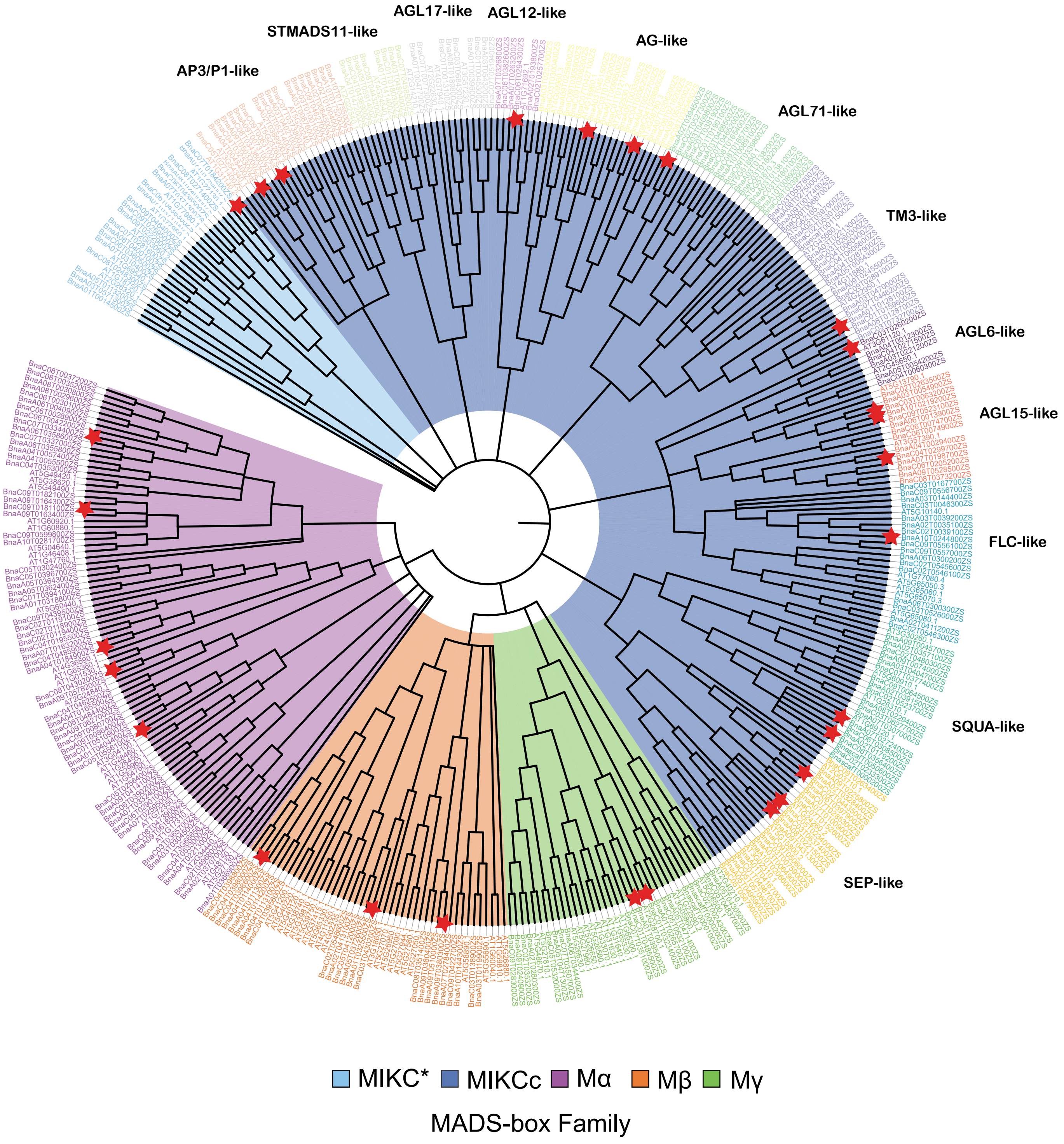

Supplementary Figure 13. Phylogenetic tree of MADS-box proteins in *B.napus* .
Phylogenetic analysis of 405 MADS-box proteins from *A. thaliana* (108) and *B. napus* (297) showed similar groupings between *B. napus* and *A. thaliana*. Referring to the *A. thaliana* classification, the colors of inner circle represent different subfamily. Further, the MIKCc subfamily is futher divided into 12 subgroups.

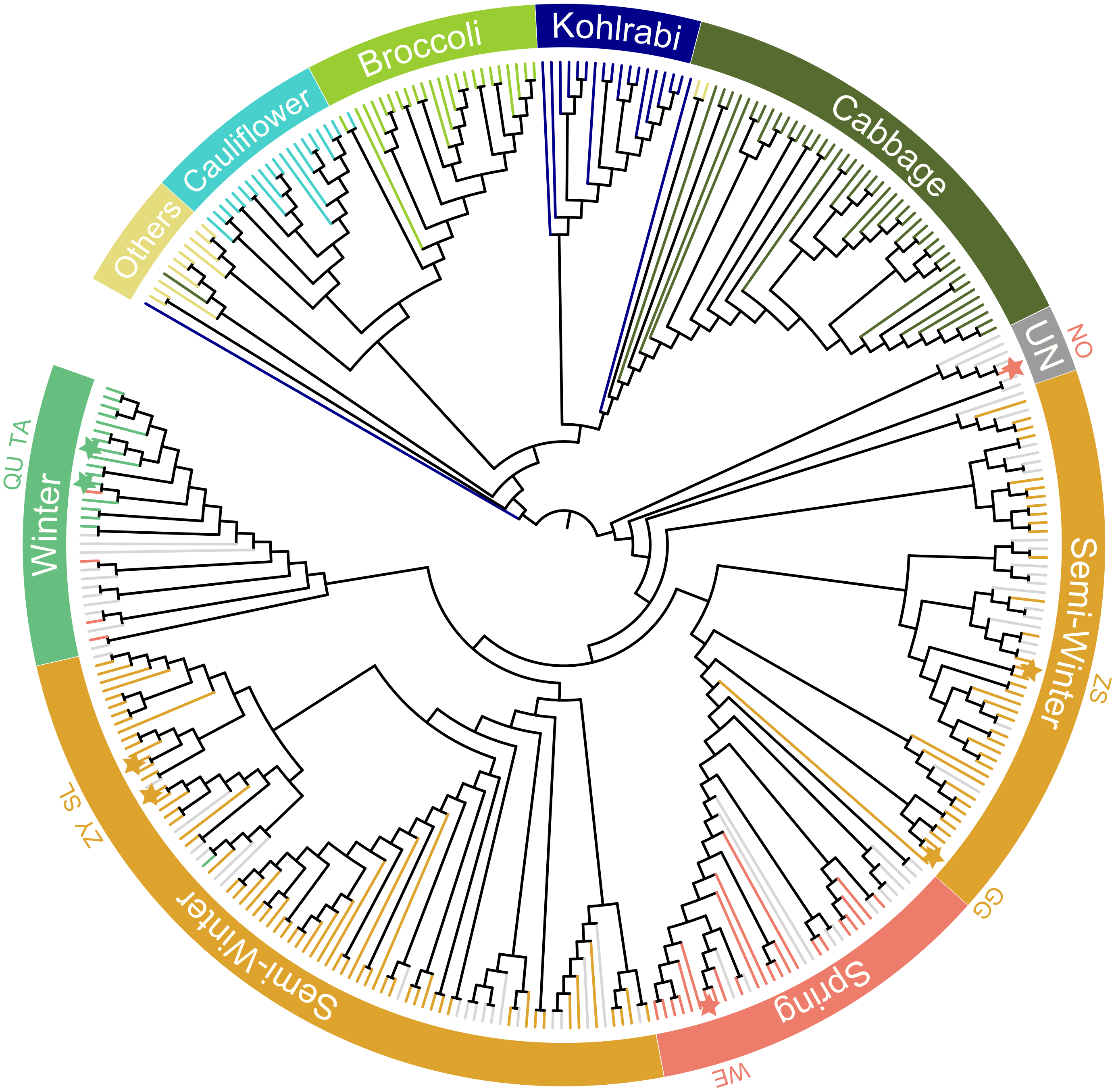

Supplementary Figure 14. A neighbor-joining tree of all 216 *B. napus* accessions and 119 *B. oleracea* accessions inferred from C subgenome SNPs. The eight reference accessions are represented by pentagrams. The outer ring indicates the group name of each clade.

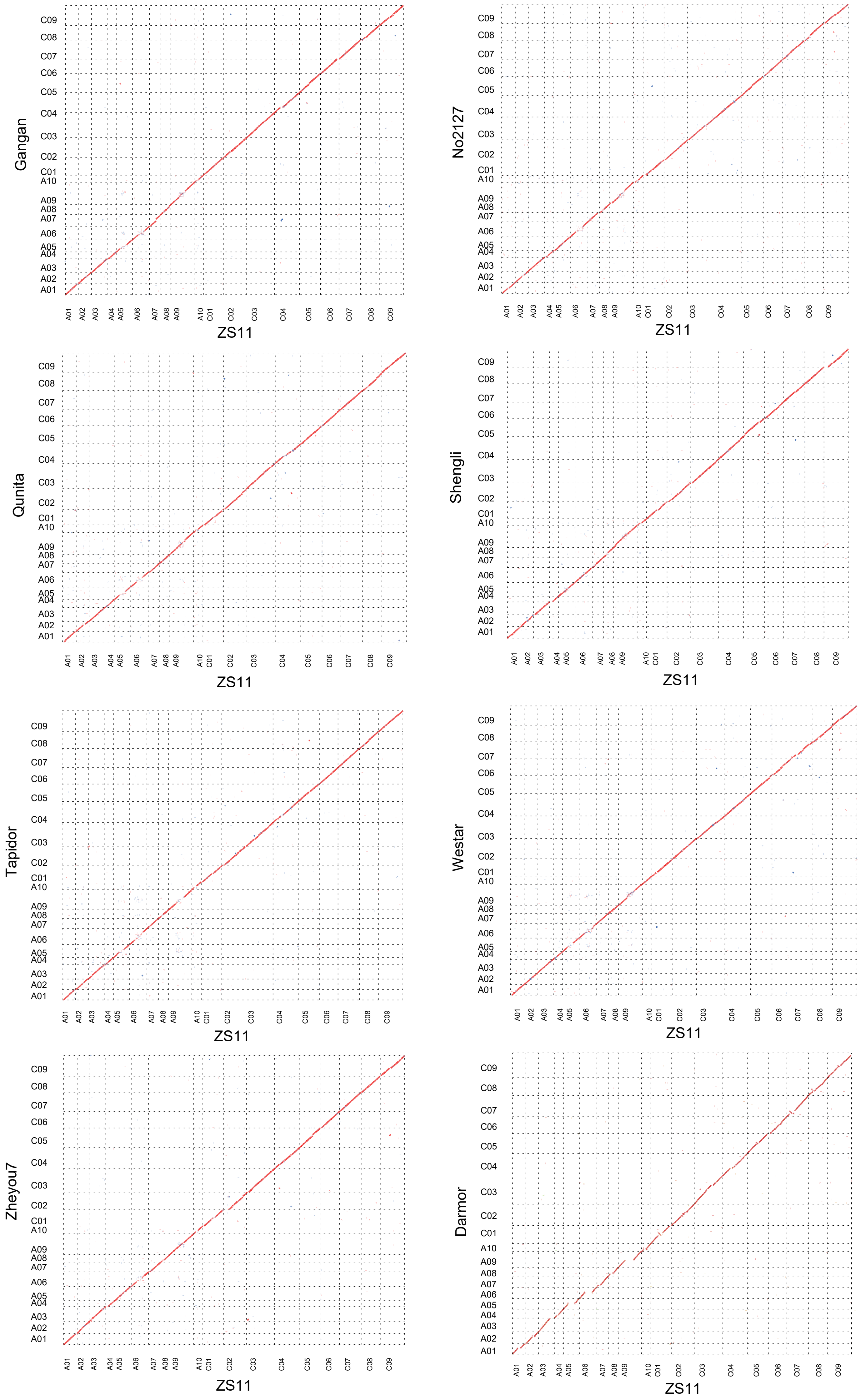

Supplementary Figure 15. The illustration of dot plots showing synteny regions between each of the other 8 *B. napus* genomes and ZS11 genomes. Horizontal axis shows reference coordinate and the vertical axis shows query coordinate. Red dots represent collinear regions above 1 kb, blue dots represent reverse alignments.

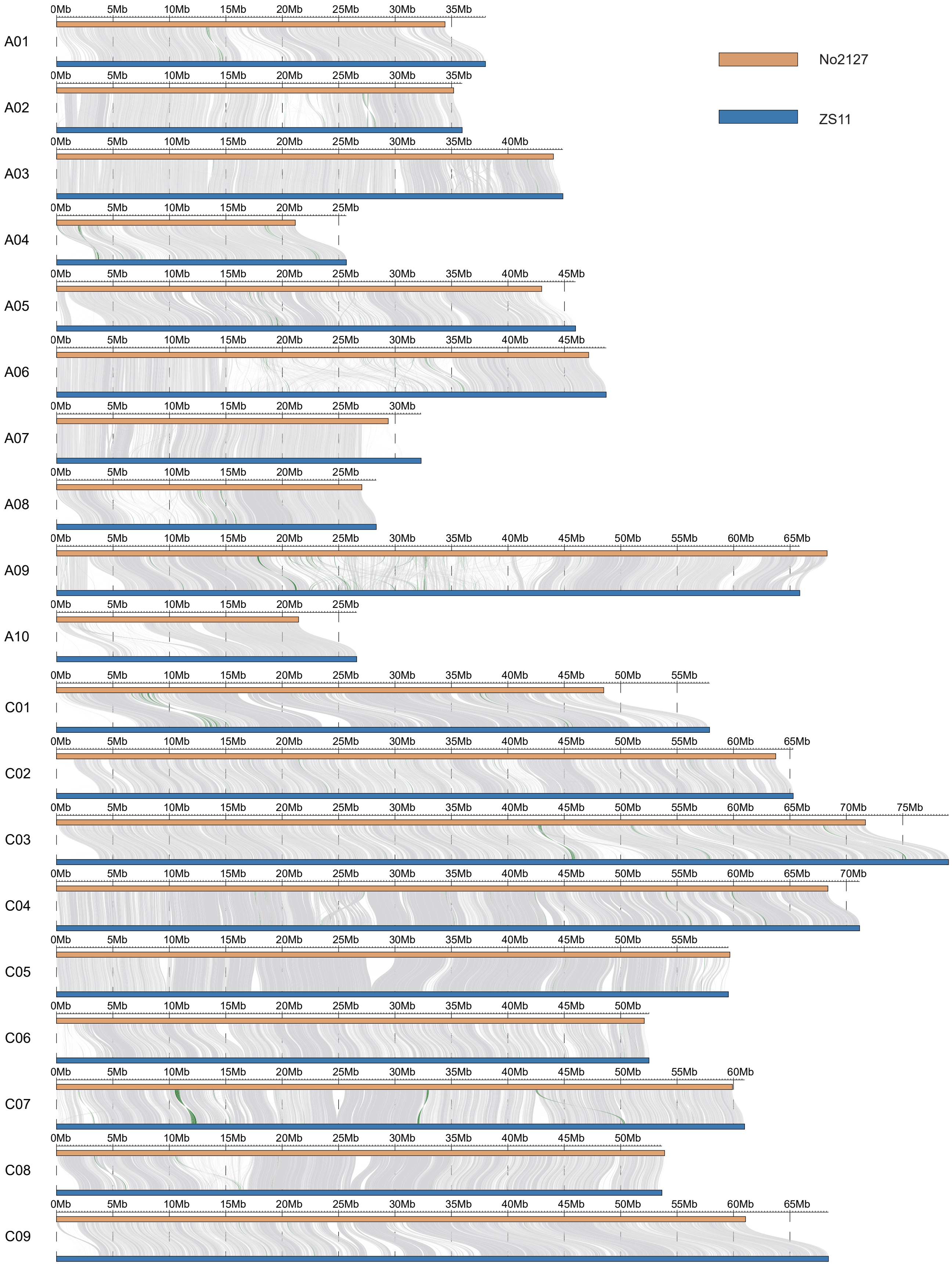

Supplementary Figure 16. Whole-genome comparison between ZS11 and No2127. Gray lines represent synteny blocks larger than 1 kb. Green lines represent reverse alignment. Total syntenic region between ZS11 and No2127 was 759.6 Mb. A ~430 kb inversion was observed near 10.5 Mb in C07. At C05: 27.4-29.9 Mb, No2127 has a ~3 Mb unique sequence.

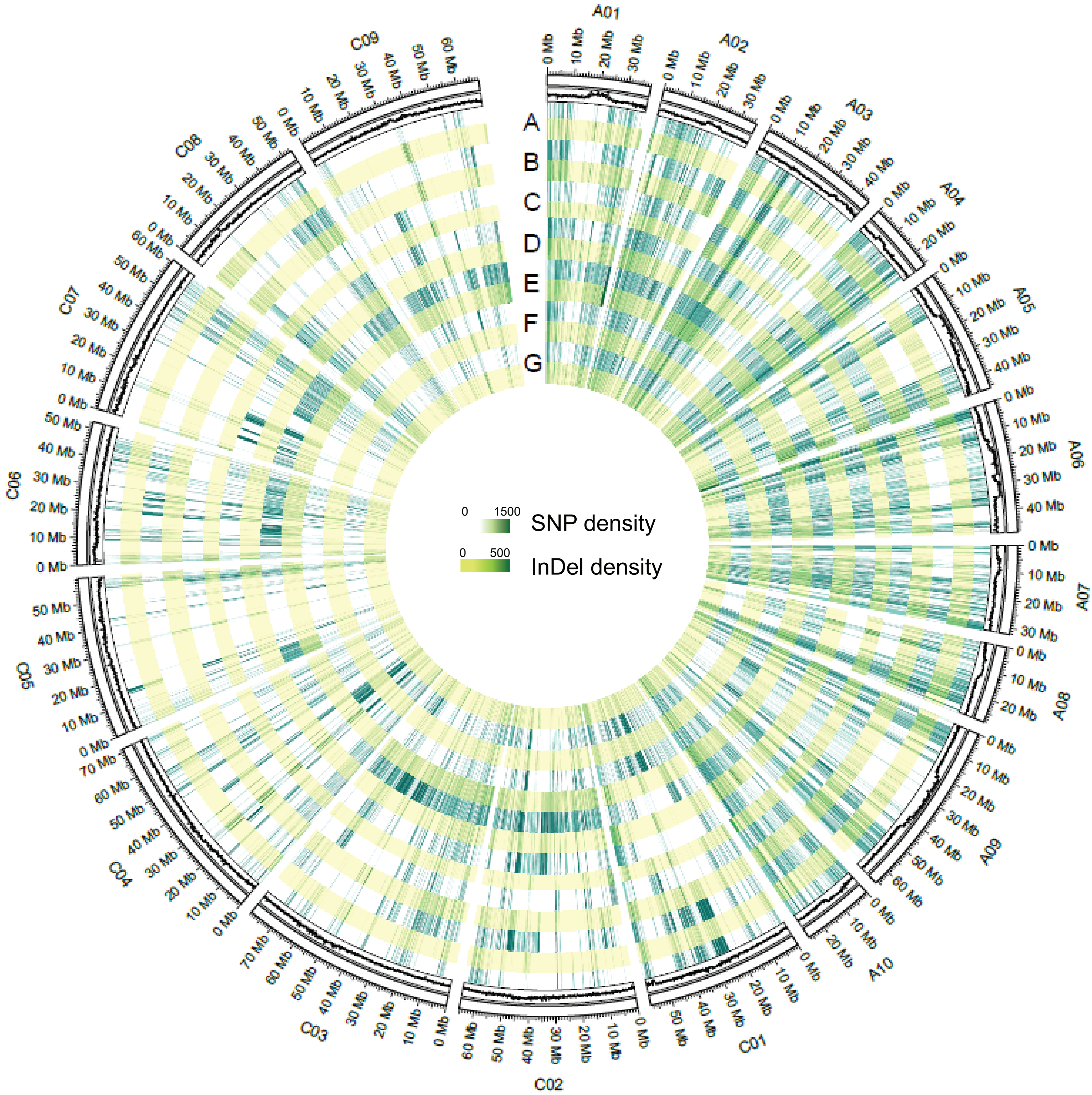

Supplementary Figure 17. The density of SNPs and InDels in seven *B. napus* genomes compared with ZS11 genome. A: Zheyou, B: Shengli, C: Gangan, D: Westar, E: No2127, F: Tapidor, G: Quinta. The correlation between the density of SNPs and InDels (R = 0.92) was caculated by R package 'cor.test'.

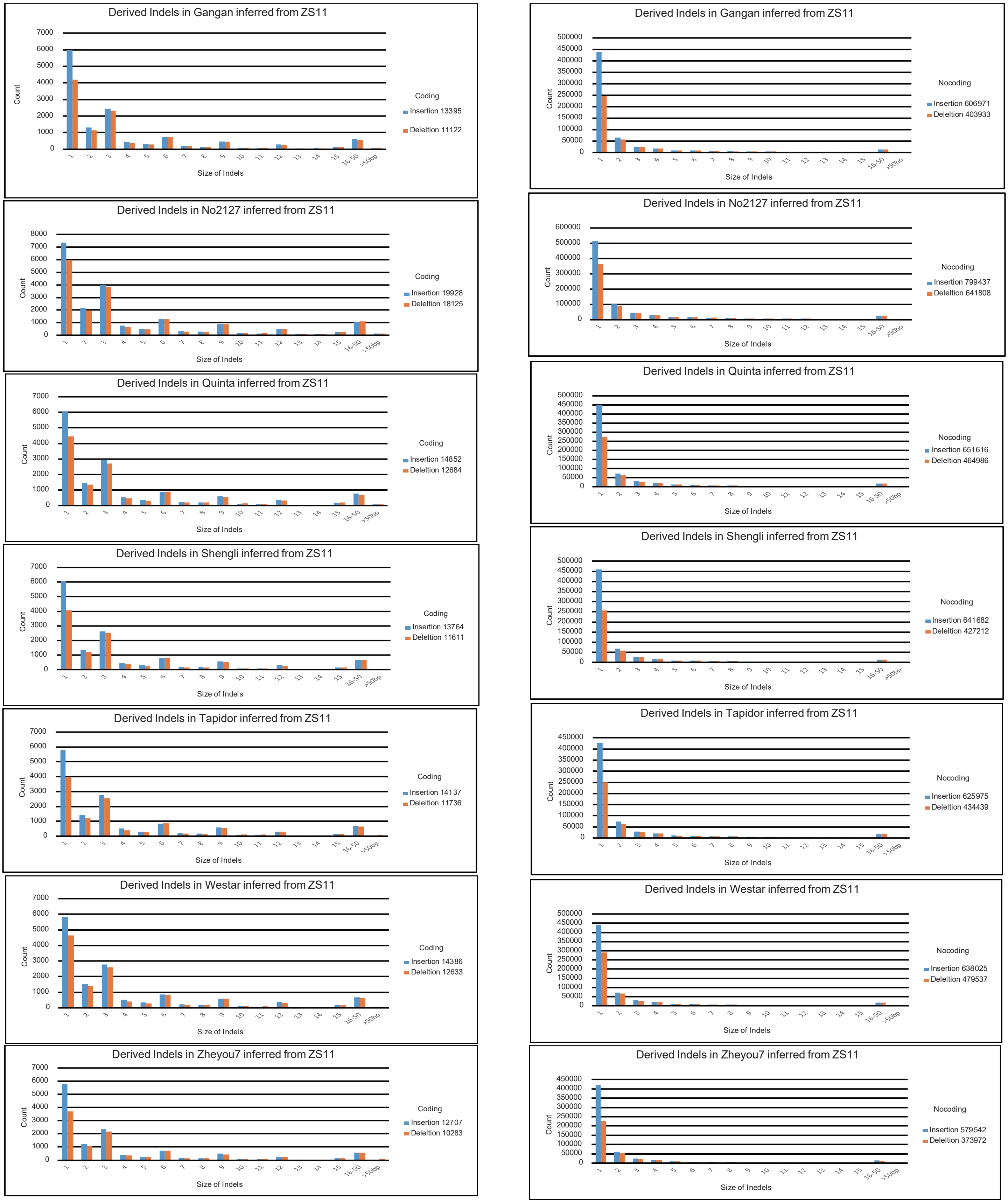

Supplementary Figure 18. The size distribution of insertions and deletions. Left panels show indels within or overlapping with coding regions, in which multiples of three bases are overrepresented. Right panels show indels in noncoding regions, with a declining trend as indel size increasing.

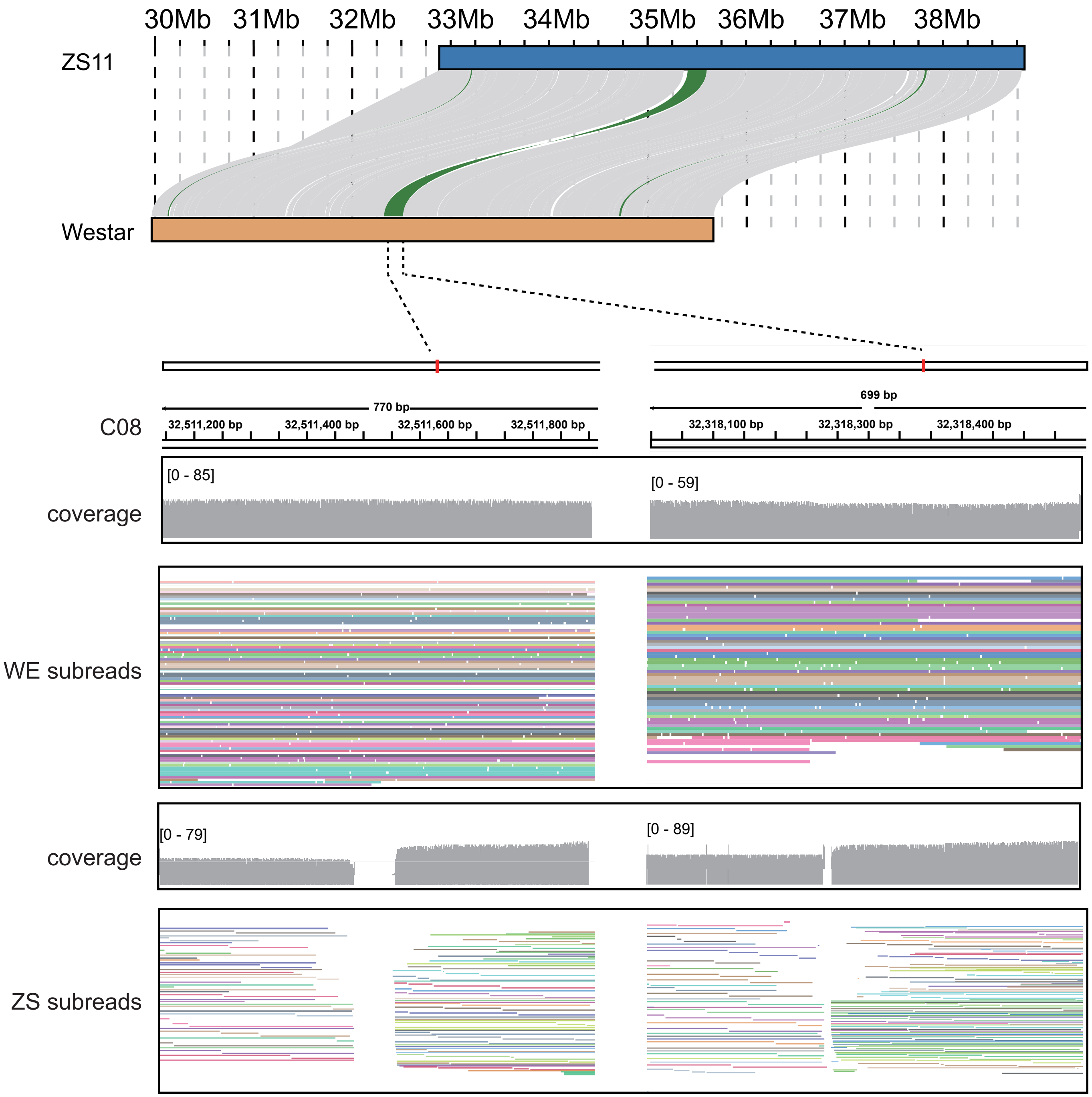

Supplementary Figure 19. Illustration of a 193 kb inversion in Westar C08 chromosome. The subreads of ZS11 and Westar were mapped back to reference genome Westar, and significant breakpoints were observed in the ZS11 reads at the boundaries of identified inversion. The subreads in different colors in the figure represent those from different zero-mode waveguides (ZMWs).

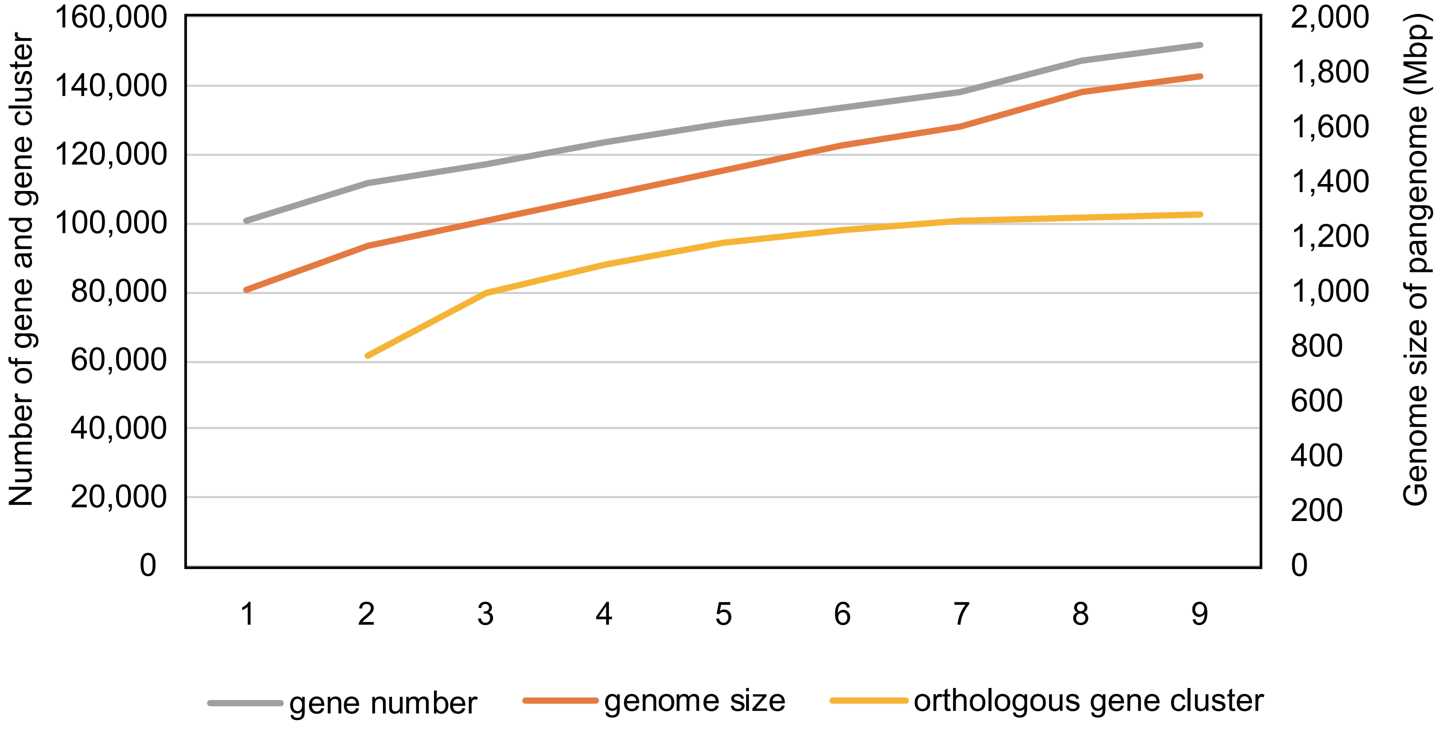

Supplementary Figure 20. Evaluation of the number of protein coding genes in the pan genome. Stepwise addition of *B. napus* accessions from n = 1 to n = 9 was performed to evaluate the number of coding genes in the pan genome. Orthologous gene clusters are defined as coding genes present in at least two *B. napus* accessions.

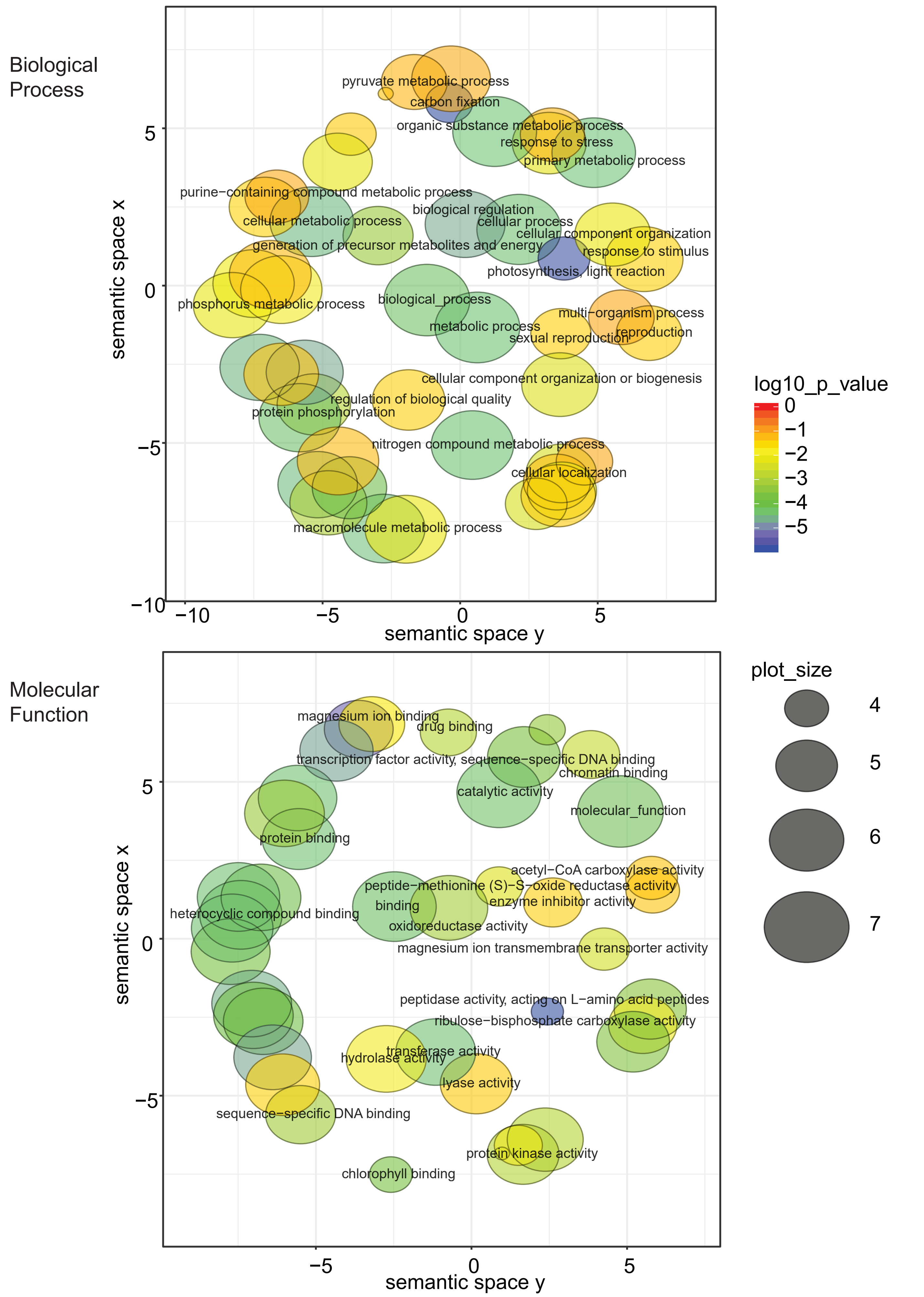

Supplementary Figure 21. The clustering of enriched GO terms of specific genes in Gangan. The scatterplot shows the cluster representatives (i.e. terms remaining after the redundancy reduction) in a two dimensional space derived by applying multidimensional scaling to a matrix of the GO terms semantic similarities. Plot_size indicates the frequency of the GO term.

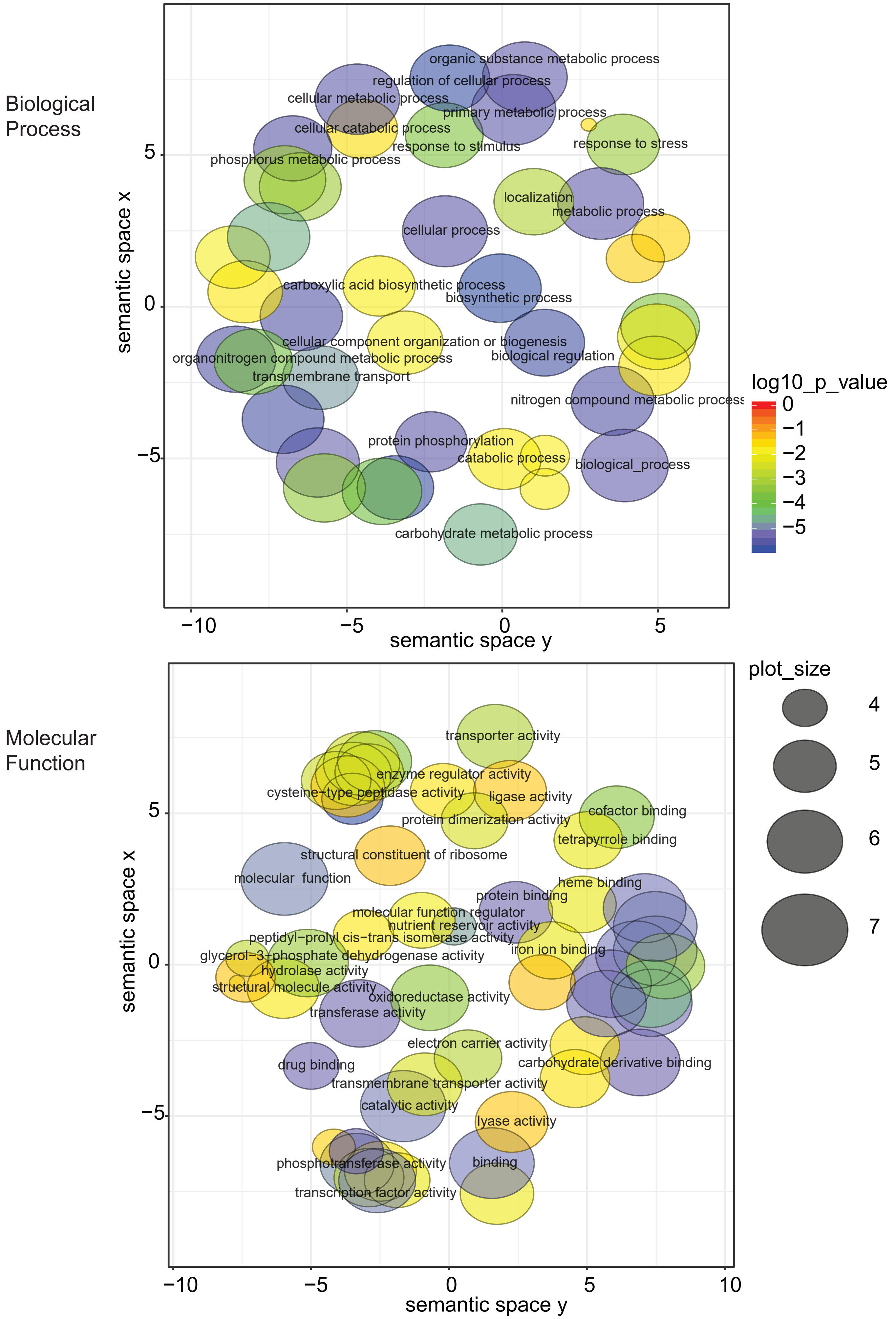

Supplementary Figure 22. The clustering of enriched GO terms of specific genes in No2127. The scatterplot shows the cluster representatives (i.e. terms remaining after the redundancy reduction) in a two dimensional space derived by applying multidimensional scaling to a matrix of the GO terms semantic similarities. Plot_size indicates the frequency of the GO term.

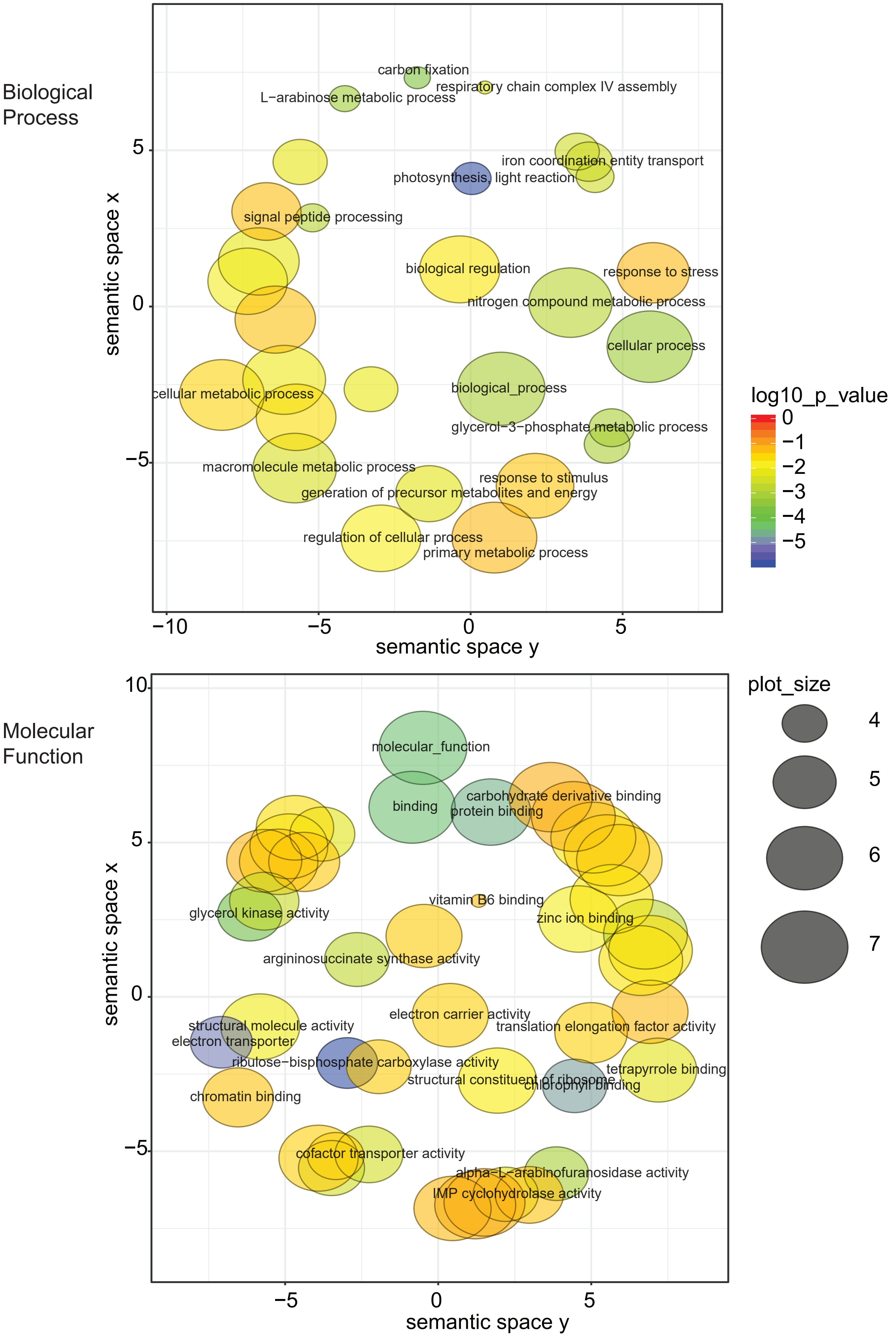

Supplementary Figure 23. The clustering of enriched GO terms of specific genes in Quinta. The scatterplot shows the cluster representatives (i.e. terms remaining after the redundancy reduction) in a two dimensional space derived by applying multidimensional scaling to a matrix of the GO terms semantic similarities. Plot_size indicates the frequency of the GO term.

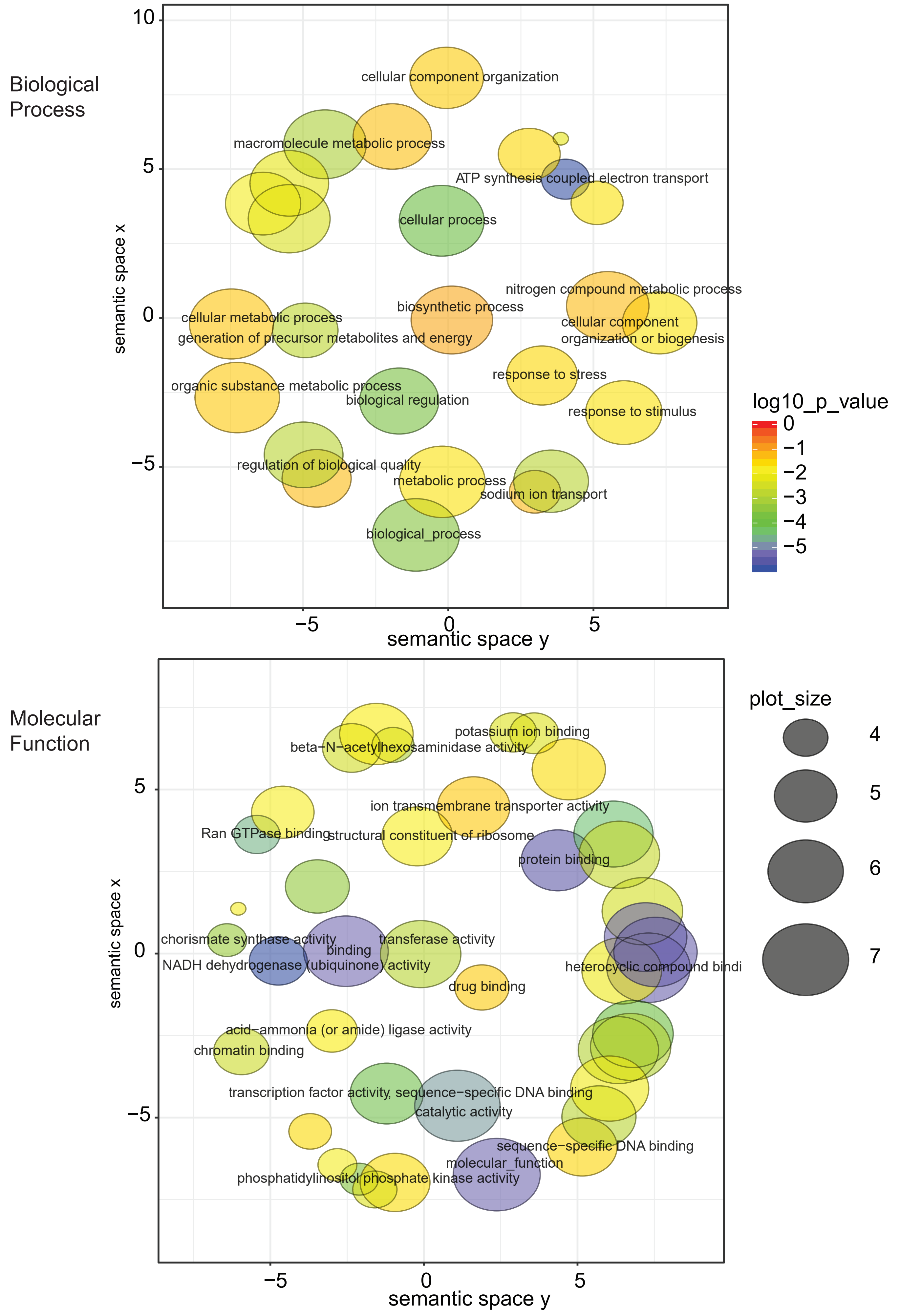

Supplementary Figure 24. The clustering of enriched GO terms of specific genes in Shengli. The scatterplot shows the cluster representatives (i.e. terms remaining after the redundancy reduction) in a two dimensional space derived by applying multidimensional scaling to a matrix of the GO terms semantic similarities. Plot_size indicates the frequency of the GO term.

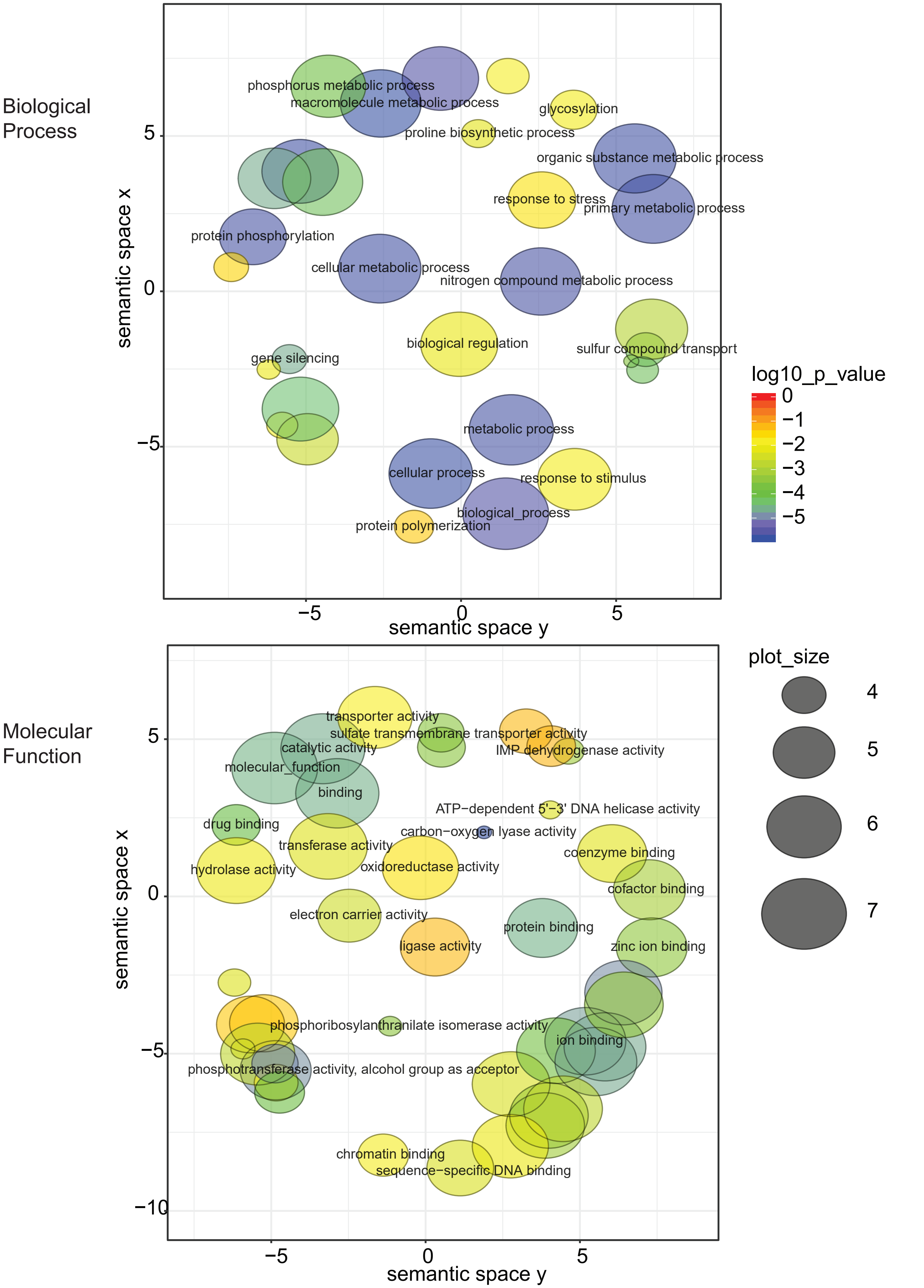

Supplementary Figure 25. The clustering of enriched GO terms of specific genes in Tapidor. The scatterplot shows the cluster representatives (i.e. terms remaining after the redundancy reduction) in a two dimensional space derived by applying multidimensional scaling to a matrix of the GO terms semantic similarities. Plot_size indicates the frequency of the GO term.

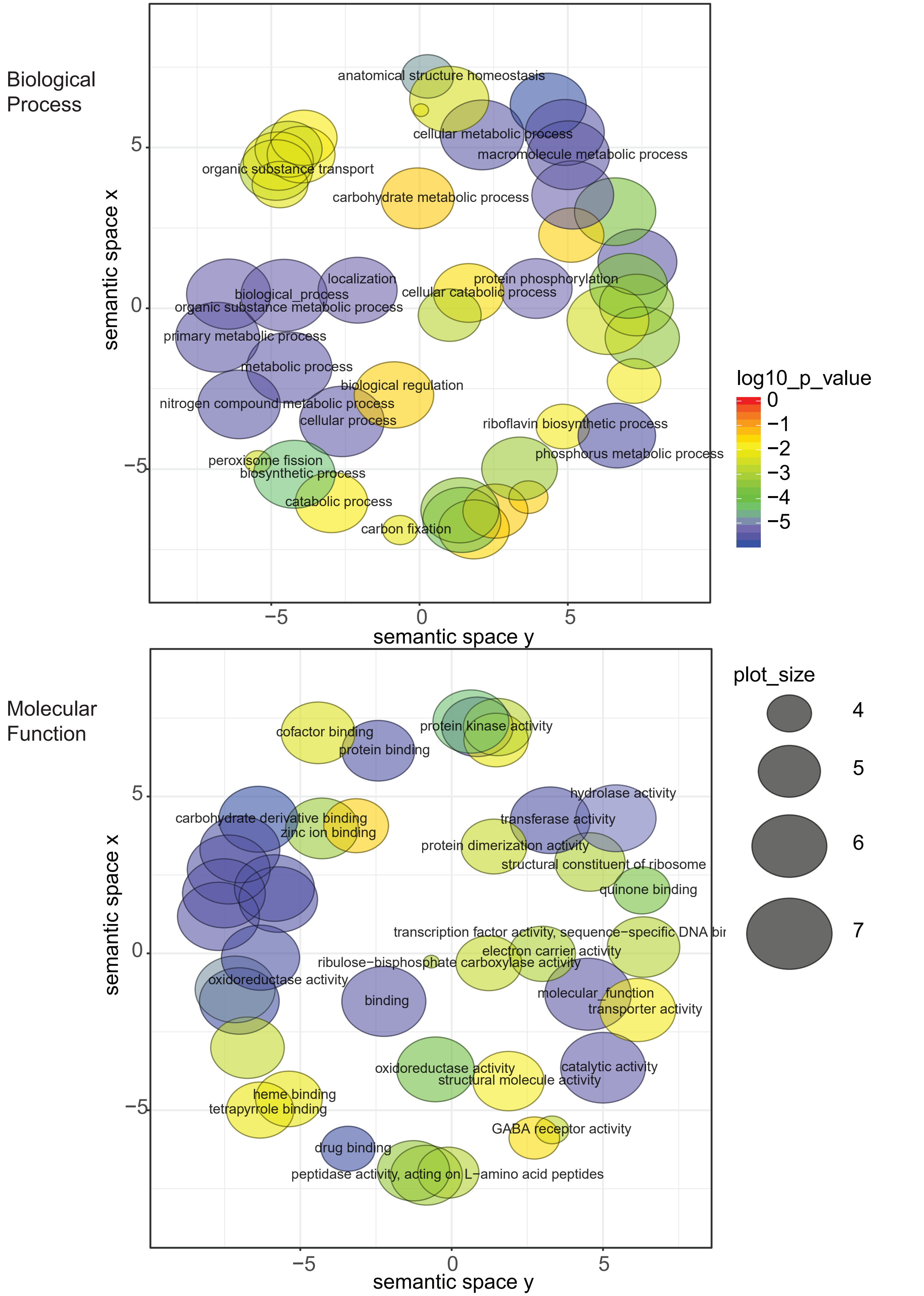

Supplementary Figure 26. The clustering of enriched GO terms of specific genes in Westar. The scatterplot shows the cluster representatives (i.e. terms remaining after the redundancy reduction) in a two dimensional space derived by applying multidimensional scaling to a matrix of the GO terms semantic similarities. Plot_size indicates the frequency of the GO term.

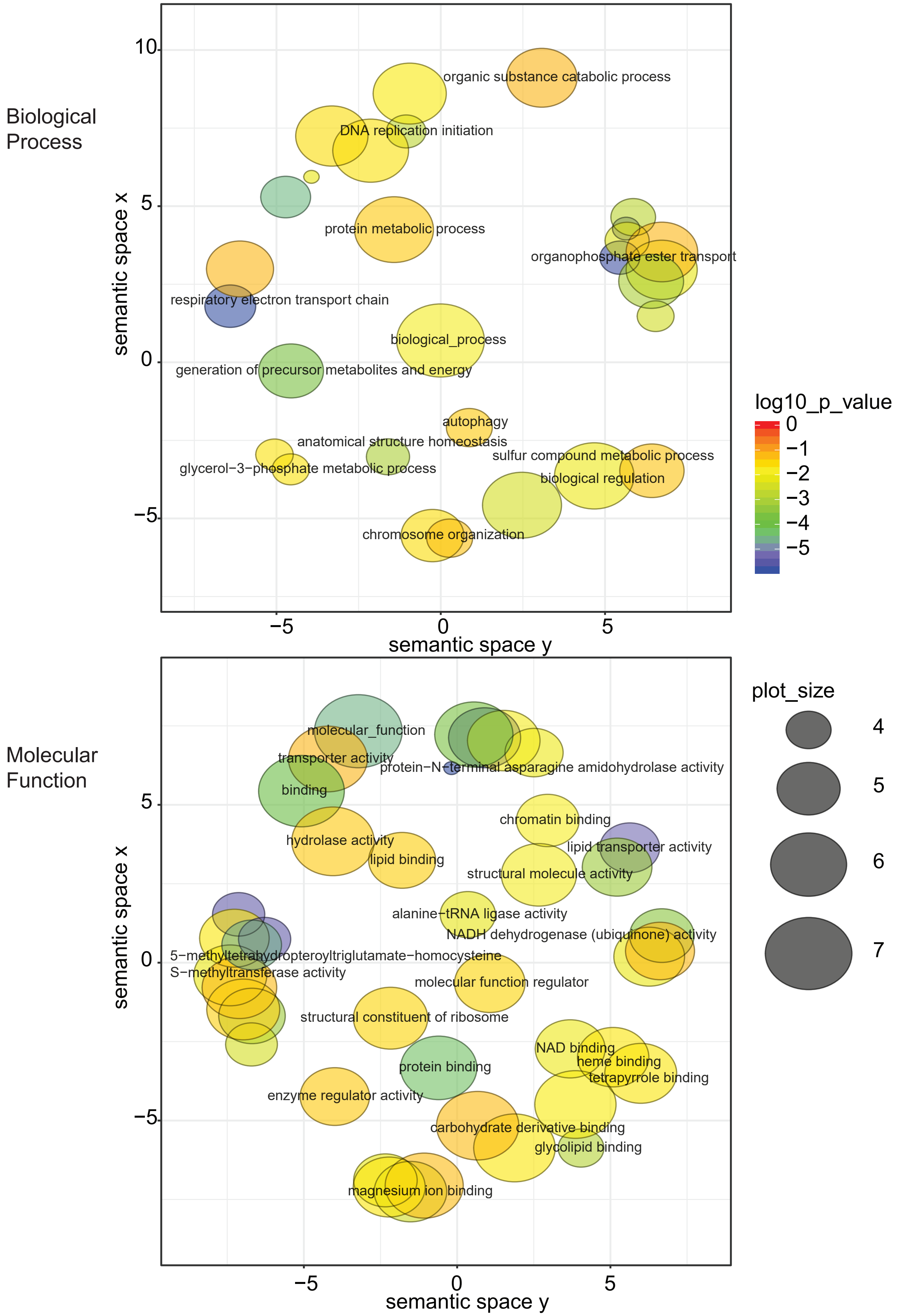

Supplementary Figure 27. The clustering of enriched GO terms of specific genes in Zheyou7. The scatterplot shows the cluster representatives (i.e. terms remaining after the redundancy reduction) in a two dimensional space derived by applying multidimensional scaling to a matrix of the GO terms semantic similarities. Plot_size indicates the frequency of the GO term.

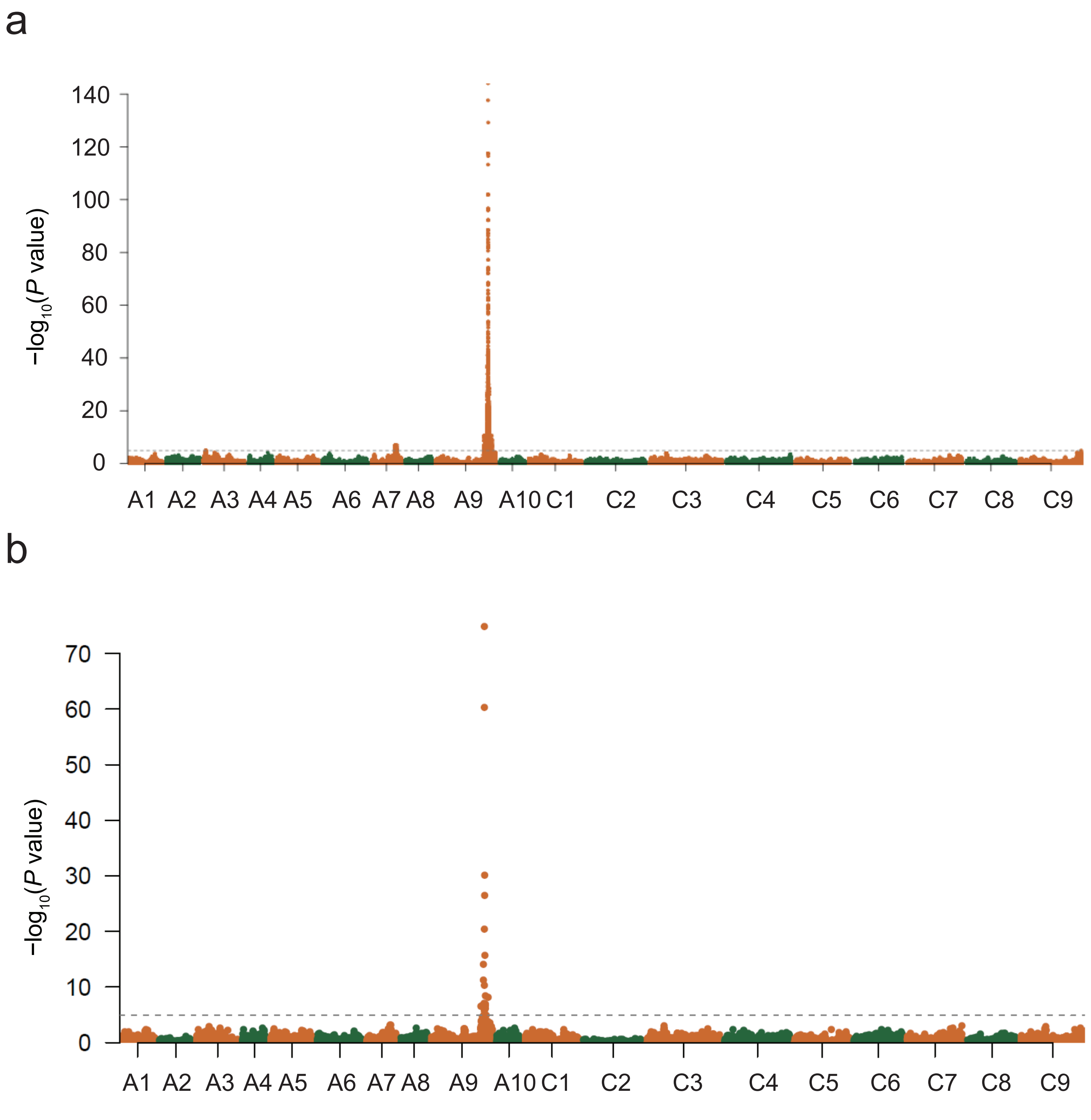

Supplementary Figure 28. Manhattan plots of SNP-GWAS and PAV-GWAS of silique length. a, Manhattan plot of SNP-GWAS of silique length for association using 3,971,412 SNPs. The gray dashed lines indicate the significance threshold ($-\log_{10}P$ = 4.94). **b,** Manhattan plot of PAV-GWAS of silique length for association using 27,146 PAVs. The gray dashed lines indicate the significance threshold ($-\log_{10}P$ = 4).

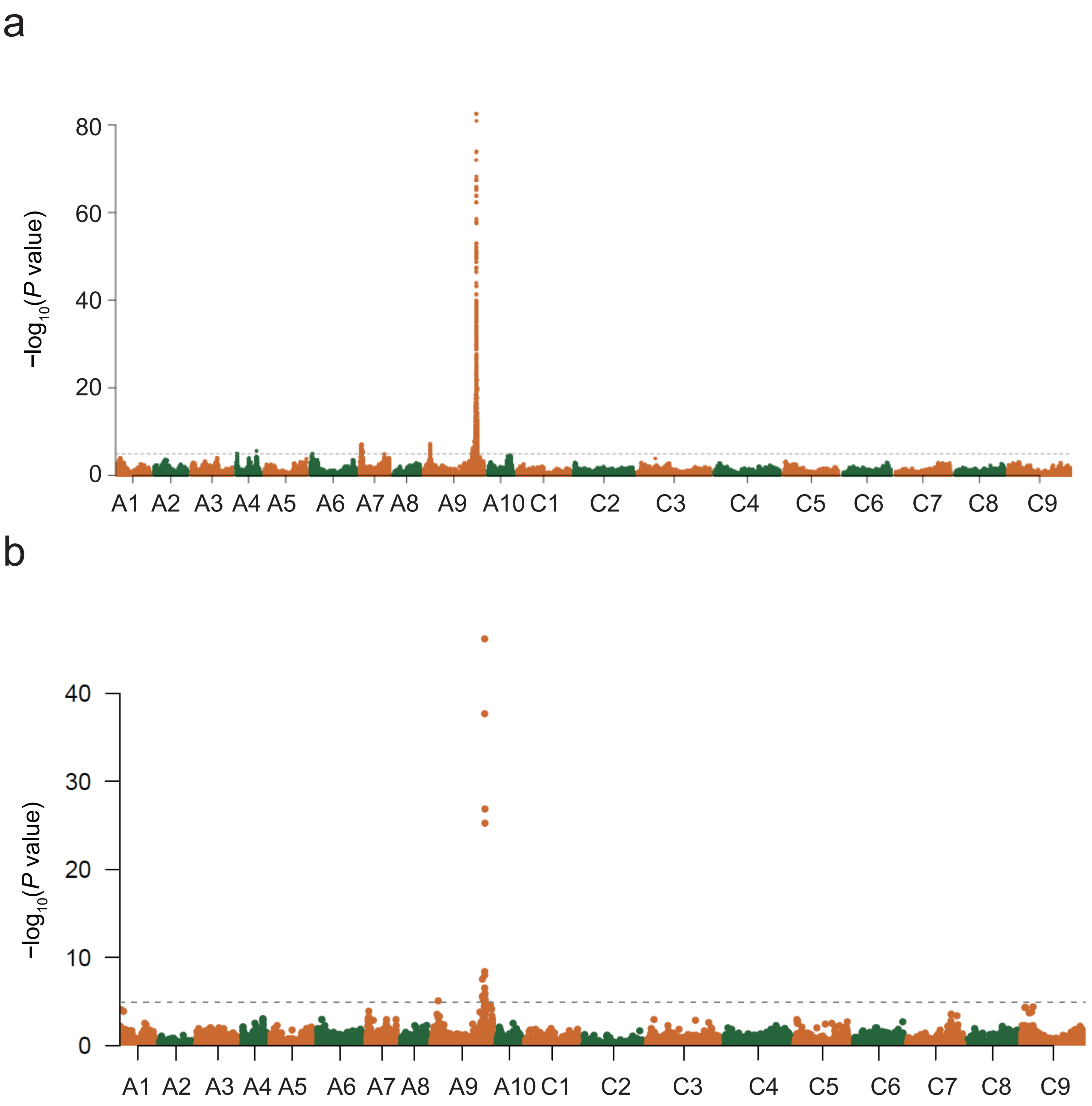

Supplementary Figure 29. Manhattan plots of SNP GWAS and PAV-GWAS of thousand seed weight. a, Manhattan plot of GWAS of thousand seed weight for association of 3,971,412 SNPs. The gray dashed lines indicate the significance threshold ($-\log_{10}P$ = 4.94). **b,** Manhattan plot of GWAS of thousand seed weight for association of 27,146 PAVs. The gray dashed lines indicate the significance threshold ($-\log_{10}P$ = 4).

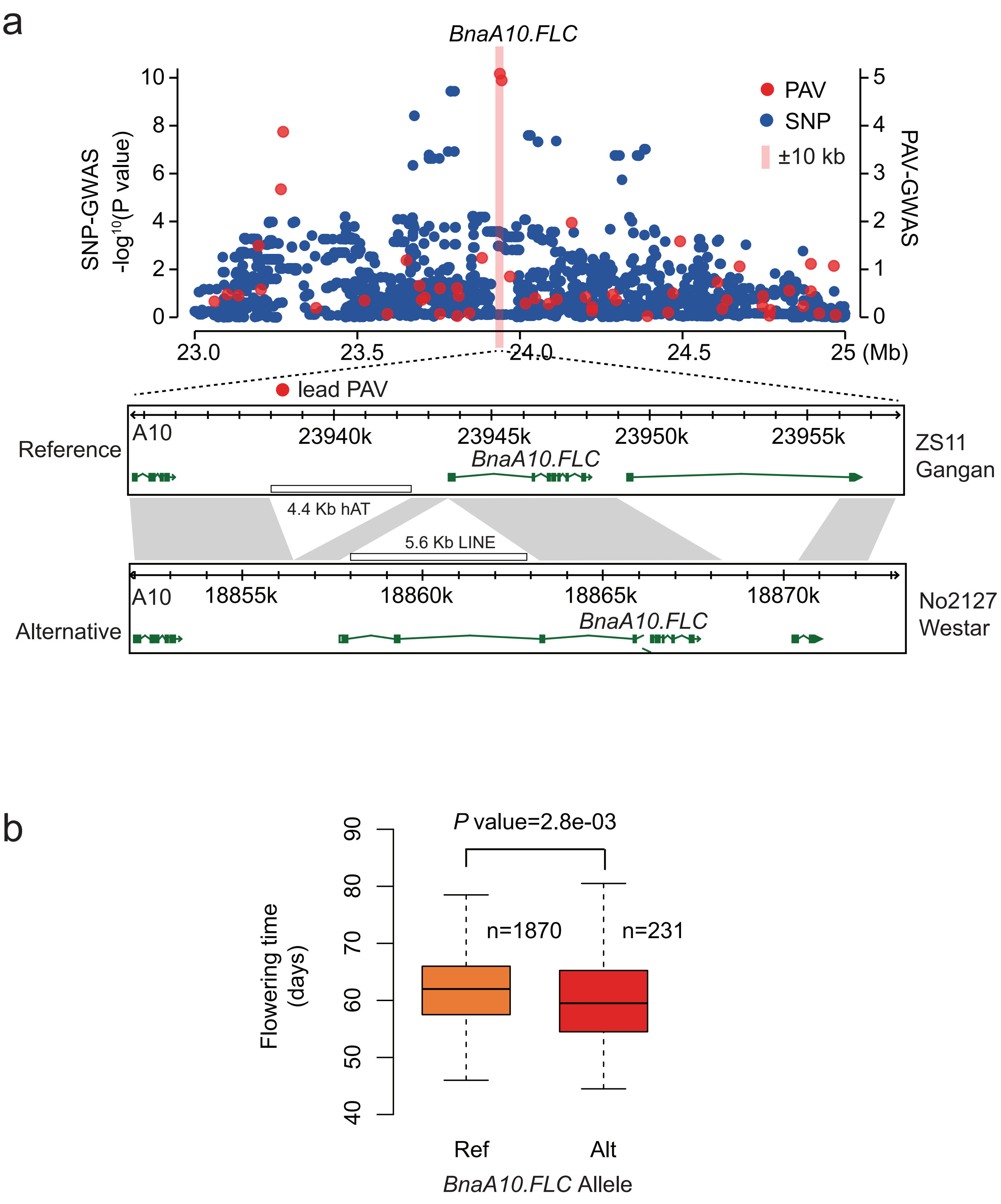

Supplementary Figure 30. The lead PAV on chromosome A10 identified by PAV-GWAS of flowering time in the NAM population. a, The Manhatton plots of SNP-GWAS (left vertical axis) and PAV-GWAS (right vertical axis) indicate the associations and the lead PAVs in the region from 23.0 to 25.0 Mb on chromosome A10. There were two PAVs in *BnaA10.FLC* (4.4 kb hAT and 5.6 kb LINE). **b,** The flowering time of RILs with different *BnaA10.FLC* genotypes in spring cropped environments. The flowering time of individuals with reference allele (with hAT insertion) was significantly longer than the individuals with Alt.

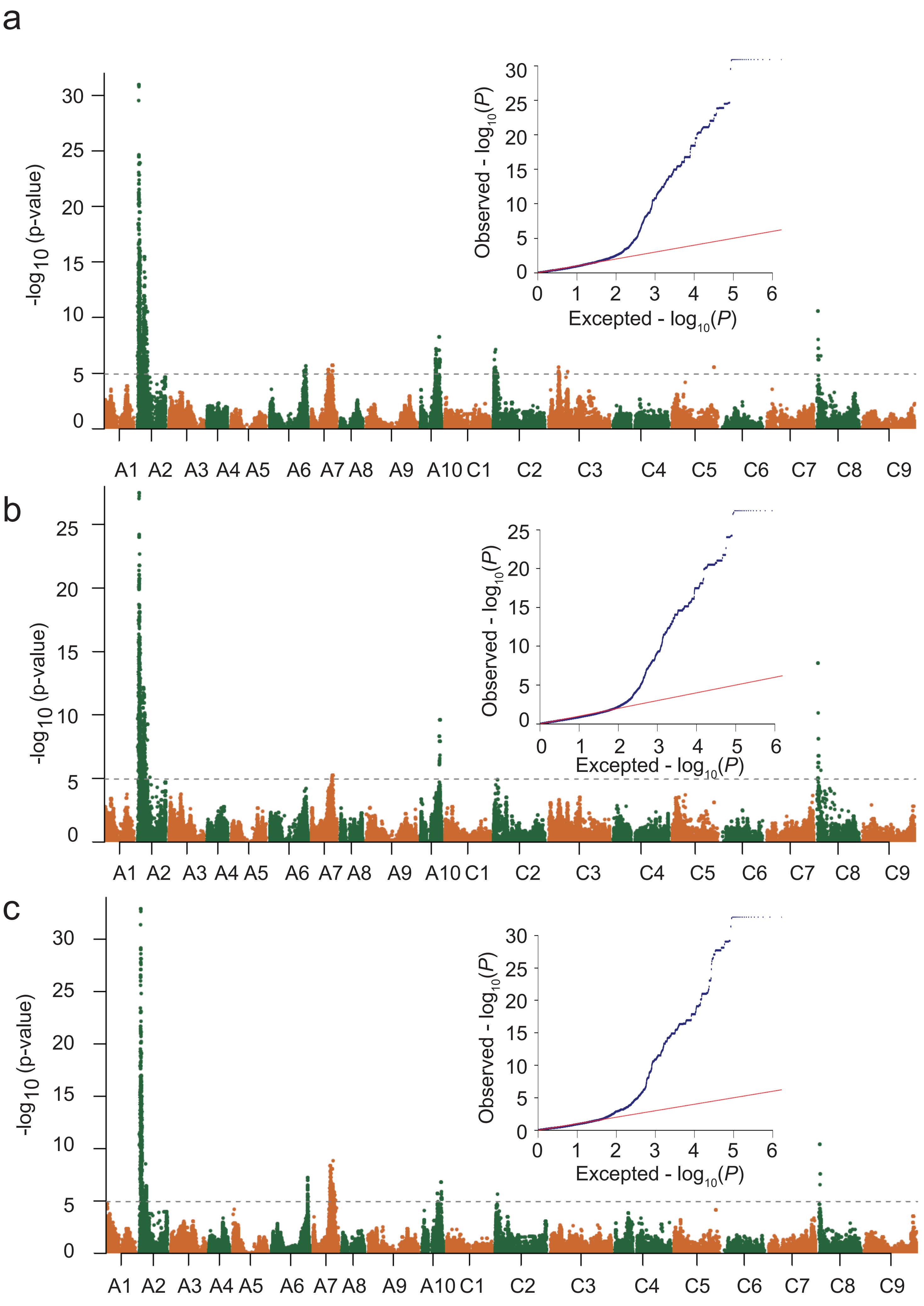

a
b
c
-log10 (p-value)
Observed - log10(P)
Excepted - log10(P)
A1 A2 A3 A4 A5 A6 A7 A8 A9 A10 C1 C2 C3 C4 C5 C6 C7 C8 C9

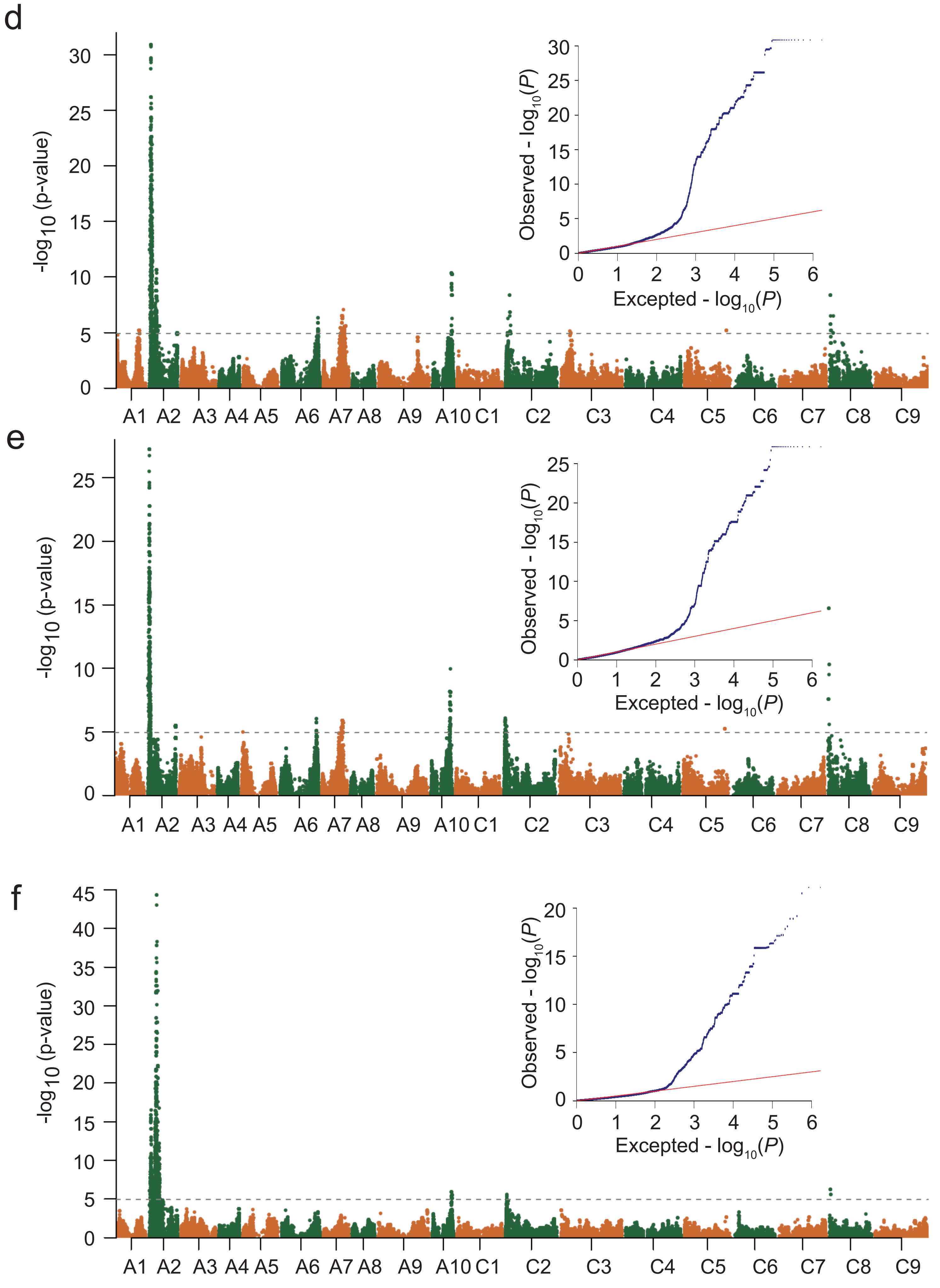

d
-log10 (p-value)
30
25
20
15
10
5
0
A1 A2 A3 A4 A5 A6 A7 A8 A9 A10 C1 C2 C3 C4 C5 C6 C7 C8 C9
Observed - log10(P)
Excepted - log10(P)
e
-log10 (p-value)
25
20
15
10
5
0
A1 A2 A3 A4 A5 A6 A7 A8 A9 A10 C1 C2 C3 C4 C5 C6 C7 C8 C9
Observed - log10(P)
Excepted - log10(P)
f
-log10 (p-value)
45
40
35
30
25
20
15
10
5
0
A1 A2 A3 A4 A5 A6 A7 A8 A9 A10 C1 C2 C3 C4 C5 C6 C7 C8 C9
Observed - log10(P)
Excepted - log10(P)

g

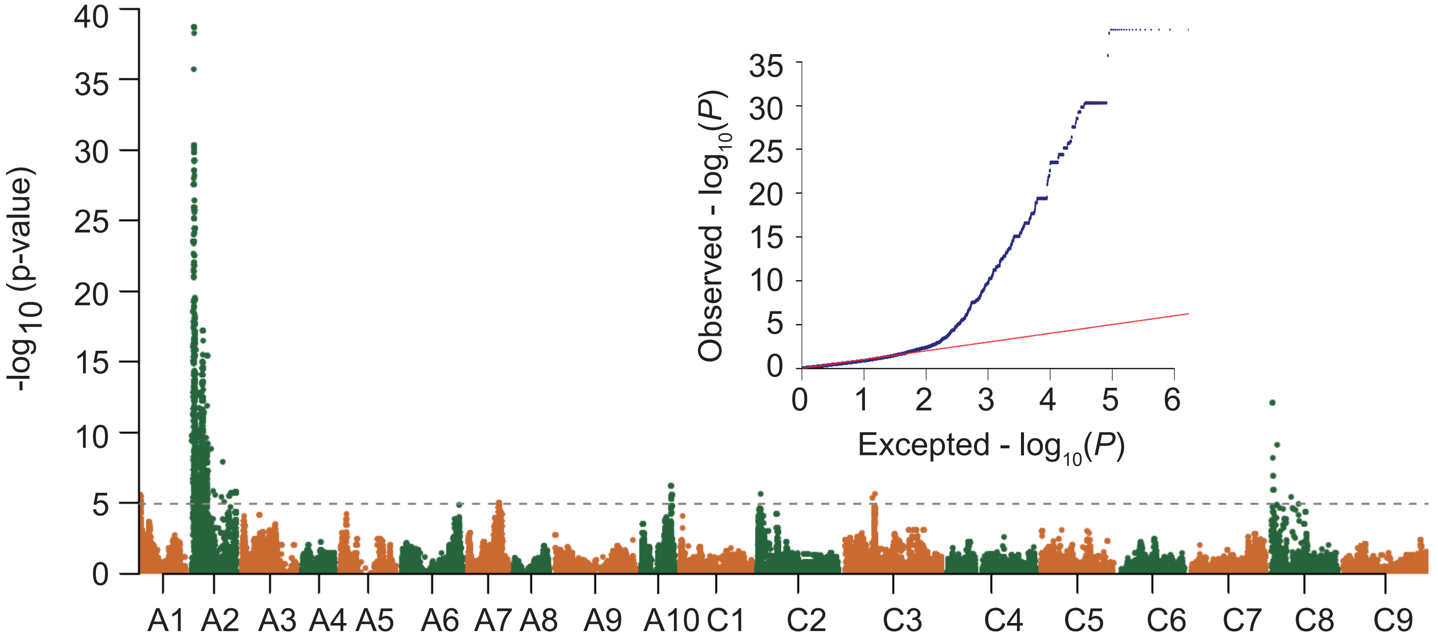

h

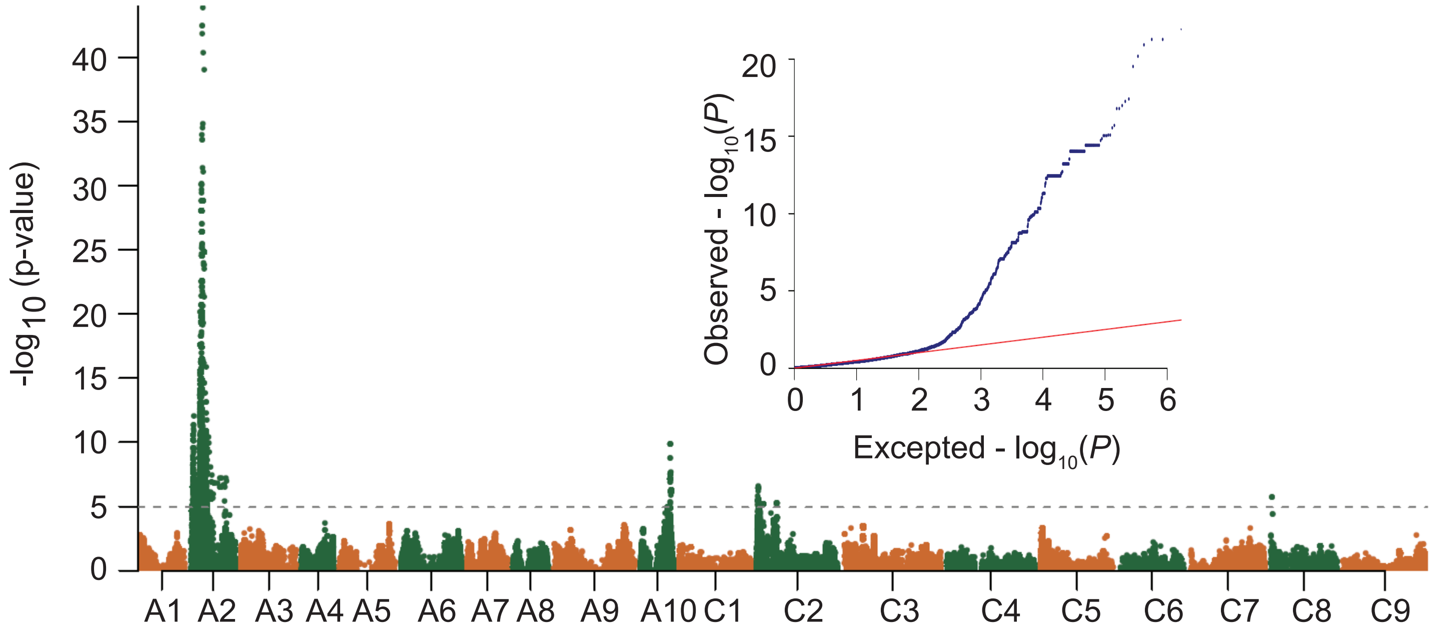

Supplementary Figure 31. The Quantile-quantile (Q-Q) and Manhattan plots of GWAS of flowering time with phenotypic data from multiple environments using genome-wide SNPs. The black dotted line marks the threshold for genome-wide significance ($-\log_{10}$(p-value) = 4.94). From a-h, Manhattan and Q-Q plots are the results of Wuhan in 2015 (winter environment), Caidian in 2015 (winter environment), Ezhou in 2015 (winter environment), Yangluo in 2015 (winter environment), Hezheng in 2016 (spring environment), Ezou in 2017 (winter environment) and Hezheng in 2017 (spring environment), respectively.

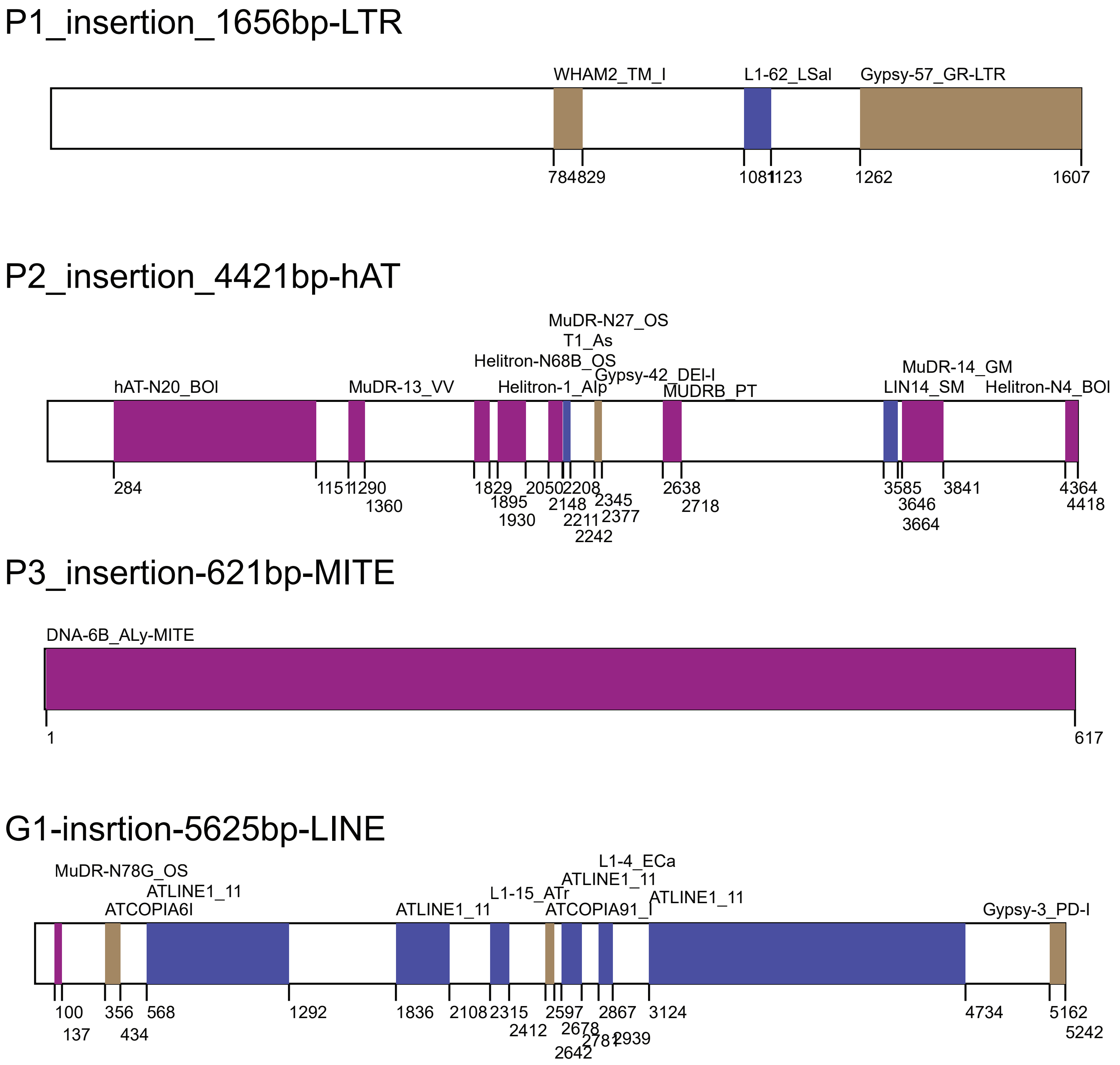

Supplementary Figure 32. Annotation and classification of transposable elements inserted in the promoter and genebody of *BnaA10.FLC*. The sequences of all insertions were used as queries to search a database of transposable elements using CENSOR. The four insertions are named as P (Promoter) 1-LTR, P2-hAT, P3-MITE and G (Genebody) 1-LINE based on their locations relative to *Bna10.FLC*.

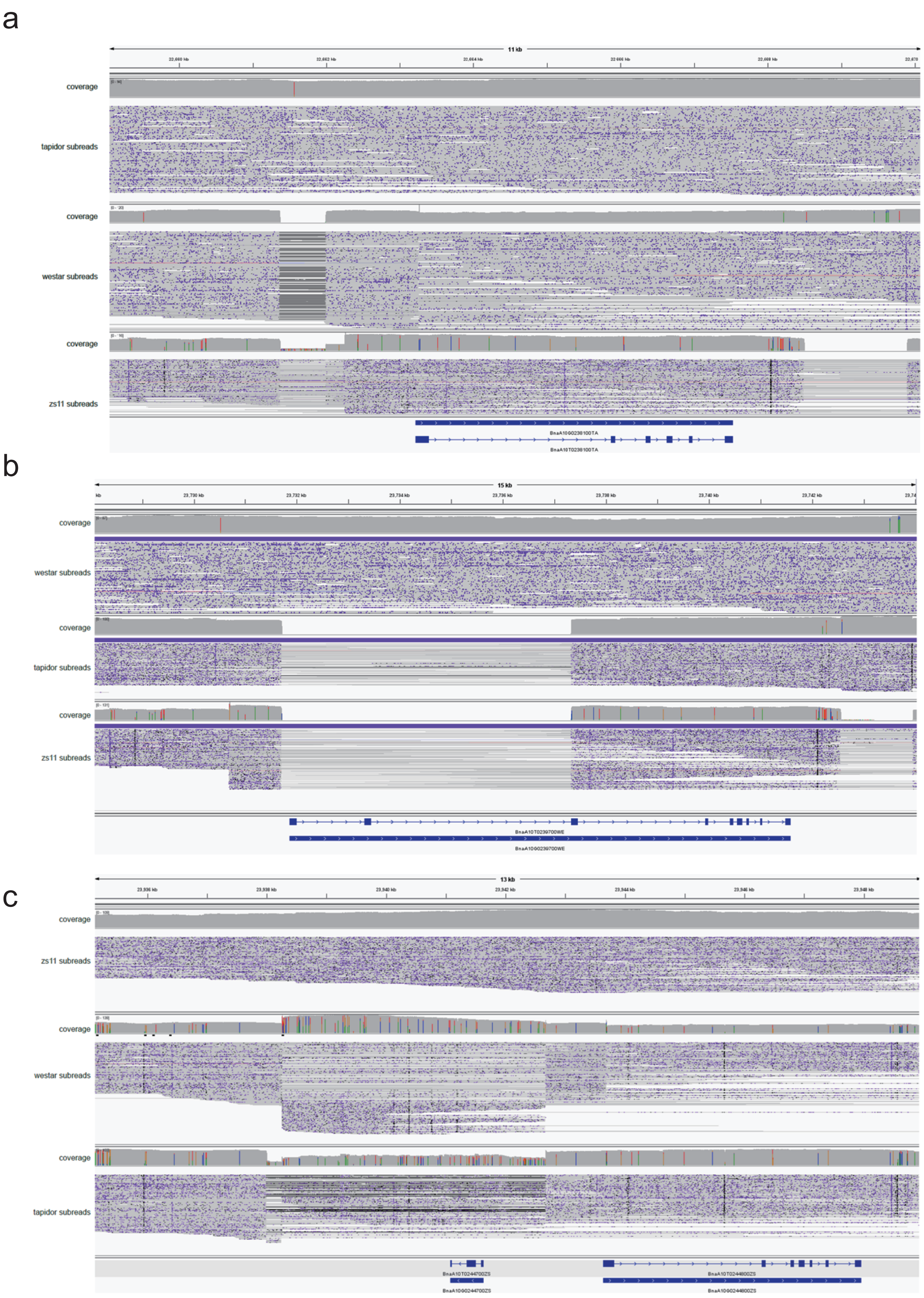

Supplementary Figure 33. **Validation of *BnaA10.FLC* structural variation using raw long reads. a,** Reads mapped to the Tapidor genome. **b,** Reads mapped to the Westar genome. **c,** Reads mapped to the ZS11 genome.

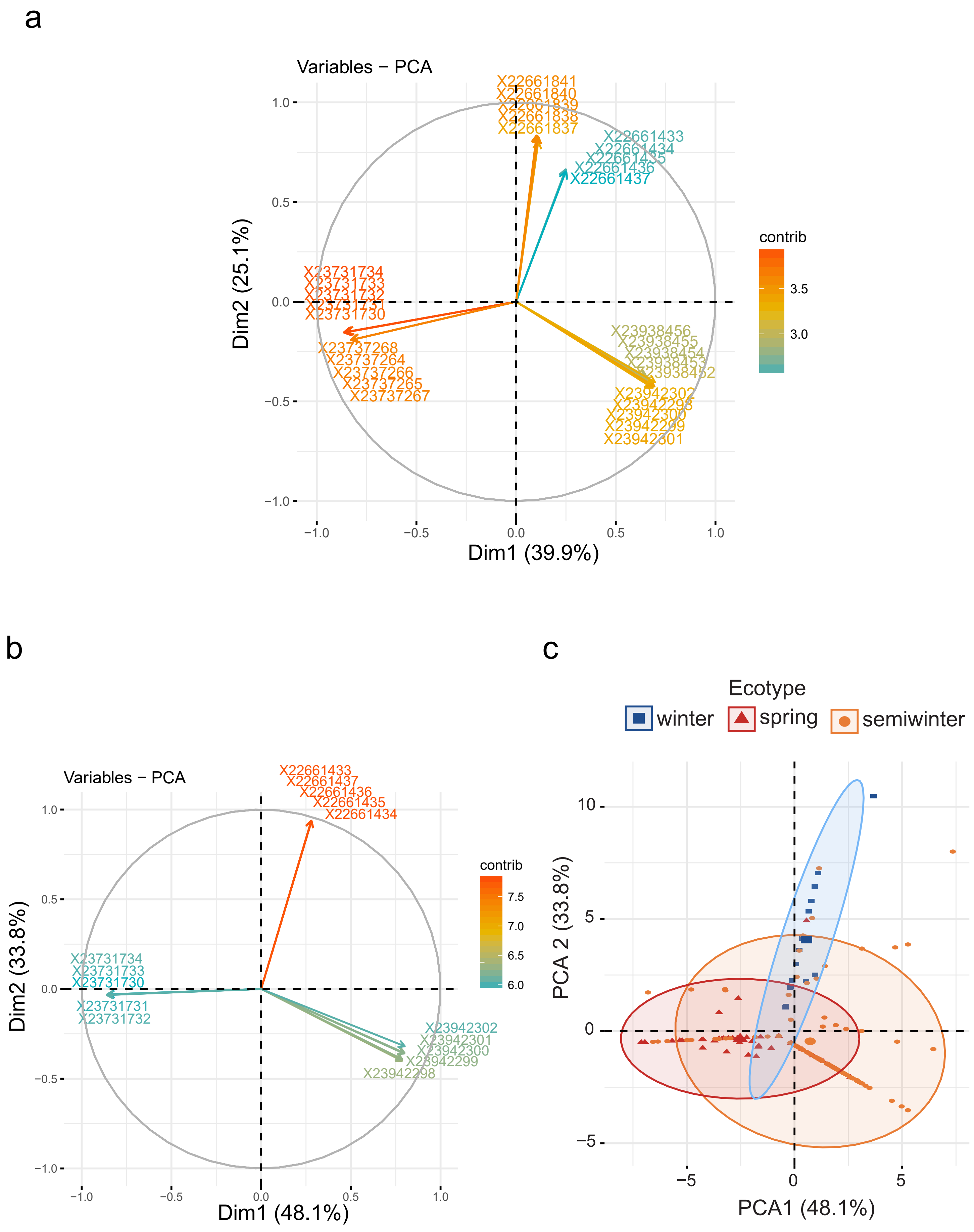

Supplementary Figure 34. The contribution of unique sites around *BnaA10.FLC* to the principal components. a, Visualization of the contribution of raw 30 candidate sites around *BnaA10.FLC*. **b,** Visualization of the contribution of final unique sites. **c,** PCA result of different ecotype *B. napus* resequence data. For a-c, the sample size (n) was 210.

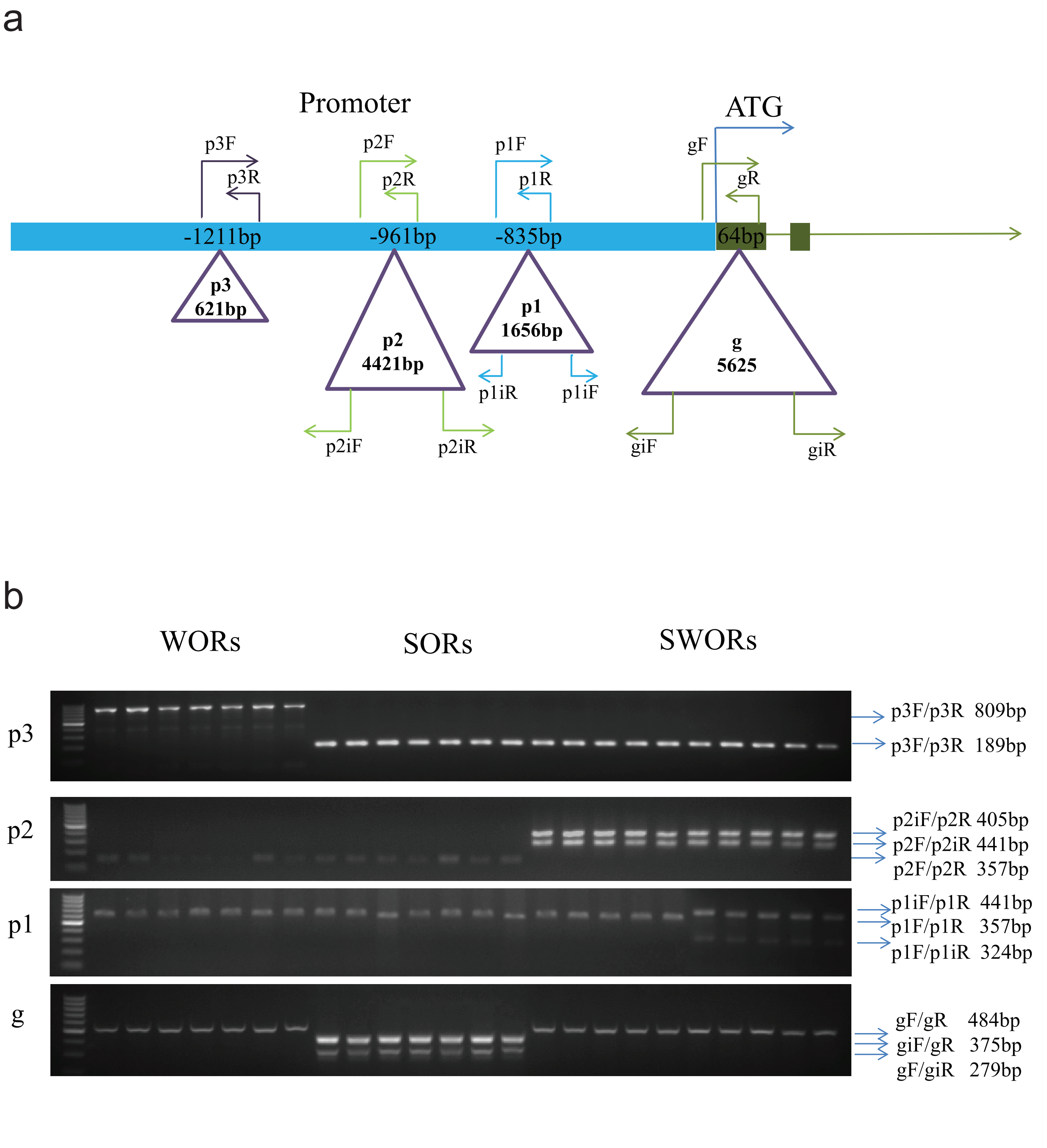

Supplementary Figure 35. PCR validation of structure variations in *BnaA10.FLC*. a, The locations of designed primers. **b,** PCR products amplified with multiple primers for each of the transposable elements in 21 *B.napus* accessions. From left to right, the ID of samples was Bn044, Bn025, Bn039, Bn022, Bn036, Bn026, g031, Bn037, No2127, g167, g168, g091, n003, Bn007, Bn049, g138, g015, g104, g107, Shengli, g179, g177, g052 and g080. These experiments were repeated once.

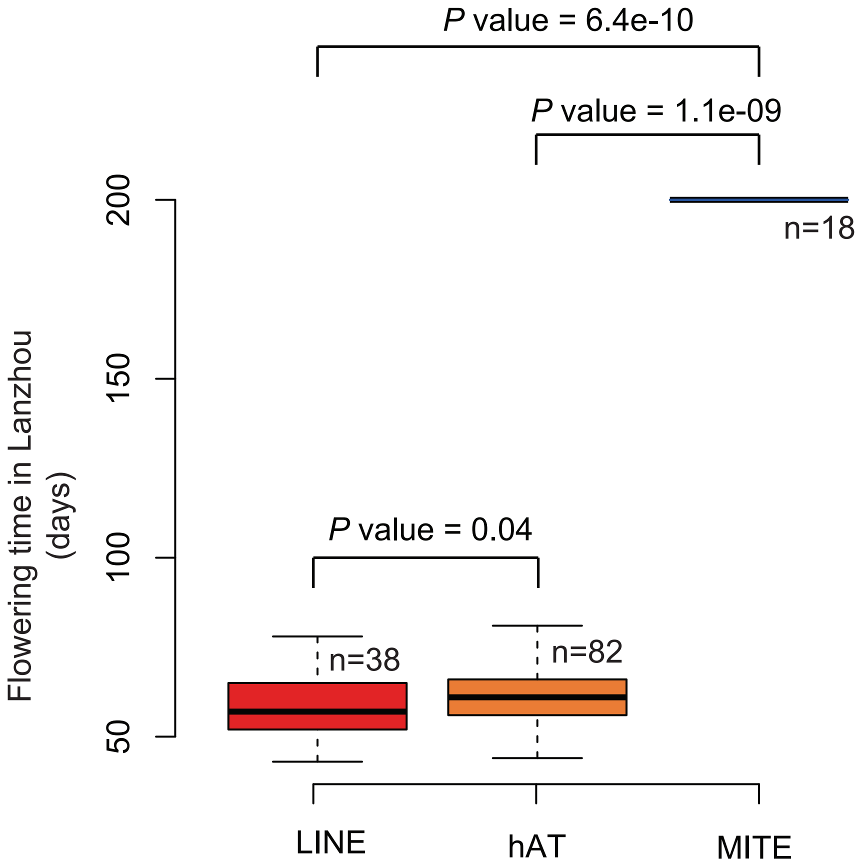

Supplementary Figure 36. Flowering time of natural population lines with different insertions in *BnaA10.FLC*. Flowering time data was collected in Lanzhou in 2014. The flowering time of individuals which can not flower was set to 200.

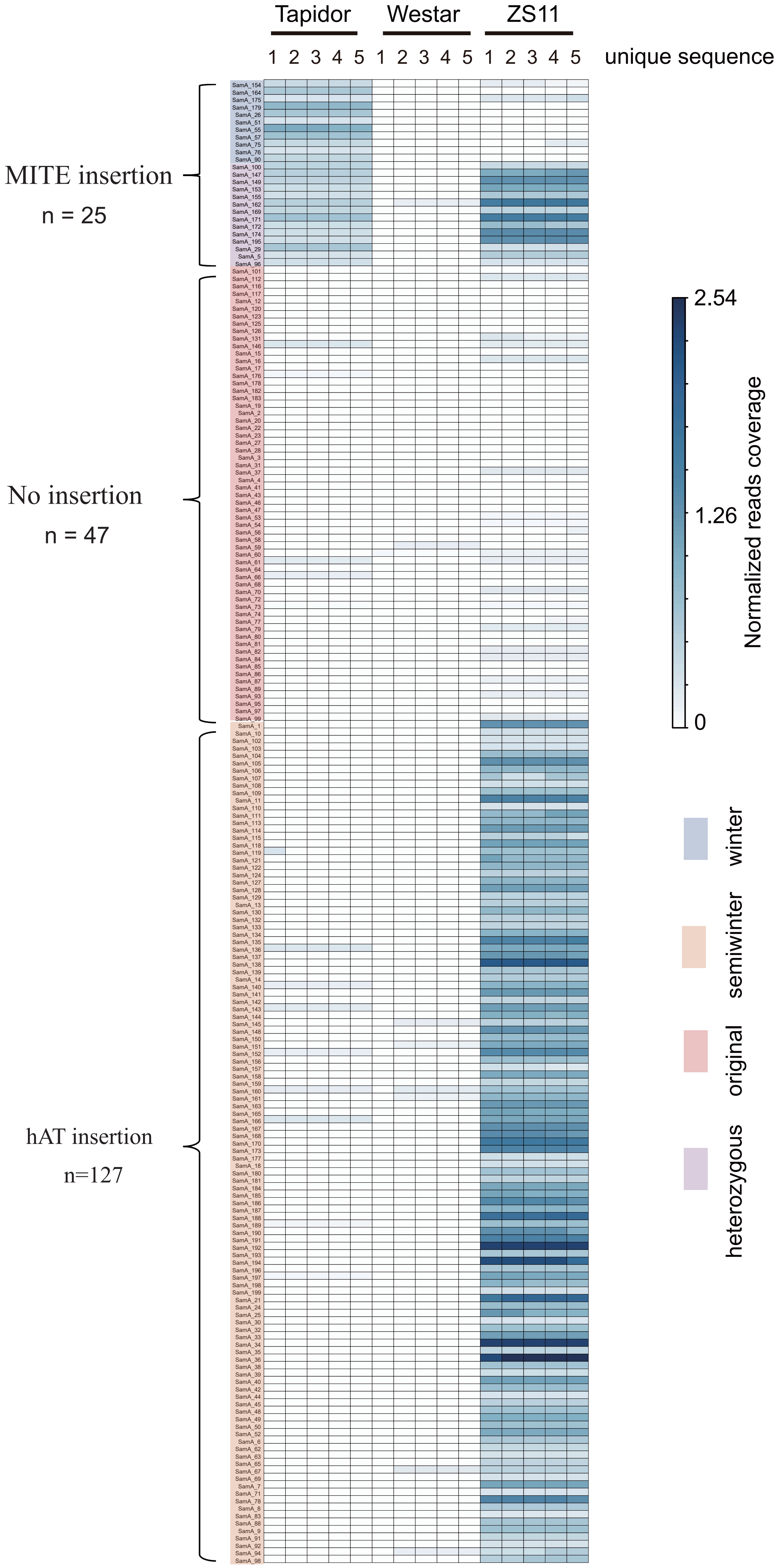

Supplementary Figure 37. The statistic of three insertions in *B. rapa* population (n=199). MITE insertion was detected in 25 *B. rapa* varieties. hAT insertion was detected in 127 *B. rapa* varieties. LINE insertion was not detected in the *B. rapa* population. Obeseved sites were the same as *B. napus*.

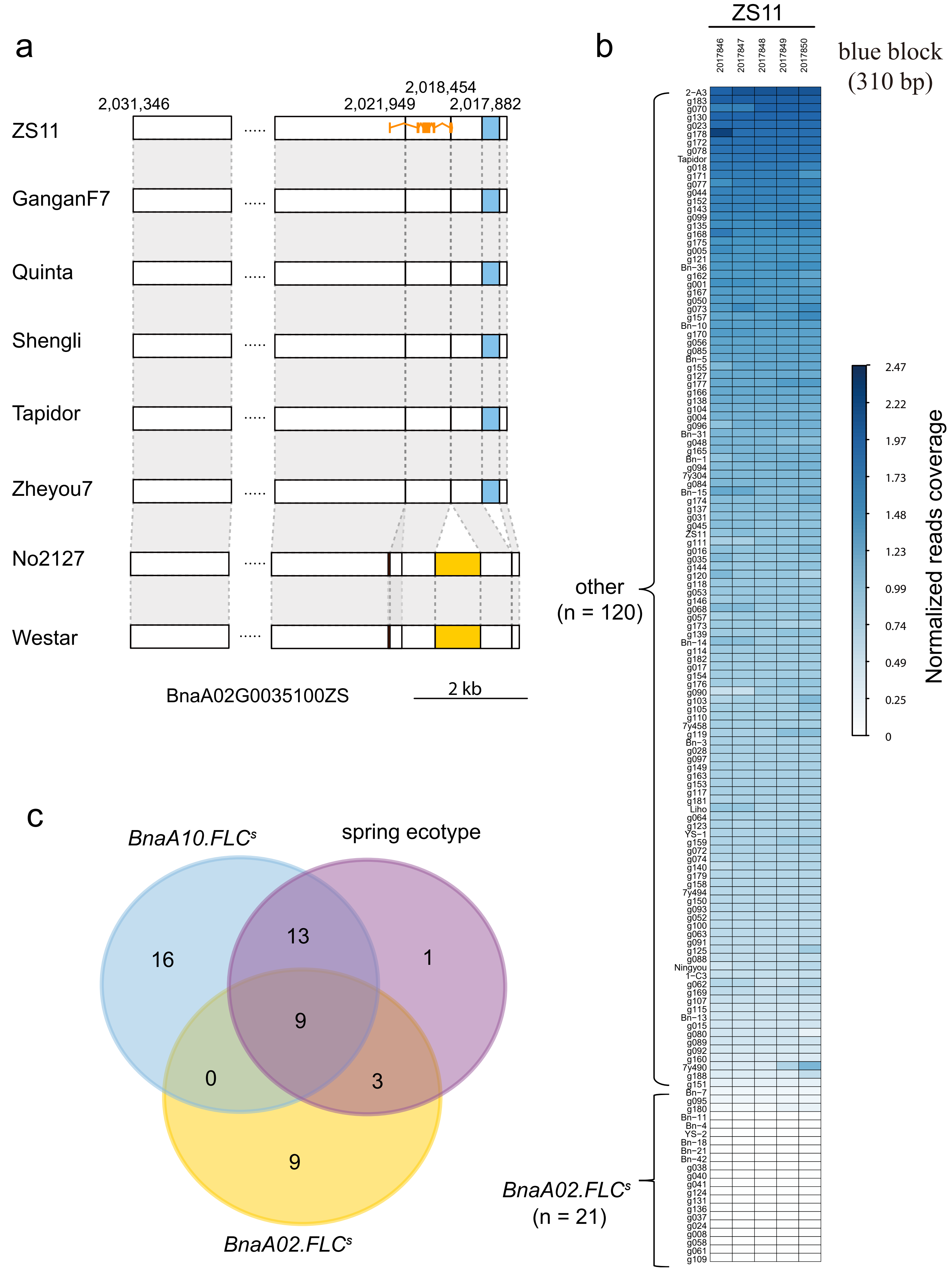

Supplementary Figure 38. The structrue variations of *BnaA02.FLC* in eight reference genomes and *B.napus* natural population. a, Structural variation of 8 *B. napus* genomes around *BnaA02.FLC*. An insertion about 824 bp (yellow block) was found in 2 spring *B. napus* on the last exon of *BnaA02.FLC*. Approximately 310 bp specific fragment (blue block) was present at 250 bp downstream of the *BnaA02.FLC* in the other 6 *B. napus*. **b,** Coverage of 310 bp specific fragments in the population. Accessions which do not have effective coverage (<0.25) for blue block specific fragments are considered to be spring-type *BnaA02.FLC* (*BnaA02.FLC*^{s}). **c,** The intersection of accession between *BnaA02.FLC*^{s} and spring ecotype *B. napus*. 57% of the *BnaA02.FLC*^{s} accessions are spring ecotype *B. napus*.

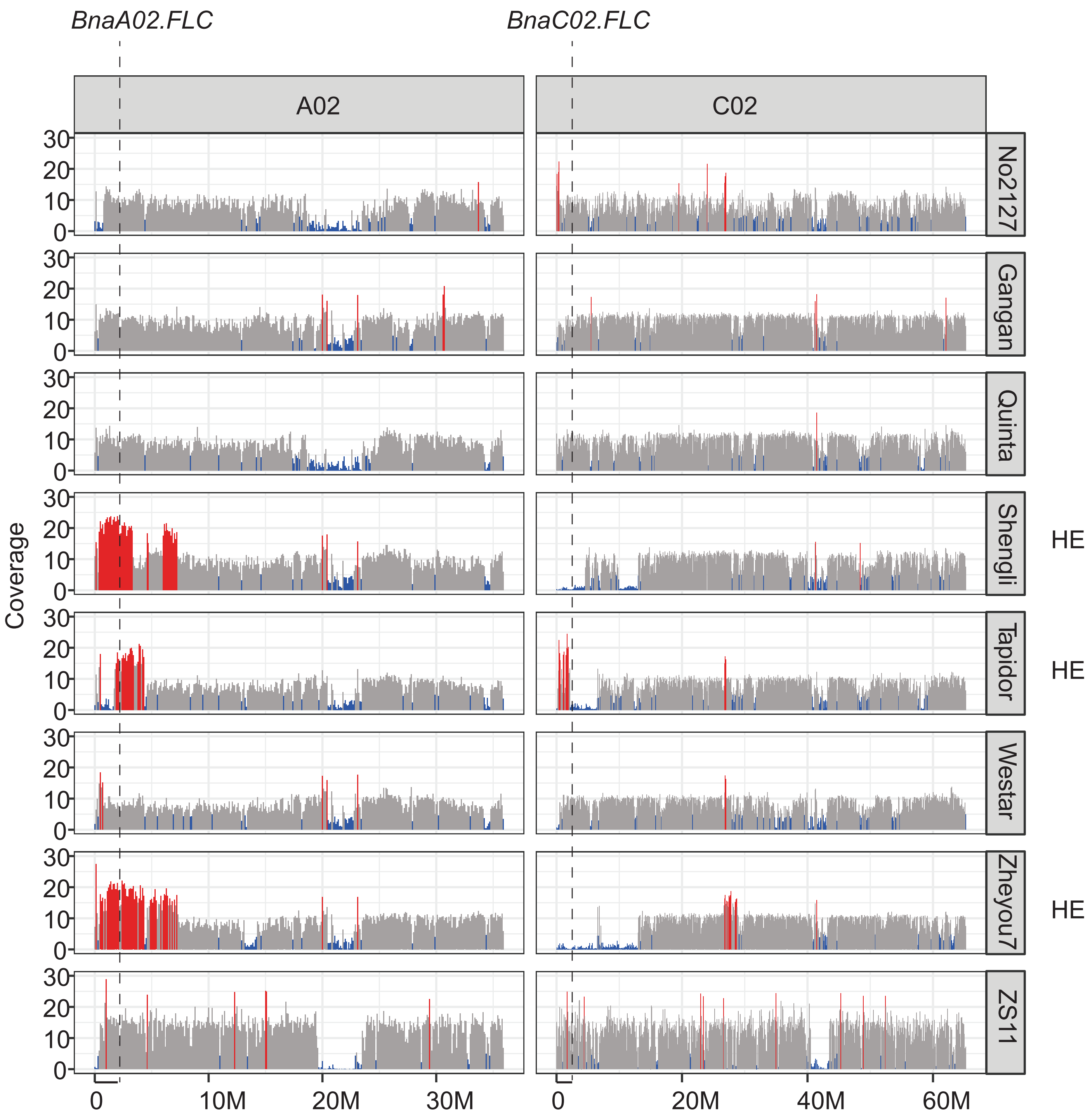

Supplementary Figure 39. Homologous exchange events detected between chromosome A02 and C02 of the eight *B. napus* genomes. Red bar represents a window whose coverage is larger than chromosome average coverage * 1.5. Blue bar represents a window whose coverage is less than 5. Homologous exchanges (HE) result in the presence of two copies of *BnaA02.FLC* and removal of *BnaC02.FLC* in the genomes of Shengli, Tapidor and Zheyou7.

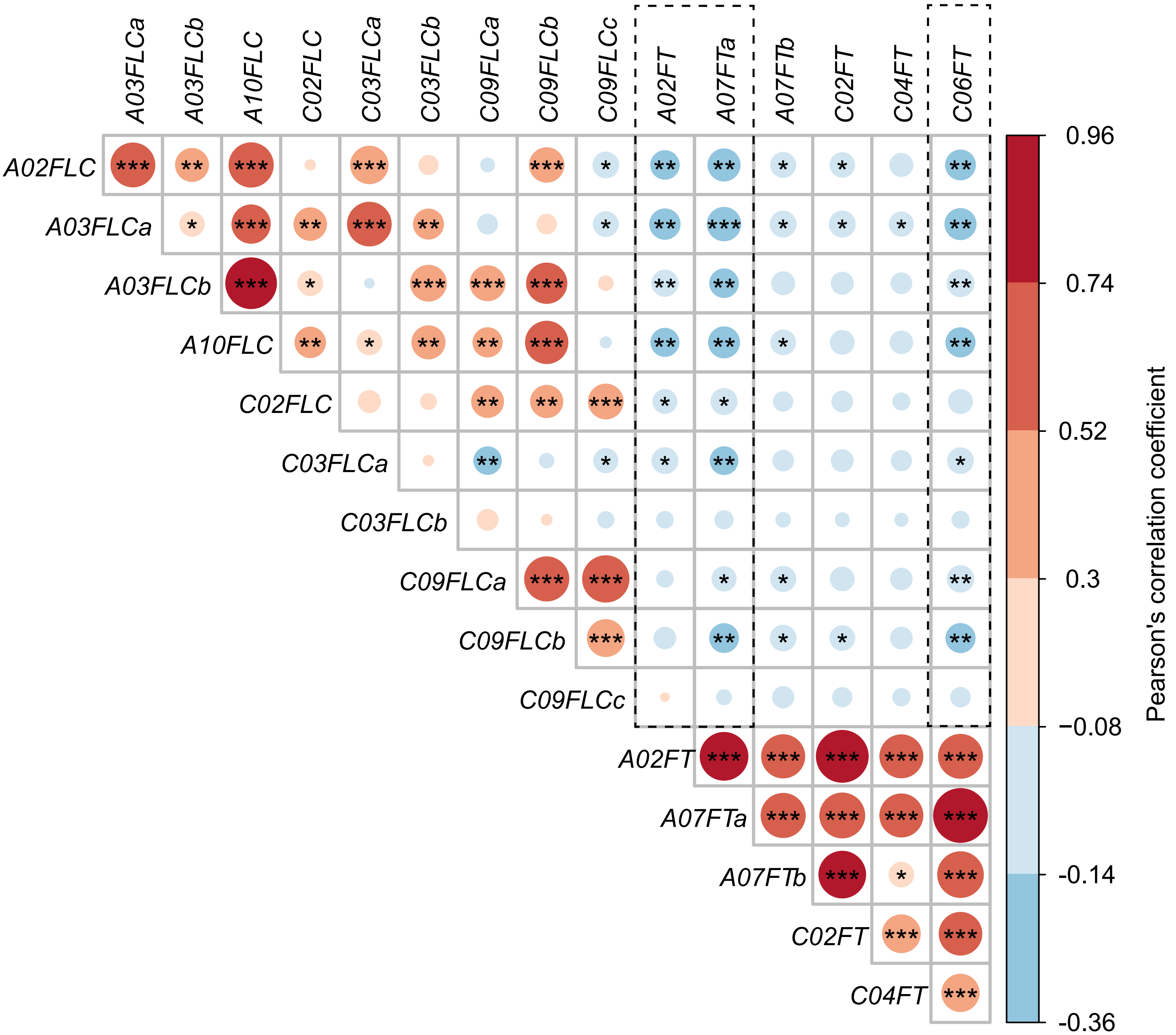

Supplementary Figure 40. Correlations between the expression levels of individual *FLC* and *FT* genes based on RNA-seq data. This color-coded correlation matrix illustrates pairwise correlations between the levels of gene expression in all individuals. Asterisks represent different significant levels (p-value < 0.1, 0.05 and 0.001).R package ‘rcorr’ was used to compute the matrix of Pearson's correlation coefficients. *BnaA02.FT*, *BnaA07.FTa* and *BnaC06.FT* were expressed *FT*s (average FPKM >=1 in each accession) and mainly associated *FT*s with *FLC*. Pearson's correlation coefficient was performed to determine the p-values. The sample size (n) was 80.

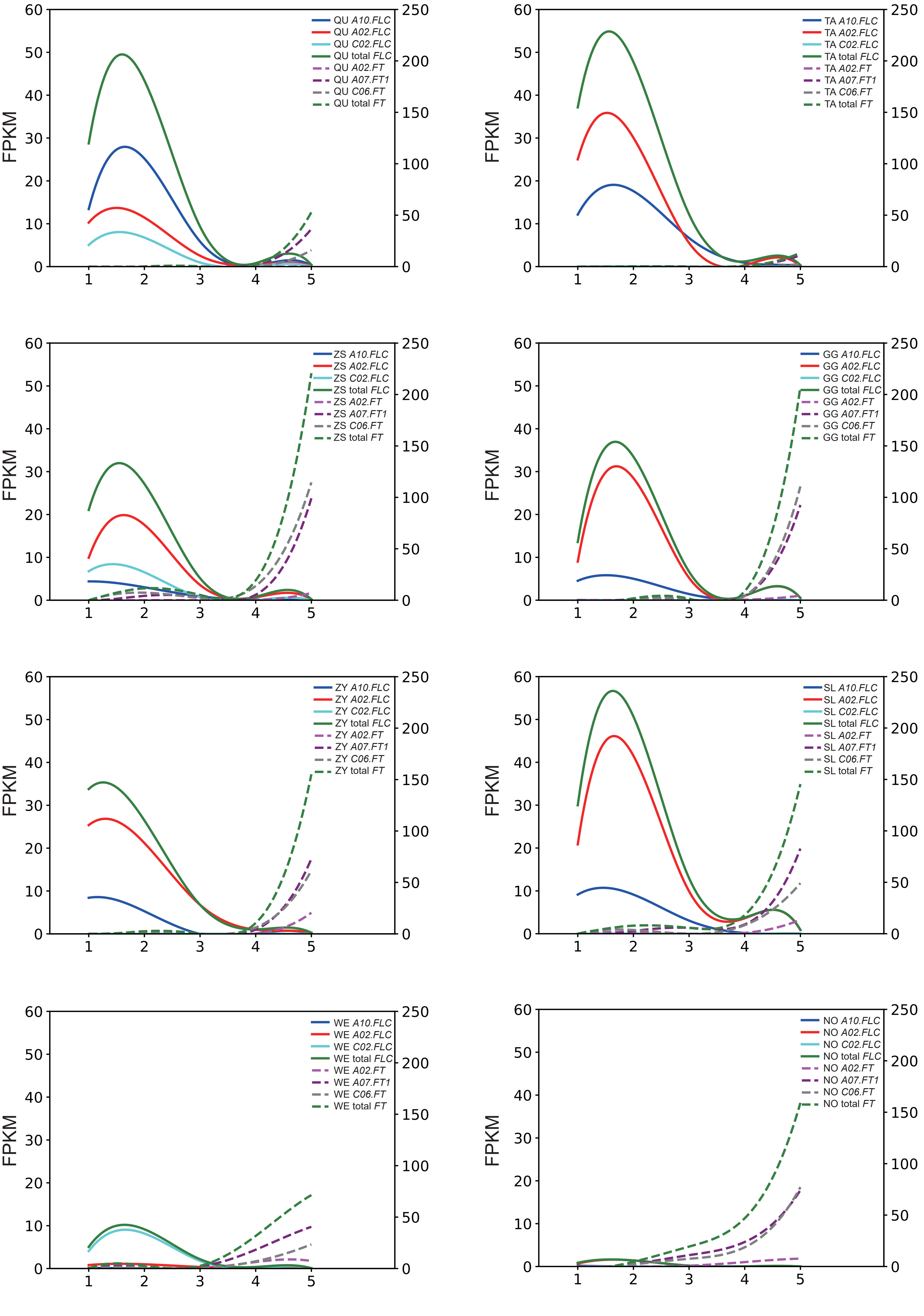

Supplementary Figure 41. Expression levels of individual *Bna.FLC*s and *Bna.FT*s in the eight reference accessions before and after vernalization as detected by RNA-seq. The left Y-axis and the right Y-axis are *Bna.FLCs* and *Bna.FTs* expression levels, respectively. The X-axis 1-5 indicates five stages T0-T4.

Supplementary Notes

RNA-seq. For each accession, tissues including roots, stems, leaves before vernalization, leaves after vernalization, buds, flowers and siliques 25-day-after pollination were pooled in one RNA-seq library. Eight RNA-seq libraries were sequenced by the Illumina HiSeq platform with 150-bp paired-end reads for gene annotation. RNA-seq for leaves of all eight sequenced rapeseed accessions at five stages with one month interval including T0 stage before vernalization (the lowest temperature was higher than 10□), T1-T3 stage during vernalization (the lowest temperature was lower than 10□), and T4 stage post vernalization (low temperature lower than 10□ more than 100 days).

BioNano sequencing and assembly. High-molecular weight DNA was isolated and labelled from leaf tissue of three-week old *B. napus* plants according to BioNano's standard protocols using the single-stranded nicking endonuclease Nt.BspQI. Labelled DNA was imaged automatically using the BioNano Irys system and *de novo* assembled into consensus physical maps using the BioNano IrysView analysis software (URL). The final *de novo* assembly used only single molecules with a minimum length of 150 kb and eight labels per molecule. PacBio-BioNano hybrid scaffolds were identified using IrysView's hybrid scaffold alignment subprogram.

Merge the assemblies of Canu and Falcon. MUMmer[1] was used to perform reciprocal alignment between Falcon[2] and Canu[3] assemblies with the parameters "-l 90 -d 40". Then the unaligned contigs in Canu assembly were filtered by Illumina reads coverage (mean coverage < 50X) to identify the final unique sequences in Canu assembly. The final assembly was generated by combining the unique sequences in Canu assembly and the whole Falcon assembly.

Annotation of repeats. The method of repetitive sequence annotation was divided into two types: homologous sequence alignment and *de novo* prediction. Homologous sequence alignment was based on repetitive sequence database (RepBase database)[4] and software Repeatmasker[5] to identify sequences similar to known repetitive sequences. *De novo* prediction was used to build up the *de novo* repetitive sequences database using software

LTR_FINDER[6], PILER[7], RepeatScout[8] and RepeatModeler (http://www.repeatmasker.org/RepeatModeler.html). For predicted database, we carried out the improvement of database by RepBase Database with Uclust based on 80-80-80 rules and Repeatmask. In addition, *de novo* prediction could obtain tandem repeat in genome with TRF[9].

Gene annotation. Prediction of gene structure was combined with multiple prediction methods, mainly homologous prediction, *de novo* prediction and evidence-based prediction. Augustus[10], GlimmerHMM[11] and SNAP[12] were used for *de novo* prediction depending on the genome sequence data statistical characteristics. Homologous prediction was performed by aligning proteins of homologous species (*A. thaliana*, *B. oleracea* capitata, *B. oleracea* TO1000, *B. rapa* Chiifu, *B. napus* Darmor and *B. napus* ZS11) through Genewise[13] to predict gene structure. For each accession, RNA-Seq data of mixed tissues were used as transcript evidence. According to the prediction and transcriptome comparison data, we used EVidenceModeler[14] to integrate gene sets by different methods into a non-redundant and more complete gene set. Finally, we used PASA[14] to correct the EVM annotation based on transcriptome assembly, and added UTR, variable clipping and other information to obtain the final gene set.

ncRNA annotation. The annotation of non-coding RNA includes tRNA, rRNA, miRNA and snRNA. According to the structure characteristics of tRNA, tRNAscan-SE[15]was used to obtain tRNA in the genome. Considering that rRNA was highly conserved, blast was employed to identify rRNA sequences by aligning to related species. Sequences of miRNA and snRNA were obtained by Rfam family covariance model using INFERNAL[16]

Centromere regions. Centromere-specific repeats (CentBr1 and CentBr2)[17] were aligned to the ZS11 genome by BLAST (E-value: 1e^{-5}) to identify centromere-related sequences.

Hi-C data processing and analysis. The Hi-C reads were mapped to ZS11 genome and filtered using Juicer pipeline[18]. The following read pairs were removed: duplicated and near-duplicated read pairs, read pairs mapped to the same fragment or mapping quality score below 30. The contact matrices were built at 10-, 50-, 100- and 500-kb resolutions using Juicer_tools[19]. Normalized contact matrices were produced at all resolutions with the KR

method[20]. For compartment analysis, each chromosome was divided into consecutive 500-kb regions from normalized contact matrices. The eigenvectors of all regions were analyzed using the Juicer_tools. Finally, all regions were aligned to A/B compartment according to the gene density due to the fact that genomic regions belonging to the A compartments usually contain more genes than those of B compartments[21].

Genome blocks construction. We used the same method as Wang et al.[22] to construct the genome blocks. We first got the syntenic blocks of the ZS11 genome and *A. thaliana* genomes by MCScanX with default parameters[23]. Then LF, MF1 and MF2 were separately classified in A and C sub-genome according to gene retention of these blocks.

Divergence time. In order to ensure the high homology of gene pairs in K_s calculation, we searched for orthologous pairs and paralogous pairs within species based on colinear region and reciprocal best blast hit (RBH). In order to call ortholog blocks, we performed all-against-all blastp ($E < e^{-10}$). We identified putative homologous chromosomal regions using MCScanX based on blastp results. Each block contained at least 5 genes, and the maximum gap between genes was allowed to be less than 25. On the other hand, Python scripts (https://github.com/peterjc/galaxy_blast/blob/master/tools/blast_rbh/blast_rbh.py) were used to obtain RBH between two genomes. Finally, the intersection of the gene pairs in homologous region and RBH gene pairs were used for K_s analysis. For homologous gene pairs, we screened sequence difference by protein and CDS alignment with ParaAT[24]. For orthologous gene pairs and paralogous gene pairs (*B. napus*, *A. thaliana* Initiative A G, 2000)[25], *B. rapa* and *B. oleracea*[26], KaKS_caculator[27] was used for calculating KS based on maximum likelihood and the model parameter was NG. In general, the peak of KS in intraspecific (orthologous) was considered to be related to genome duplication events, and the peak of KS in intraspecific was the divergence events. The peak of *A. thaliana* and *B. napus* were observed at 0.417, so the divergence time between *A. thaliana* and Cruciferae was approximately ~14 MY. The positive selection of K_a / K_s gene is the ratio between the heterotopic (K_a) and the homotopic (K_s). This ratio can be used to determine whether there is selection pressure acting on the protein-coding gene. If $K_a/K_s>1$, it was considered that there was a positive selection effect. If $K_a/K_s=1$, neutral selection was considered. If $K_a/K_s<1$, it was considered to have purification selectivity. K_a/K_s

value was calculated between A sub-genome of *B. napus* and *B. rapa*, C sub-genome of *B. napus* and *B. rapa*, respectively.

Identification and classification of MADS-box gene family. The gene with MADS-box domain was identified using InterProScan[28]. Using the MADS-box genes[29] in *A. thaliana* as reference, pariwise distance tool in MEGA7[30] was used to identify conserved genes in *B. napus*. Clustal W (version 2.1)[31] was used to compare the *B. napus* and *A. thaliana* MADS-box genes. MEGA7 was employed to construct a phylogenetic tree based on the comparison results with the neighbor joining (NJ) method. Parameter model was Poisson model and the bootstrap was 1000 replications.

OrthoMCL clustering. To identify and estimate the number of potential ortholog gene families between ZS11, Gangan, Zheyou7, Shengli, Tapidor, Westar, No2127 and Darmor, the OrthoMCL pipeline[32] was applied to compute the all-to-all similarities with standard settings (blastp E value $<1 \times 10^{-5}$ and inflation factor =1.5). For the un-clustered genes, considering the influence of gene length and different genome annotations, the gene sequence was mapped to other genomes to verify whether there were homologous sequences (blastn E value $<1 \times 10^{-5}$, identity>90% and coverage>90%). Finally, the orphan genes of each species were determined, and specific genes were combined into specific gene families.

Field environments and phenotype of BN-NAM population. The BN-NAM population contained 15 RIL families and a total of 2,141 RILs which was generated as previously described[33]. The RIL families were planted in the field at five different locations over a period of four years with one replication and a randomized complete block design, which included six winter environments in or near Wuhan City and two spring environments at Hezheng county, Gansu Province. The six winter environments are 14WH (grown at the experimental station at Huazhong Agricultural University in the 2013-2014 growing season), 15CD (grown in Caidian City in the 2014-2015 growing season), 15EZ, 16EZ and 17EZ (grown in Ezhou City in the 2014-2015, 2015-2016 and 2016-2017 growing season, respectively) and 15YL (grown in Yangluo City in the 2014-2015 growing season). The two spring environments are 16HZ and 17HZ (grown in Hezheng county in the 2016 and 2017 growing seasons). RIL seeds were sown in early October and harvested in late April or early May of next year, or sown in middle May

and harvested in middle September of the same year. Each RIL was grown in one plot with one row of 10 plants. The distance between plants was 20 cm within each row and the distance between rows was 30 cm. Silique length (SL) was measured as the average length of 10 siliques, and Thousand-seed weight (TSW) or seed weight (SW) was measured as the total weight of 1000 seeds. Flowering time in each environment was recorded as the number of days from the sowing to 50% plants in one plot bloomed. The SL ranged from 2.69 to 12.70 cm and the SW ranged from 1.97 to 7.65g (TSW) in five winter environments (15CD, 15EZ, 15YL, 16EZ, 17EZ), while the flowering time ranged from 46 to 174 days in the six winter environments and 37 to 114 days in two spring environments. In GWAS, the flowering time for each RIL was represented by the best linear unbiased prediction (BLUP) value of the six winter environments (W-BLUP) or by the two spring environments (S-BLUP). The BLUP values were calculated separately using an R package LME4[34].

Identification of candidate genes for flowering time. To identify candidate genes for flowering time, we first collected all genes involved in regulating flowering time in *A. thaliana*. The predicted protein sequences in the association regions in ZS11 were used as query to blast all flowering time genes in *A. thaliana*. The genes with E-value below $1e^{-5}$ were considered as orthologous candidate genes in *B. napus* (Supplementary Table 50). The association region was inferred by extending 300 kb to upstream and downstream regions from the peak SNP.

Verification of the variations in *BnaA10.FLC* using PCR. Four panels of primers were used to genotype the presence and absence of the transposable elements, MITE, hAT, LTR and LINE, in the promoter and gene regions in *BnaA10.FLC* in *B. napus* accessions. PCR products were visualized on 2% agarose gel. The primers and their corresponding products are listed in Supplementary Table 50.

Statistics. The R package Density was used to calculate the peak value of K_S by gaussian distribution fitting of the original K_S distribution. GO enrichment analysis was carried out using GOATOOLS based on Fisher's exact test[35]. Only GO terms with a *P* value of less than 0.05 were retained. REVIGO[36] was used to remove redundant GO terms. The significance thresholds for SNP-GWAS and PAV-GWAS are log (*P* value) <-4.94 and log (*P* value) <-4, respectively. Unique fragments were found on both sides of the *BnaA10.FLC* related insertion

fragment, and a total of 30 candidate sites were selected from three genomes. Then R package PCA function was used to perform Principal Component Analysis to screen the locations which contributed more to present variation in resequencing data.

Supplementary reference

1. Delcher, A. L., Salzberg, S. L. & Phillippy, A/ M. Using MUMmer to identify similar regions in large sequence sets. *Curr. Protoc. Bioinformatics* Chapter 10:Unit 10.3 (2003).
2. Chin, C. S. et al. Phased diploid genome assembly with single-molecule real-time sequencing. *Nat. Methods* **13**, 1050-1054 (2016).
3. Koren, S. et al. Canu: scalable and accurate long-read assembly via adaptive *k*-mer weighting and repeat separation. *Genome Res*. **27**, 722-736 (2017).
4. Bao, W., Kojima, K. K. & Kohany, O. Repbase Update, a database of repetitive elements in eukaryotic genomes. *Mobile Dna* **6**, 11 (2015).
5. Smit, A. F. A., Hubley, R. & Green, P. RepeatMasker Open-4.0. 2013–2015. (2015).
6. Xu, Z. & Wang, H. LTR_FINDER: an efficient tool for the prediction of full-length LTR retrotransposons. *Nucleic Acids Res*. **35**, W265-W268 (2007).
7. Edgar, R. C. & Myers, E/ W. PILER: identification and classification of genomic repeats. *Bioinformatics* **21**, i152-i158 (2005).
8. Price, A. L., Jones, N. C. & Pevzner, P. A. De novo identification of repeat families in large genomes. *Bioinformatics* **21**, i351-i358 (2005).
9. Benson, G. Tandem repeats finder: a program to analyze DNA sequences. *Nucleic Acids Res*. **27**, 573-580 (1999).
10. Stanke, M. et al. AUGUSTUS: ab initio prediction of alternative transcripts. *Nucleic Acids Res*. **34**, W435-W439 (2006).
11. Majoros, W. H., Pertea, M. & Salzberg, S. L. TigrScan and GlimmerHMM: two open source ab initio eukaryotic gene-finders. *Bioinformatics* **20**, 2878-2879 (2004).
12. SÎllner, T. et al. SNAP receptors implicated in vesicle targeting and fusion. *Nature* **362**, 318-324 (1993).
13. Birney, E., Clamp, M. & Durbin, R. GeneWise and genomewise. *Genome research* **14**, 988-995 (2004).
14. Haas, B. J. et al. Automated eukaryotic gene structure annotation using EVidenceModeler and the Program to Assemble Spliced Alignments. *Genome Biol*. **9**, R7 (2008).
15. Lowe, T. M. & Eddy, S. R. tRNAscan-SE: a program for improved detection of transfer RNA genes in genomic sequence. *Nucleic Acids Res*. **25**, 955-964 (1997).
16. Nawrocki, E. P. & Eddy, S. R. Infernal 1.1: 100-fold faster RNA homology searches. *Bioinformatics* **29**, 2933-2935 (2013).
17. Lim, Ki-Byung, et al. Characterization of rDNAs and tandem repeats in the heterochromatin of *Brassica rapa*. *Molecules & Cells* **19**, 436-444 (2005).
18. Durand, N. C. et al. Juicer provides a one-click system for analyzing loop-resolution Hi-C experiments. *Cell Syst*. **3**, 95-98 (2016).
19. Rao, S. S. P. et al. A 3D map of the human genome at kilobase resolution reveals principles of chromatin looping. *Cell* **159**, 1665-1680 (2014).
20. Knight, P. A. & Ruiz, D. A fast algorithm for matrix balancing. *IMA J. Numerical Analysis* **33**, 1029-1047 (2013).
21. Wang, M. et al. Evolutionary dynamics of 3D genome architecture following polyploidization in cotton. *Nat. Plants* **4**, 90 (2018).
22. Wang, X. et al. The genome of the mesopolyploid crop species *Brassica rapa*. *Nat. Genetics* **43**, 1035 (2011).
23. Wang, Y. et al. MCScanX: a toolkit for detection and evolutionary analysis of gene synteny and collinearity. *Nucleic Acids Res*. **40**, e49 (2012).

24. Zhang, Z. et al. ParaAT: a parallel tool for constructing multiple protein-coding DNA alignments. *Biochem. Bioph. Res. Co.* **419**, 779-781 (2012).
25. Initiative, A. G. Analysis of the genome sequence of the flowering plant *Arabidopsis thaliana*. *Nature* **408**, 796-815 (2000).
26. Belser, C. et al. Chromosome-scale assemblies of plant genomes using nanopore long reads and optical maps. *Nat. Plants* **4**, 879-887 (2018).
27. Zhang, Z. et al. KaKs_Calculator: calculating Ka and Ks through model selection and model averaging. *Genomics Proteomics Bioinformatics* **4**, 259-263 (2006).
28. Quevillon, E. et al. InterProScan: protein domains identifier. *Nucleic Acids Res.* **33**, W116-W120 (2005).
29. Pařenicová, L. et al. Molecular and phylogenetic analyses of the complete MADS-box transcription factor family in Arabidopsis: new openings to the MADS world. *Plant Cell* **15**, 1538-1551 (2003).
30. Kumar, S., Stecher, G. & Tamura, K. MEGA7: molecular evolutionary genetics analysis version 7.0 for bigger datasets. *Mol. Biol. Evol.* **33**, 1870-1874 (2016).
31. Larkin, M. A. et al. Clustal W and Clustal X version 2.0. *Bioinformatics* **23**, 2947-2948 (2007).
32. Li, L., Stoeckert, C. J. & Roos, D. S. OrthoMCL: identification of ortholog groups for eukaryotic genomes. *Genome Res.* **13**, 2178-2189 (2003).
33. Hu, J. et al. Genetic properties of a nested association mapping population constructed with semi-winter and spring oilseed rapes. *Front. Plant Sci.* **9**, 1740 (2018).
34. Bates, D. et al. The lme4 package. *R package version*, **2**, 74 (2007).
35. Klopfenstein, D. V. et al. GOATOOLS: A Python library for Gene Ontology analyses. *Sci. Rep.* **8**, 10872 (2018).
36. Supek, F. et al. REVIGO summarizes and visualizes long lists of gene ontology terms. PloS One **6**, e21800 (2011).
