# Supplementary material for: Eight high-quality genomes reveal pan-genome architecture and ecotype differentiation of Brassica napus
Source: Nat Plants. 2020 Jan 13;6(1):34–45. doi: 10.1038/s41477-019-0577-7 (PMC6965005; doi:10.1038/s41477-019-0577-7)
Supplement: Supplementary file 2 — Reporting Summary [file 41477_2019_577_MOESM2_ESM.pdf]

nature research

Corresponding author(s): Kede Liu, Qing-Yong Yang, Ling-Ling Chen, Liang Guo

Last updated by author(s): Nov 17, 2019

Reporting Summary

Nature Research wishes to improve the reproducibility of the work that we publish. This form provides structure for consistency and transparency in reporting. For further information on Nature Research policies, see Authors & Referees and the Editorial Policy Checklist.

Statistics

For all statistical analyses, confirm that the following items are present in the figure legend, table legend, main text, or Methods section.

n/a	Confirmed	
☐	☒	The exact sample size (*n*) for each experimental group/condition, given as a discrete number and unit of measurement
☐	☒	A statement on whether measurements were taken from distinct samples or whether the same sample was measured repeatedly
☐	☒	The statistical test(s) used AND whether they are one- or two-sided<br>*Only common tests should be described solely by name; describe more complex techniques in the Methods section.*
☐	☒	A description of all covariates tested
☐	☒	A description of any assumptions or corrections, such as tests of normality and adjustment for multiple comparisons
☐	☒	A full description of the statistical parameters including central tendency (e.g. means) or other basic estimates (e.g. regression coefficient) AND variation (e.g. standard deviation) or associated estimates of uncertainty (e.g. confidence intervals)
☐	☒	For null hypothesis testing, the test statistic (e.g. *F*, *t*, *r*) with confidence intervals, effect sizes, degrees of freedom and *P* value noted<br>*Give P values as exact values whenever suitable.*
☒	☐	For Bayesian analysis, information on the choice of priors and Markov chain Monte Carlo settings
☒	☐	For hierarchical and complex designs, identification of the appropriate level for tests and full reporting of outcomes
☐	☒	Estimates of effect sizes (e.g. Cohen's *d*, Pearson's *r*), indicating how they were calculated

Our web collection on statistics for biologists contains articles on many of the points above.

Software and code

Policy information about availability of computer code

Data collection	PacBio Sequel, PacBio SMRT Link, IrysSolve
Data analysis	BLASR (version 1.6), Falcon (version 2017.11.02-16.04-py2.7), SMRT Link (version 5.0.1), SAMTools (version 0.1.19), BWA (version 0.7.8-r455), Pilon (version 1.18), MUMmer (version 3.23), 3D-DNA (version 180419), Juicebox (version 1.8.8), BLAST+ (version 2.2.28), MAFFT (version 7.427), Gblocks (version 0.91b), RAxML (version 8.0.0), PLINK (version 1.90), TreeBeST (version 1.9.2), iTOL (version 3), GCTA (version 1.91.7), SyRI (https://github.com/schneebergerlab/syri), minimap2 (version 2.10), MCscanx (20130912), GEMMA (version 0.98), BayesTyper (version 1.3.1), KMC (version 3.1.0), R (version 3.5.3), LTR_FINDER (version 1.05), PILER (version 1.0), RepeatScout (version 1.0.5), RepeatModeler (version 1.0.11), TRF (version 4.09), RepeatMasker (version 4.0.6), Augustus (version 2.4), GlimmerHMM (version 3.0.4), SNAP (20130216), GenBlastA (version 1.0.4), GeneWise (version 2.4.1), PASA (version 2.0.2), EVM (version 1.1.1), BUSCO (version 2), tRNAscan-SE (version 1.3.1), Infernal (version 1.1.2), GATK (version 3.8.1), KaKs_Calculator (version 2.0), InterProScan (version 5), TopHat (version 2.0.13), Cufflinks (version 2.2.1), MEGA (version 7.0), Clustal W (version 2.1), OrthoMCL (version 2.0.9), GOATOOLS (version 0.6.10), REViGO, BEDTools (version 2.13.3), Bionano Solve (version 3.4.1), IrysView (version 2.5.1), Canu (version 1.6), Juicer (version 1.6.2), Circos (version 0.69-6), GEC (version 0.2).

For manuscripts utilizing custom algorithms or software that are central to the research but not yet described in published literature, software must be made available to editors/reviewers. We strongly encourage code deposition in a community repository (e.g. GitHub). See the Nature Research guidelines for submitting code & software for further information.

Data

Policy information about availability of data

All manuscripts must include a data availability statement. This statement should provide the following information, where applicable:

- Accession codes, unique identifiers, or web links for publicly available datasets
- A list of figures that have associated raw data
- A description of any restrictions on data availability

Provide your data availability statement here.

Field-specific reporting

Please select the one below that is the best fit for your research. If you are not sure, read the appropriate sections before making your selection.

☒ Life sciences ☐ Behavioural & social sciences ☐ Ecological, evolutionary & environmental sciences

For a reference copy of the document with all sections, see nature.com/documents/nr-reporting-summary-flat.pdf

Life sciences study design

All studies must disclose on these points even when the disclosure is negative.

Sample size	The sample size of 210 is the number of rapeseed accessions collected from the world major rapeseed growing countries to represent the genetic diversity of rapeseed, 141 of which had ecotype information. The sample size of 2,141 is the number of inbred line of our association mapping population BN-NAM. The predetermine sample size of BN-NAM includes 15 RIL populations, each RIL population includes 160 individuals; however, only 2141 individuals contain complete genotype and phenotype data in total.
Data exclusions	During association mapping, those variants with minor allele frequency (MAF) less than 5% were excluded. This is common applied since the current method has no power to uncover this kind of rare alleles in maize, rice (Li et al., 2016; Yano et al., 2016; Si et al., 2016).
Replication	For RNA-Seq experiment of the leaves of eight accessions, we used at two biological replicates. For the gene expression in leaves in each stage of eight accessions the replication was highly successful.
Randomization	Plant growth of rapeseeds was in a randomized block design.
Blinding	For the 210 rapeseed accessions, 69 rapeseed accessions had no unequivocal ecotype information since the other 141 rapeseed accessions containing three different ecotypes of B. napus make sence in further analysis.

Reporting for specific materials, systems and methods

We require information from authors about some types of materials, experimental systems and methods used in many studies. Here, indicate whether each material, system or method listed is relevant to your study. If you are not sure if a list item applies to your research, read the appropriate section before selecting a response.

Materials & experimental systems

n/a	Involved in the study
☒	☐ Antibodies
☒	☐ Eukaryotic cell lines
☒	☐ Palaeontology
☒	☐ Animals and other organisms
☒	☐ Human research participants
☒	☐ Clinical data

Methods

n/a	Involved in the study
☒	☐ ChIP-seq
☒	☐ Flow cytometry
☒	☐ MRI-based neuroimaging
